# Investigating the methodological foundation of lesion network mapping

**DOI:** 10.1038/s41593-025-02196-7

**Published:** 2026-01-15

**Authors:** Martijn P. van den Heuvel, Ilan Libedinsky, Sebastian Quiroz Monnens, Jonathan Repple, Iris Sommer, Luca Cocchi

**Affiliations:** 1 Department of Neurosciences, Center for Neurogenomics and Cognitive Research, Amsterdam Neuroscience, Vrije Universiteit Amsterdam, Amsterdam, the Netherlands; 2 Department of Child and Adolescent Psychiatry and Psychology, Amsterdam UMC location Vrije Universiteit Amsterdam, Amsterdam, the Netherlands; 3 Department of Psychiatry, Psychosomatic Medicine and Psychotherapy, Goethe University Frankfurt, University Hospital, Frankfurt, Germany; 4 Institute for Translational Psychiatry, University of Münster, Münster, Germany; 5 Goethe University Frankfurt, Cooperative Brain Imaging Center—CoBIC, Frankfurt, Germany; 6 Center for Clinical Neuroscience and Cognition, University Medical Center Groningen, Groningen, the Netherlands; 7 Brain and Mental Health Program, QIMR Berghofer, Brisbane, Queensland Australia; 8 School of Biomedical Sciences, University of Queensland, Brisbane, Queensland Australia

**Keywords:** Psychiatric disorders, Systems biology

## Abstract

Lesion network mapping (LNM) is a neuroimaging framework that uses normative functional connectivity (FC) data to link heterogeneous brain lesions and functional alterations to brain networks implicated in neurological and psychiatric conditions. However, many of the networks identified by LNM and related methods appear to be highly similar across diverse conditions such as addiction, depression, psychosis and epilepsy. To understand this similarity, we re-examined the data from multiple LNM studies and assessed the methodological roots of the method. Our findings reveal a foundational limitation: at its core, LNM involves a repetitive sampling of one and the same FC matrix. As a result, it systematically maps sets of local brain changes—whether they are patient lesions, magnetic resonance imaging-derived alterations, synthetic or random—onto the same nonspecific properties of the used FC data, producing highly similar networks across conditions. This central limitation cautions the use of LNM as a method for studying distinct biological networks underlying brain disorders. Our work may aid the development of a new generation of network-mapping methods from first principles.

## Abstract (web summary)

The lesion network mapping method links diverse brain lesions to similar functional brain networks, reflecting general brain organization rather than disorder-specific circuits.

## Main

Identifying brain regions and circuits that give rise to neurological and psychiatric symptoms is a central goal of fundamental and clinical neuroscience. Charting the relationship between brain alterations and behavior has long served as a cornerstone of this effort, from linking brain injury to behavioral outcomes^1–3^ to systematic studies leveraging modern neuroimaging techniques^4^. Progress has, however, been more elusive for complex neurological and psychiatric conditions, where patients can often exhibit highly spatially distributed and heterogeneous brain abnormalities^5–7^.

The method of ‘lesion network mapping’ (LNM)^8,9^, also known in literature under alternative terms such as ‘causal brain mapping’^10^, ‘causal network localization’^11^, ‘lesion network-symptom mapping’^12–15^, ‘network localization’^16,17^, ‘atrophy network mapping’^18^, ‘remission network mapping’^19^, ‘coordinate network mapping’ or ‘coordinate-based network mapping’^20–22^, ‘activation network mapping’^23^, ‘network-based meta-analytic’ analysis^24^, among others (Supplementary Table 1), has rapidly gained traction as a framework to trace and unite topographically heterogenous lesions and other brain alterations to underlying brain circuits^10,11,15^. Collectively referred to as the LNM framework, this method maps the anatomical locations of brain alterations onto normative functional brain connectivity (FC) to examine whether, and if so how, these alterations converge onto a common underlying network. The framework posits that alterations in different brain regions can give rise to similar clinical symptoms when they disrupt the same functional brain network. Over the past years, LNM studies have reported such functional networks for a broad range of neurological and psychiatric disorders, including post-traumatic stress disorder (PTSD)^25^, epilepsy^26,27^, autism spectrum disorder (ASD)^28^, schizophrenia^29^, obsessive-compulsive disorder (OCD)^30^ and migraine^20^, among many others (see refs. ^31–33^ and a 2025 PubMed/ClinicalTrials.gov search for review; Supplementary Table 1 and Supplementary Note 1). Notable LNM findings include the ‘causal depression network’^15,34–36^, a ‘psychosis circuit’^37^ and brain circuits related to addiction^38^, all highlighted as promising for clinical application^15,25,26,38–40^.

**Fig. 1:**
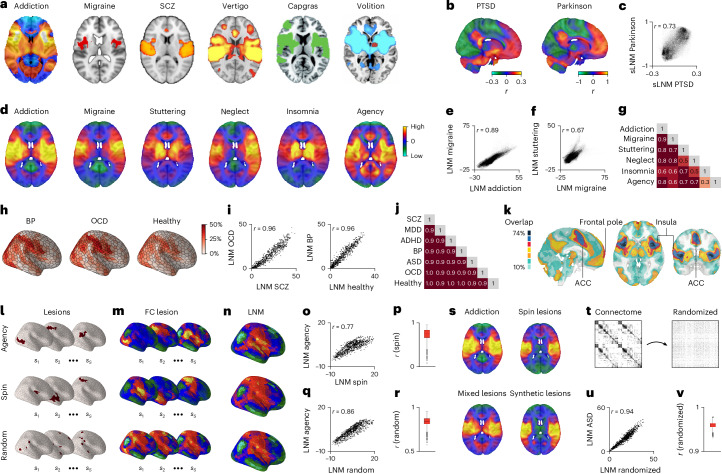
Observed similarity of published work using LNM networks from original and randomized lesions. **a**,**b**, Images of LNM-related circuitry maps from recent LNM and sLNM publications (from refs. ^16,20,25,29,38,41–43^). Panel **a** is reproduced with permission. **c**, Correlation between sLNM networks for reduced PTSD risk^25^ and cognitive decline induced by DBS in Parkinson’s disease^43^ (shown in **b**). **d**, Recomputed LNM maps resulting from the application of voxel-wise Lead-DBS^54^ on publicly available lesions for addiction^38^, migraine^20^, neurogenic stuttering^44^, neglect syndrome^53^, insomnia^53^ and disrupted agency^16^. Reconstruction of LNM maps (**d**, first two images) compared to those reported in the original study (**a**) is high. **e**–**g**, Correlations between reconstructed LNM maps depicted in **d** are shown. **h**–**j**, Results show high similarity between LNM circuits derived from cortical deviations for six psychiatric conditions (BP and OCD are shown) and healthy controls; data taken from ref. ^28^. **k**, The most reported regions across 102 LNM networks from a literature survey (Supplementary Tables 1 and 2), highlighting the prevalence of the top 10% highest correlated and anticorrelated voxels. Extensive overlap is evident in the insula, ACC and frontal pole. **l**–**n**, LNM networks derived from random lesions also show highly similar LNM outcomes. For example, lesions that disrupted agency^16^ and spin-randomized versions of these lesions (middle row) across the brain, as well as completely randomized seed locations (bottom row), result in similar LNM outcomes (shown in **n**). **o**,**q**, Plot of the spatial correlation between the original LNM map (disrupted agency^16^) and a typical example from the randomized conditions. **p**,**r**, Randomization of lesions was repeated 1,000 times, with almost all occasions resulting in highly similar LNM maps between the original (disrupted agency) and random conditions (box plots show values of *n* = 1,000 permutations; (**p**) minima = 0.06, maxima = 0.92, center = (median) 0.75, bounds of box (Q1 25th percentile–Q3 75th percentile) = 0.66–0.81, whiskers = 0.43–0.92; (**r**) minima = 0.58, maxima = 0.96, center = (median) 0.84, bounds of box (Q1 25th percentile–Q3 75th percentile) = 0.81–0.87, whiskers = 0.72–0.96). **s**, The application of LNM (Lead-DBS) on lesions associated with addiction remission (top left, lesion masks taken from ref. ^38^). The panel also shows LNM outputs on the same lesion set but now spin-randomized across the cortex (top right, exemplary spin, *r* = 0.48), following a random selection of 100 lesions with mixed symptomatology (bottom left, ‘mixed lesions’, *r* = 0.93), and based on 100 synthetic lesions (bottom right, *r* = 0.71). All approaches yield very similar LNM maps. **t**–**v**, Plots show data (ASD^28^) from an alternative null analysis, with the connections of the group connectome *C* binarized and randomized (**t**, left = original matrix, right = randomized matrix). Once again, LNM analyses resulted in very similar maps. Plot in **u** shows a representative example (ASD) and **v** shows a box plot of all randomizations (box plot shows values of *n* = 1,000 permutations; minima = 0.93, maxima = 0.98, center = (median) 0.96, bounds of box (Q1 25th percentile–Q3 75th percentile) = 0.96–0.96, whiskers = 0.94–0.98). ADHD, attention-deficit/hyperactivity disorder; BP, bipolar disorder; MDD, major depressive disorder; OCD, obsessive-compulsive disorder; PTSD, post-traumatic stress disorder; *s* subjects; SCZ, schizophrenia.

However, many of these reported LNM networks—purportedly delineated as disease-specific—seem to converge on strikingly similar brain networks. As illustrated in Fig. 1a,b, the LNM networks reported for psychiatric conditions such as addiction^38^, migraine^20^, PTSD^25^ and schizophrenia^29^, but also for neurological conditions such as vertigo^41^, Capgras syndrome^42^, Parkinson’s disease^43^ and disrupted volition^16^, appear to implicate one and the same system, a network involving bilateral insular cortices, the anterior cingulate cortex (ACC) and parts of the frontopolar cortex, thalamus and cerebellum. This observation is unexpected, considering the substantial heterogeneity in etiology and symptomatology of these conditions.

Examining this spatial overlap between published LNM networks in more detail substantiates the observed high spatial alignment. For example, published LNM networks for PTSD^25^ and cognitive decline in Parkinson’s disease^43^ show high spatial correlation (*r* = 0.73; see Fig. 1b,c, Supplementary Note 2 and Supplementary Table 2 for data sources). Similar overlap is observed among networks for addiction^38^, migraine^20^, neurogenic stuttering^44^ and disrupted agency^16^ (*r* = 0.62–0.89; voxel-wise *P* < 0.001; Fig. 1d–g). This spatial alignment remains highly significant after correcting for spatial autocorrelation effects (spin test^45^ and BrainSMASH^46^; *P*_spin_, *P*_brainsmash_ < 0.001; *r* and *P* values for all examined networks are listed in Supplementary Table 3). Similar overlap is evident for LNM networks linked to aphasia^47^ and epilepsy^27^ (*r* = 0.40), amnesia^48^ and psychosis^37^ (*r* = 0.80), as well as for networks further linked to individual symptom data like networks related to risk of depression in multiple sclerosis^34^ and remission for smoking addiction^38^ (*r* = 0.57; all *P*, *P*_spin_, *P*_brainsmash_ < 0.001). LNM maps derived based on focal neurological lesions (for example, dyskinetic cerebral palsy^49^) or associated with deep brain stimulation (DBS)-related targets (for example, treatment for OCD^50^) also appear to show surprisingly high similarity (*r* = 0.64; *P*, *P*_spin_, *P*_brainsmash_ < 0.001; Supplementary Table 3).

Remarkably, several of these LNM networks—for example, disruption of agency^16^ (Fig. 1l–n), ASD^28^, addiction^38^, but also epilepsy^27^ (Supplementary Fig. 8)—seem to be indistinguishable from networks derived when lesions are randomly shuffled across the brain (*r* = 0.73–0.95; Fig. 1l–r), derived from a mix of lesions not associated with one specific disorder (Fig. 1s), or even from completely random synthetic lesions (Fig. 1s and Supplementary Note 6). Also, randomizing the connections of the normative connectome dataset does not appear to markedly disrupt the LNM outcomes, resulting in rather similar networks (degree-preserving randomization^51,52^; for example, LNM for neglect syndrome^53^, *r* = 0.66, addiction^38^, *r* = 0.72, agency^16^, *r* = 0.75, and ASD^28^, *r* = 0.94, illustrated in Fig. 1t–v; Supplementary Note 7 and Supplementary Fig. 3).

The breadth of this spatial similarity is indicated by a literature survey, identifying 201 studies that discussed and/or used the LNM framework in context of studying 101 neurological and psychiatric conditions (2015–2025; see details in Supplementary Notes 1 and Supplementary Table 1). Re-analyzing 102 LNM networks across 72 of these studies confirmed an overall high alignment of LNM maps (|*r*| = 0.40, s.d. = 0.25; Supplementary Notes 2 and 3), with regions such as the bilateral insula, ACC and frontal cortex appearing in up to 74% of reported LNM networks (Fig. 1k; see Supplementary Note 5 for details).

To explain this notable similarity among reported LNM networks, we examined the core principles of the method. Our systematic analysis reveals a fundamental limitation of LNM methods: LNM projects sets of lesions—regardless of their clinical association—onto only elementary properties of the standard connectivity matrix, primarily the row sum of that matrix (that is, node ‘degree’). Below, we provide a step-by-step walkthrough of the LNM pipeline, illustrating how its procedural stages can be expressed compactly in linear matrix notation. This formalization exposes the inherent constraint of the method that explains why the majority of published LNM networks converge to highly similar outcomes instead of identifying disorder-specific circuits.

## Results

### Step-by-step walkthrough of LNM

**Fig. 2:**
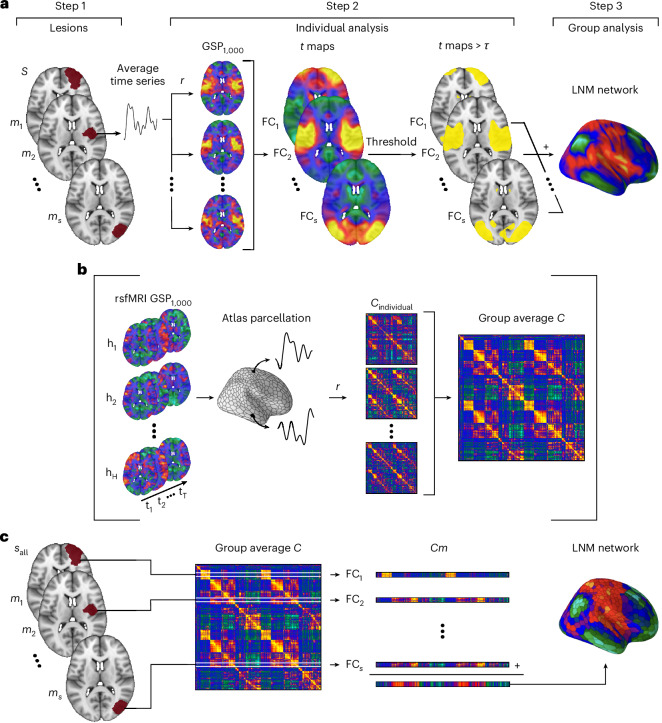
LNM pipeline and streamlined implementation. **a**, The procedure of LNM involves three major steps—first, the lesion(s) of a single patient *s* (step 1) is placed into standard space. Next, the FC profile of that lesion *m*_*s*_ of patient *s* is computed by means of the fMRI resting-state data in a large normative dataset, with the FC maps combined in a one-sample *t* test (two-sided) to obtain a single FC map for each lesion of patient *s*. Optionally, the *t* map can be thresholded to select the strongest connections (step 2). Steps 1 and 2 are repeated for all lesions of the group of patients *S*. Afterwards, the individual FC lesion maps are combined in a group analysis (step 3) to define their underlying common network. **b**, Step 2 of the LNM procedure can be streamlined (left, middle row) using an atlas-based approach in which the cortex and subcortical areas are parcellated according to a high-resolution atlas—for example, the Yeo-Schaefer1000/Melbourne54 atlas^107,108^. Middle, an atlas-based approach allows for precomputation of all lesion-to-region FC for all datasets in the normative connectome dataset. Right, all individual matrices can be grouped into a single group connectome *C*, with the resulting group matrix containing the same information as the one-sample *t* test performed in step 2. **c**, Taken together, the entire LNM procedure is now compressed to selecting row *i* corresponding to lesion *m*_*s*_ of patient *s* from the group matrix *C* (optionally, threshold the resulting vector), repeat this for all lesions of all patients *s* in *S*, and summing over the selected rows *Cm* to obtain the final LNM network map. *C*, group connectivity matrix; GSP1000, Brain Genomics Superstruct Project 1000; h, normative participants; *r*, correlation coefficient; *S*, all participants.

LNM (for methodologically equivalent variants and approaches published under different nomenclature, see Supplementary Table 1, from now on collectively referred to as LNM) typically consists of three methodological steps. Figure 2a presents a schematic of these steps, as implemented in popular LNM toolboxes like Lead-DBS^54^ (Supplementary Notes 8 and 17). We can consider a group of patients, each with one or more brain lesions, and study them using a large standard resting-state functional magnetic resonance imaging (fMRI) dataset from normative healthy individuals (for example, 1,000 healthy participants from the GSP1000 (ref. ^55^) or Human Connectome Project^56^). In step 1 of the LNM procedure, each lesion is mapped to corresponding voxels in the standardized space (for example, MNI152) of the normative dataset. Next, in step 2, the FC of a lesion is computed by correlating the average resting-state time series of the lesion’s matching voxels with all other voxels in the brain and standardizing the correlation values using a Fisher *r*-to-*z* transformation. This is repeated across all healthy datasets in the normative connectivity dataset, resulting in over 1,000 FC maps per lesion, which are then combined into a single map using a one-sample *t* test to assess voxel-wise deviation from zero FC. A threshold (for example, |*t*| > 7) can be applied to identify the strongest connections^57^. Steps 1 and 2 are repeated for all studied lesions, producing a set of individual FC *t* maps, one for each lesion.

Next, in the group-analysis step 3 of the LNM procedure, the lesion FC *t* maps are combined to produce the group LNM network. This is typically done by averaging the lesion FC *t* maps, identifying regions consistently connected across lesions (for example, ≥75% (ref. ^17^)). The resulting map is referred to as the LNM network^9^ or LNM sensitivity map^8^. Alternatively, when individual symptom data are available, the group-analysis step 3 can involve correlating the lesion FC maps with symptom scores (~16% of reviewed studies; Supplementary Table 1) or contrast subgroups with differing symptom levels (~11%); variants of the method referred to as ‛lesion network-symptom mapping’ or symptom-based LNM^12–15^. The sign of the resulting *r* values or *t* values in the symptom lesion network mapping (sLNM) depends on the behavioral scale that is used, and may indicate, for example, risk level^11,25^, symptom change^15^ or clinical state (for example, relapse versus remission)^38^.

### Formal notion of LNM

We found that the LNM methodological steps can be considerably compressed, without losing information. This compression is illustrated in Fig. 2b,c, and a mathematical derivation is provided in Supplementary Note 18. First, precomputing the correlation among the time series of all brain voxels yields all possible lesion-to-voxel FC maps beforehand. These precomputed matrices, for all normative participants in the normative connectivity dataset (*H*), can replace step 2 in the LNM approach (Fig. 2b). To improve practical feasibility, a high-resolution brain atlas can be used to divide the brain into, for example, *R* = 1,000 equally sized regions^58^. Furthermore, inferring equal variance across the connections in *H* (which we empirically validated, *r* = 0.99; Supplementary Note 8), the one-sample *t* test in step 2 can be replaced by taking the mean of the precomputed individual matrices^54^. This allows replacing the entire set of 1,000 normative FC matrices with a single mean group connectivity matrix *C* (Fig. 2b). This approach eliminates the need for looping the procedure over all normative datasets for each lesion, repetitively, reducing the computation time for a standard dataset of 50 lesions from ~10–12 h using the Lead-DBS toolbox^54^ to under 10 s. We empirically validated this compressed approach, with both the full Lead-DBS implementation and the atlas-based accelerated version producing effectively identical LNM maps (examined across 100 patient and 100 synthetic lesions, mean *r* = 0.96; Supplementary Notes 8 and 20).

The compressed version (Fig. 2c) describes the LNM procedure now as: (step 1) matching lesion *m*_*s*_ of participant *s* to the region(s) *i* in the used brain atlas; (step 2) selecting the matching row(s) *i* in the group connectivity matrix *C*; repeat steps 1–2 for all lesions; and (step 3 group analysis) taking the sum (or mean, which are equivalent) of all selected rows to obtain the final LNM map.

Formally, we can express LNM as

1 $$\mathrm{LNM}=\mathop{\sum }\limits_{s=1}^{{S}}\left(\frac{1}{|{m}_{s}|}\mathop{\sum }\limits_{i\in {m}_{s}}{C}_{i,r}\right)\mathrm{for}\,\mathrm{all}\,r\in R$$

where *S* denotes the total set of patients, *s* one specific participant, *m*_*s*_ the lesion of participant *s*, |*m*_*s*_| the size of lesion *m*_*s*_, *i* the row(s) in *C* matching the region(s) of lesion *m*_*s*_ in participant *s*, *C* the group average functional matrix of size *R* × *R, R* all voxels or brain regions in the chosen brain mask or atlas and *r* a specific region in *R* (scaled with a fixed constant; for exact formal notation, see Supplementary Notes 8 and 18). We can also rewrite equation (1) in a vector notation:

2 $$\begin{array}{l}{\mathrm{LNM}}=\mathop{\sum}\limits_{s=1}^{{S}}\left({{{\bf{m}}}}_{s}\times C\right)\end{array}$$

where ${{\bf{m}}_{s}}$ is a row vector of size 1 × *R*, indicating the lesion region with entries of 1 or 1/$\left|{m}_{s}\right|$ when a lesion covers multiple rows, and 0 otherwise.

We can now make one final compression—combining all lesion vectors ${{\bf{m}}}_{s}$ of all participants into a single lesion matrix *M* = (${\vec{{\bf{m}}}}_{1}$, ${\vec{{\bf{m}}}}_{2}$, …,${{\vec{\bf{m}}}}_{s}$) (Fig. 2c). This summarizes the entire LNM procedure (steps 1, 2 and 3 combined) to a linear matrix multiplication:

3 $$\mathrm{LNM}=\mathop{\sum }\left(M\times C\right)$$

where *M* denotes the lesion matrix, *C* the standard group connectivity matrix.

In the sLNM variant, the group-analysis step is slightly modified (illustrated in Supplementary Fig. 1). In step 3, at each voxel, the FC values across the individual lesion maps (size *S* × 1) are further correlated with the participants’ symptom scores (size *S* × 1), instead of taking the mean over all maps without further weighting. With steps 1 and 2 the same (and given by *M* × *C*, equation (3)), it can be obtained that the calculation of the final sLNM *r* map of all voxels in step 3 scales with:

4 $$\mathrm{sLNM}=\mathop{{\bf{sv}}}\limits\times \left(M\times C\right)$$

where *M* and *C* are again the lesion matrix and the normative group connectivity matrix, and ${{\bf{sv}}}$ now a standardized row vector describing the individual symptom scores (Supplementary Notes 9 and 19 provide a step-by-step and more formal derivation of sLNM).

We provide exemplary code for the voxel-wise Lead-DBS implementation of LNM and sLNM, along with the equivalent linear matrix form of equations (3) and (4) in Supplementary Note 20.

### LNM converges to the elementary properties of the input matrix

The above formal characterization brings to light a key limitation at the core of the LNM method, explaining the observed similarity between published networks (Fig. 1). Specifically, the approach involves a repetitive sampling of one and the same matrix *C*, with the lesions *M* (and additionally the symptom scores *sv* in the sLNM variant) involving only linear operations on the input matrix.

**Fig. 3:**
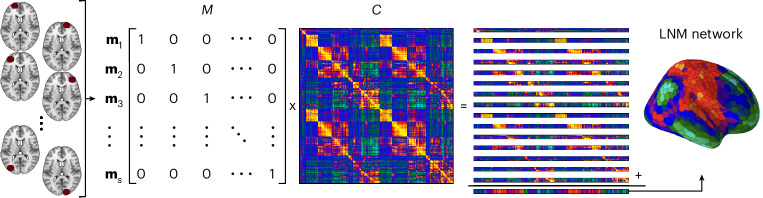
The systematic alignment of LNM to the summation vector of C. Visual illustration of how the method of LNM represents a matrix multiplication *M* × *C. M* is the lesion matrix containing the full lesion information across all participants *S*. Each row defines a unique lesion vector **m**_*s*_ describing the brain region(s) affected by the lesion(s) of participant *s* (1) and which are not (0). *C* is the normative functional connectivity matrix of size *R* × *R*. The LNM procedure samples the corresponding rows of the normative matrix *C*. In the case of the number of lesions to approximate all regions of the brain, *M* becomes the identity matrix *I*, leading to the entire LNM procedure to copy *C*. After (optional) thresholding and summing across rows, the resulting LNM map equals the summation vector, or degree, of the normative connectome *C*. It is readily obtained that this alignment to degree will also occur when sets are smaller in size than *R*, with a uniform sampling of *C* approximating the degree of the matrix.

Let us consider two simple cases. First, for a single patient with exactly one unifocal lesion, applying LNM yields an intermediate tensor (equation (1)) of size *S* × *M* × *R* = 1 × 1 × 1,000. Averaging over lesions *M* of participants *S* (both equal to 1 here) results in an LNM brain map that mirrors row *i* of the input matrix *C*. Similarly, with five distinct lesions across five patients, LNM selects five rows from *C*, and the resulting LNM map corresponds to the sum or mean of those rows. Now consider a larger sample of *S* » 1 participants, each with a single lesion (Fig. 3). For *S* = 1,000 with minimal spatial overlap between lesions, each lesion approximately corresponds to a unique region in the set of *R* = 1,000 regions, and thus to a unique row of *C*. It now emerges that step 2 of the LNM procedure involves selecting all rows of *C*, effectively reproducing the entire matrix. In the group-analysis step 3, the resulting LNM map contains the same information as the row-summation vector of the original connectivity matrix *C*. This convergence to the row-summation vector of *C* is even clearer when viewed in matrix notation (equation (3)). In this example, the lesion matrix *M* is the identity matrix *I*, leaving steps 1 and 2 as *I* × *C*, and the final group-analysis map as the row-summation vector of *C* (Fig. 3).

Such convergence arises rapidly for any reasonably sized set of spatially heterogeneous lesions, which represent the typical input to LNM studies (Supplementary Table 1). When LNM (equation (3)) is applied to lesion sets of ≥10 spatially heterogeneous lesions, the resulting map already approximates the summation vector of *C* (Supplementary Fig. 2; *r* > 0.44, 10,000 runs, *P*_spin_ < 0.05). For sets of 20–25 heterogeneous lesions, a typical size for LNM studies (Supplementary Table 4), the correlation increases further quickly (*r* > 0.62; Supplementary Fig. 2), approaching the degree distribution of the input matrix for almost all spatially heterogeneous lesion sets.

This systematic alignment with the summation vector of *C* also occurs when lesions exhibit substantial spatial overlap. Although most LNM studies focus on spatially heterogeneous lesion sets (for example, refs. ^8,22,24,28,38^; Supplementary Table 1), some have examined localized, overlapping lesions—for example, localized stroke or other lesion data linked to peduncular hallucinosis^8^, coma^59^, psychosis^37^, as well as spatially proximal transcranial magnetic stimulation (TMS) or DBS stimulation sites^43,50,60^. In these cases (with empirical examples reported below), the lesion vectors in matrix *M* contain duplicates or mark rows of *C* corresponding to spatially adjacent regions, resulting in the repeated selection of identical or highly similar rows. Consequently, the resulting LNM map still converges to the sum of the selected rows, primarily reflecting the inherent FC pattern of the underlying seed region(s). Even in the extreme case where all lesions fall within a single region, the probability that the LNM map reflects the degree structure of *C* remains non-negligible (|*r*| > 0.3, 74% of all possible cases; Supplementary Note 10). More formally, in such scenarios, the LNM map converges toward the sum of the row-induced subgraph *Cm* of *C*, that is, the sum of rows corresponding to the lesion regions (*i*, *j*, …, *k*).

Variants like sLNM refine the LNM map using individual symptom scores, but they still fundamentally rely on information drawn from one and the same connectivity matrix *C*. The linear operation of a vector, such as the symptom/phenotype vector ***sv*** on a structured (formally, low-rank) matrix, will produce patterns of correlation *r* values that are shaped by the limited set of latent factors defining the matrix (we provide a more detailed explanation of this phenomenon together with examples in Supplementary Note 9). Consequently, sLNM maps based on a structured matrix, such as the FC matrix *C*, will align with the elementary properties of *C*. This leaves systematic traces in the sLNM map, most strongly aligned with the dominant latent factors of *C* (for example, PC1 of *C*, which overlaps with degree, |*r*| = 0.82), resulting in predictable sLNM outcomes regardless of whether the lesions or symptom scores are clinically informed or random (Supplementary Notes 9 and 20).

### Empirical LNM results systematically reflect the summation vector of the connectivity matrix

**Fig. 4:**
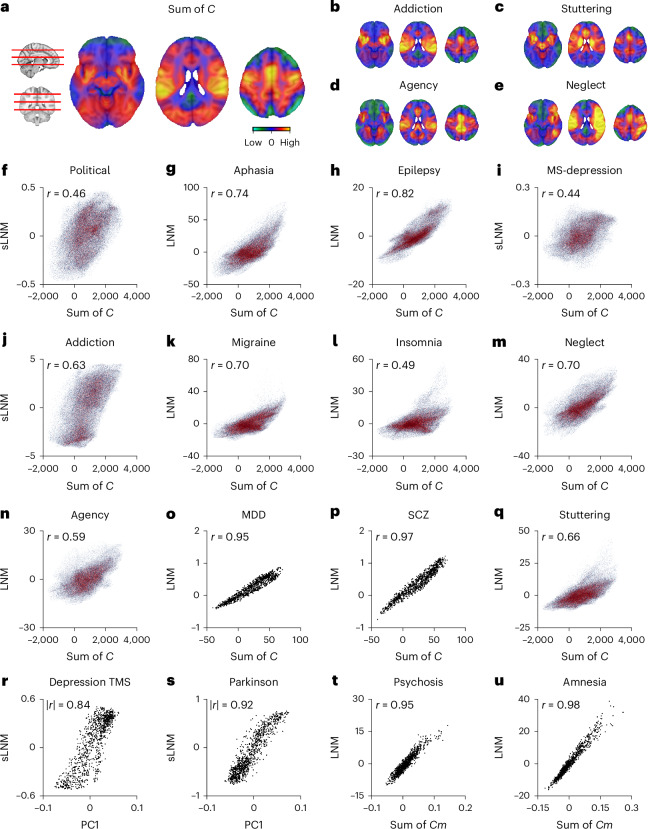
Published LNM networks converge to the summation vector of the connectome data. **a**, Brain plots displaying the degree of the group average functional connectome in standard space, with warmer colors indicating regions of high degree. **b**–**e**, Same slices as in **a** for LNM maps for addiction^38^ (**b**), neurogenic stuttering^44^ (**c**), disrupted agency^16^ (**d**) and neglect syndrome^53^ (**e**). **f**–**q**, Correlations between functional degree of the normative connectome and (s)LNM networks (from left to right) for political involvement^109^ (**f**), aphasia^62^ (**g**), epilepsy^27^ (**h**), depression circuit in multiple sclerosis (MS-depression)^34^ (**i**), addiction^38^ (**j**), migraine^20^ (**k**), insomnia^53^ (**l**), neglect syndrome^53^ (**m**), disrupted agency^16^ (**n**), major depressive disorder (MDD)^28^ (**o**), schizophrenia (SCZ)^28^ (**p**) and neurogenic stuttering^44^ (**q**). Red dots represent voxels, black dots denote brain regions (atlas-based LNM). **r**,**s**, Systematic relationship between the first principal component (PC1) of the normative connectome and LNM maps derived from sLNM^12^, a variant of LNM in which lesion functional maps are further tuned by correlating them to individual symptom scores, for TMS target sites for depression^64^ (**r**), and DBS-related networks for cognitive decline in Parkinson’s disease^43^ (**s**). **t**,**u**, Association between LNM maps and row sum of the matching subset of rows of the normative connectome *C* corresponding to the voxels (or regions) affected by the set of lesions (Cm) for psychosis^37^ (**t**) and amnesia^48^ (**u**). In **f**–**u**, spatial spin permutation (Main and Methods) was used to assess statistical significance (*P*_spin_ < 0.001, two-sided, *n* = 10,000 permutations, *P* values shown in Supplementary Table 4).

We empirically tested the predicted systematic alignment of published LNM and sLNM maps to degree and other basic elementary properties (see below) of the normative functional connectome. We computed the row-summation vector of the group-average connectivity matrix of the GSP1000 dataset as used in Lead-DBS^55^. Then we correlated the resulting voxel-wise and atlas-based degree map with a series of reported LNM networks. Results support the prediction that LNM network maps strongly represent the summation vector of the normative connectome (multiple examples shown in Fig. 4). For example, LNM networks presented for addiction^38^ (three conditions, *r* = 0.81/0.70/0.82), neglect syndrome^53^ (*r* = 0.70, Fig. 4m), disrupted agency^16^ (*r* = 0.59, Fig. 4n), symptoms related to concussion^61^ (*r* = 0.50), emotional processing in depression^24^ (*r* = 0.74), schizophrenia^28^ (*r* = 0.97, Fig. 4p), bipolar disorder^28^ (*r* = 0.97) and OCD^28^ (*r* = 0.96) all show a strong association with the summation vector of *C* (*P*, *P*_spin_, *P*_brainsmash_ < 0.001; Supplementary Note 10; *r* and *P* values listed in Supplementary Table 4).

Similarly, published network maps derived by means of sLNM and related variants, for example, networks reported from addiction^38^ (Fig. 4j, *r* = 0.63), risk for depression in multiple sclerosis^34^ (Fig. 4i, *r* = 0.44), or networks hypothesized to reduce anxiety and depression symptoms^14^ (*r* = 0.56) showed a significant trace of degree (*P*, *P*_spin_, *P*_brainsmash_ < 0.001; Supplementary Table 4) and more specifically the first principal component of *C* (|*r*| = 0.77–0.89; see also Supplementary Note 9).

LNM networks derived from small, homogeneous and/or highly focal lesions can similarly exhibit strong traces of degree. Examples are found in the application of LNM derived on the basis of smaller lesion datasets (for example, aphasia^62^, *n* = 20, *r* = 0.74; migraine^20^, *n* = 11, *r* = 0.70; Fig. 4g,k; delusional misidentification^63^, *n* = 17, *r* = 0.65; Supplementary Table 4). The same holds for application of LNM and sLNM to DBS and TMS target sites^43,64^, where the stimulation sites are often highly localized within a radius of millimeters or centimeters (for example, TMS to DLPFC target sites to treat depression symptoms, *r* = 0.53, DBS related to cognitive decline in Parkinson’s disease, *r* = 0.64, *P*, *P*_spin_, *P*_brainsmash_ < 0.001; PC1 |*r*| = 0.84/0.92; Fig. 4r,s). Similarly, traces of degree are present when considering LNM maps derived from lesions with considerable spatial overlap (psychosis^37^, ~30% lesions in midbrain, |*r*| = 0.52; amnesia^48^, ~50% lesions in the thalamus, |*r*| = 0.12), but with these maps even more strongly reflecting the row-summation vector of their selected row graphs *Cm* (*r* = 0.95–0.98; Fig. 4t,u).

In total, of the 102 published LNM and sLNM networks we re-analyzed, 78 showed a significant trace of degree (*P*_spin_ < 0.05, 91 of 102 for *P*_brainsmash_ < 0.05; Supplementary Table 4). Below, we will further discuss the (non)specificity of these LNM maps, showing that the spatial patterns of almost all LNM networks can be explained by means of the same elementary properties of the standard matrix *C*.

### Elementary properties of whole-brain organization shape LNM outcomes

The fundamental properties of functional connectome organization—for example, modularity^58,65,66^, hubs^67^, anticorrelation^68^, gradient structure^69^—constrain LNM and sLNM maps to reflect the network membership of lesion sites. The linear nature of LNM (equations (3) and (4)) implies repeated sampling of one and the same fixed matrix *C*, leaving lesion projections *M*, and joint symptom projections *sv* in the case of sLNM, inherently constrained to the principal subspace defined by *C* (Supplementary Note 9). Distributed lesions yield LNM maps that approximate the global degree sequence (as in many reported LNM networks; Fig. 4). Conversely, clustered lesions cause the procedure to mirror the functional module or resting-state network(s) in which the lesions are located. Simulations confirm that >90% of generated FC lesion maps correlate with the canonical resting-state networks derived from modularity analysis of *C* (Methods), a pattern replicated in patient lesions and published circuits (*r* > 0.3; Supplementary Table 4). Moreover, the anticorrelated architecture of the connectome (that is, ~47% connections in the GSP1000 connectivity matrix are anticorrelated) ensures that LNM maps often show negative correlations with maps derived from lesions in opposing networks. Thus, patterns of anticorrelated LNM networks often interpreted as biologically meaningful in LNM studies^14,70,71^ are likely predictable consequences of the combined modular and anticorrelated structure of the standard connectome dataset.

### Specificity and explained variance of LNM networks

We examined the level of disease-specificity of LNM networks. On average, each LNM network showed a strong spatial overlap of |*r*| > 0.6 with 24 of the other 102 networks (*P*, *P*_spin_ < 0.05). This supports the very low—if not negligible—disease-specificity of LNM maps. This lack of specificity reflects the intrinsic nature of the LNM procedure. As we have seen above (equations (3) and (4)), LNM and sLNM repeatedly use the same low-rank matrix *C*, which limits the outcomes of the procedure to main patterns already present in *C*. To further illustrate this, we constructed a linear regression model that reflects nine basic factors describing the elementary properties of *C*—that is, its subcortical and cortical degree and its modular (*n* = 4) and functional gradient (*n* = 3) architecture^69^, core aspects of functional brain connectivity documented extensively in the field (for example, refs. ^58,65,66,69,72,73^; Methods). Regressing LNM maps of which lesion data was available against this simple model showed that 93% (mean, s.d. = 5.0%) of the variance in LNM networks is explained by the basic properties of *C* (Supplementary Note 14). We found similar findings for published sLNM-derived networks (*R*^2^ = 79%, s.d. = 10.2%; Supplementary Table 4). Any remaining variance falls well within the expected noise level of fMRI and LNM data^74,75^. These findings suggest that published disease LNM networks include no substantive information, other than unspecific signal already captured by global properties of functional connectome organization.

### Statistical procedures of LNM

LNM studies typically include statistical tests to support the sensitivity and specificity of presented networks^16,32,76^ (Supplementary Fig. 5). We briefly discuss the validity and meaning of these statistical tests in light of the above observations, with an extended discussion in Supplementary Note 15.

#### Sensitivity test

In the LNM approach, step 2 often involves the use of a one-sample *t* test to assess whether voxel-wise FC differs from zero. However, the large size of the normative dataset often leads to widespread significance^77^. For example, running 50 synthetic lesions in Lead-DBS shows that, on average, 64% voxels exceed the common |*t*| > 7 threshold. Moreover, this step contributes little additional statistical value. As shown above (Fig. 2c), the one-sample *t* test can be replaced altogether by simply taking the mean of the Fisher *r*-to-*z*-transformed correlations.

#### Specificity test

The specificity test evaluates the disease- or condition-specificity of the examined LNM map (Supplementary Fig. 5). This is typically conducted using a two-sample *t* test contrasting the derived map from a set of localized patient lesions with a set of random lesions drawn from other disorders^32,76^. Although this procedure appears to constitute an additional null test, it is largely redundant with the liberal sensitivity test. With LNM of random lesions to converge to the degree sequence of the normative matrix *C* (equation (3); Supplementary Fig. 2), the specificity test effectively re-assesses the same signal as the sensitivity test, but now relative to the matrix degree rather than zero. This highlights a lack of statistical independence between procedures intended to capture distinct information (for simulation analyses, see Supplementary Note 15).

#### Conjunction test

A commonly performed final test involves generating a conjunction or convergence map^22,37,76^, identifying voxels that pass both the sensitivity and specificity tests (Supplementary Fig. 5). Given the relative ease of passing the sensitivity test and its interdependence with specificity, such conjunctions are easily obtained. We modeled ~500,000 lesions across all brain regions (Yeo-Schaefer1000/Melbourne54) with varying levels of overlap using standard LNM settings (sensitivity |*t*| > 7, *G* = 75%; specificity |*t*| > 10 (for example, ref. ^57^); Supplementary Note 15). Marginal overlap between lesions (Dice = 0.08) resulted already in significant group results (10% sets), with minimum levels of overlap (Dice = 0.16) yielding 64% significant sets, increasing to almost all sets to reveal significant regions (97% tested sets) as spatial overlap increased (Dice > 0.25; Supplementary Fig. 6).

## Discussion

LNM has emerged as a widely used approach for identifying brain circuits linked to neurological and psychiatric conditions, as well as their symptoms^9,10,17^. Our analysis reveals a foundational limitation of the LNM framework—it maps circumscribed brain changes mostly to one and the same outcome, reflecting only elementary properties of the normative connectome. Given that these challenges arise from the LNM method rather than from extensive circuit-level findings in clinical neuroscience, this knowledge may aid the development of new network-mapping techniques.

Our findings have broad implications for a wide range of existing work regarding disease networks and circuits derived by means of LNM, used here as an umbrella term that unifies various related terminologies and methods in literature ((for example, the method is also commonly applied under labels such as “atrophy network mapping,” “activation network mapping,” or “network-based meta-analytic” analysis; Main and see for an overview Supplementary Table 1). The current results suggest that a substantial proportion of the presented LNM networks are nonspecific and may not accurately reflect genuine biological brain networks. In practice, the LNM captures only a small set of factors that describe broad features of the input connectivity matrix and has limited ability to identify subtle disorder-specific properties.

The convergence of LNM networks onto elemental properties of the connectome could be interpreted as support for the biological plausibility of LNM networks and circuits, like reflecting a transdiagnostic network underlying multiple disorders^78^. High-degree brain hubs^66,67,79–81^, for example, have been extensively theorized to have a central role in the pathophysiology of a wide range of disorders^82–85^. However, this interpretation in the context of LNM is misleading. The convergence of LNM methods and variants to the row sum of the used connectivity matrix (equation (3)) or, in more general terms, to the latent factors of *C* (Supplementary Notes 9,10 and 14), is purely a mathematical consequence of the procedure, not evidence of correspondence with the brain’s hub, resting-state modular network or otherwise complex wiring architecture. Accordingly, when the empirical connectivity matrix is replaced by a randomized counterpart *C*′, or by other structured nonbiological matrices, the LNM outcome remains the product of the latent properties that govern *C*′ (Supplementary Fig. 3).

LNM is increasingly proposed as a framework to guide therapeutic applications of TMS and DBS^14,25,37,39,40,50^, with case studies performed^86,87^ and protocols for larger randomized controlled trials based on LNM networks registered (see Supplementary Table 1 for review). However, many such proposed targets—for example, the anticorrelated frontopolar cortex for substance use disorder^38^, or peak voxels to refine DBS target sites for epilepsy^26,27^—seem to primarily reflect the mean signal of the standard connectivity data, rather than identifying disease-specific loci. Indeed, the same regions emerge when LNM is applied across unrelated conditions^11,25,27,44,48,59^ (Supplementary Fig. 7) or just summing FC across all voxels in the GSP1000 dataset (Supplementary Fig. 8). Given the clinical impact of these procedures, it appears essential to thoroughly reassess these targets before substituting traditional stimulation sites with demonstrated efficacy^88–90^.

LNM studies have often been motivated by the observation that brain alterations in neuropsychiatric and neurological disorders are spatially diverse and heterogeneous—indeed, 55% studies describe them as such (Supplementary Table 1)^22–24,27–29,91–93^. This heterogeneity is frequently cited as a rationale for performing the LNM analysis, in search of an underlying common functional network that unites these brain alterations. Our findings propose a re-appreciation of disease heterogeneity, further studying how brain disorders may involve spatially distributed, heterogeneous alterations that converge on shared phenotypes^6,94^.

A remaining question is whether the methodological limitations of LNM can be alleviated through refinements of its statistical procedures, for example by using random(ized) lesions or seed locations as a null-model. We approach such a solution with caution. The observation that almost all meaningful variance in LNM maps is explained by basic properties of the connectivity matrix suggests that deviations from a reference model or baseline are likely minimal, if they exist. Indeed, 70 of 78 LNM maps where lesion data were available failed to reach even a liberal significance criterion set by a generative null-model based on random synthetic lesions (nominal two-sided *α* = 0.05, uncorrected for 78,000 tests; Supplementary Note 16). A similar outcome was observed from a permutation-based null model in which lesion locations were randomly shuffled while preserving modular prevalence (71/78, *P*_FDR_ > 0.05; Supplementary Note 16). We are also hesitant to propose a null-model solution for the LNM framework from a conceptual standpoint. Permutation-based null models estimate effects under random conditions by randomizing the input data. In LNM, only two variables exist at its core—*M* and *C*. With *C* describing the connectivity and remaining fixed in LNM studies and approaches^8,9,17,24^, the set of lesions (*M*) is left to be permuted. As predicted from equation (3), LNM on random sets of lesions consistently produces similar solutions dominated by degree (Supplementary Fig. 2). This inherent limitation of the LNM framework hinders the construction of a null distribution that fulfils the essential criterion of spanning a meaningful range of alternative maps for a valid null test.

The framework of network mapping has profoundly contributed to modern concepts of psychiatric and neurological disorders as network-based conditions^83,95–98^. LNM^8,9,23,99^ is proposed as a powerful and promising method within this framework to gain deeper insight into the mechanistic role of brain circuits in disorders^9–11^. Regrettably, our findings indicate that a substantial proportion of networks and disease circuits derived from LNM may not accurately reflect genuine disease-specific biological brain networks. However, it is crucial to separate the ‘theory’ from the ‘method’. While we, and experts in the field with whom we discussed our findings, were not able to find an enduring solution to the foundational methodological issues of LNM, a continuous community effort to study the role of brain circuits in neurological and psychiatric disorders is imperative for advancing our understanding and developing new, effective treatments for these conditions. Linking deviations in brain organization to behavioral outcomes has long served as a cornerstone of this effort—from early clinical observations^1^ to systematic studies leveraging modern neuroimaging techniques^4^. Embedded in efforts collectively referred to as disease connectomics^82,83,100^, proposed fruitful future directions for the field may lie in revisiting the original rationale of lesion and ‘voxel-based lesion–symptom mapping’ in context of connectivity mapping techniques^1–3,101–103^ to systematically chart how lesions impact brain circuitry and behavior. In parallel, network neuroscience^72,79,104^ offers the framework to more broadly investigate the central role of core network nodes in brain function and dysfunction^79,82–84^. Future efforts could focus in combining real patient lesions with in silico simulations to identify brain areas that may serve as general targets for intervention^105,106^. Such community efforts in revising the field of LNM from first principles may help ongoing work to develop brain network methods to map, understand and ultimately translate network-level approaches into clinical applications.

## Methods

### Published LNM maps, lesion datasets and LNM application

A systematic literature search (December 2025) on LNM studies was performed. This identified 201 LNM studies, including 187 LNM data studies, 9 reviews and 5 commentaries with LNM, sLNM and/or other related variants the focus of the study, published between 2015 and 2025 (see Supplementary Note 1 and Supplementary Table 1 for details). From these articles, we extracted data on 102 published (s)LNM networks across 72 studies on 50 neurological, 18 psychiatric and 4 behavioral conditions (creativity, political, healthy, facial emotion), including 18 downloaded LNM maps, 11 datasets with reported lesion prevalence, 350 original lesion masks, 935 lesions manually segmented from original papers and 8 coordinate-based LNM (*n* = 1,442 brain coordinates). Details about the data extracted from these studies are presented in Supplementary Note 2 and Supplementary Table 2.

### LNM

Voxel-wise LNM was performed using the Lead-DBS toolbox^54^ (settings, FullSet of GSP1000 participants^55^; Supplementary Note 3 and Fig. 2a). Equivalently, atlas-based LNM involved mapping lesions to the 1,000 cortical regions of the Yeo-Schaefer1000 atlas^107^ and the Melbourne54 subcortical atlas^108^ and selecting the matching rows of the selected parcels from the group connectome matrix *C* (Fig. 2c).

### Comparison of LNM maps

Spatial overlap of LNM maps was computed using Pearson correlation coefficients, using voxel-wise correlation for available voxel-wise maps and atlas-space for atlas-based maps. Significance was further assessed using the spin-null model^45^ and the BrainSMASH generative null model^46^ (10,000 permutations) to account for spatial autocorrelation effects. For 102 downloaded and reconstructed LNM maps (see Supplementary Table 2 for sources), the top 10% correlated and anticorrelated voxels were binarized and averaged to generate an overlap map of LNM regions across published studies.

### Normative connectome

A group functional connectome matrix *C* was formed by mapping the same functional time series of the GSP1000 participant data as used in voxel-wise LNM^54,55^ (Supplementary Note 4) to the Yeo-Schaefer1000/Melbourne54 atlas and averaging the computed individual FC matrices into the group matrix (no thresholding).

### Network analysis

The summation vector of the group connectivity matrix *C*, or degree, was calculated as the row sum of the connectivity matrix *C* using the Brain Connectivity Toolbox^110^. Functional modules^66^, reflecting the composition of resting-state networks^58,73^, were identified using the Newman modularity algorithm^110^.

### Regression model

A simple model describing the elementary factors of the connectome matrix was formed on the basis of the derived network metrics. Subcortical, whole-brain and modular degree were computed as the mean connectivity of matching regions from the brain atlas, together with three FC gradients^69^ taken as the first three components of a principal component analysis on *C* (Supplementary Note 13).

### Spatially randomized lesions

Randomized lesions were generated by several randomization strategies, including spatially rotating the original cortical lesions across the cortical surface (spin-null permutation^45^). Alternative randomization methods included the generation of random synthetic lesions by taking random samples from the brain atlas (matching lesion size) and a biologically driven randomization that drew random lesions from the total collection of clinically informed lesions associated with a wide range of conditions and disorders (Supplementary Note 6).

### Randomized connectivity

A randomized normative connectome matrix was generated by randomizing the connections in the connectivity matrix *C* (threshold *r* > 0.2, other thresholds yielded similar results) using the rewiring method described in refs. ^51,52^. LNM maps were compared between the original connectome and its randomized counterparts (1,000 permutations performed; see Supplementary Note 7 for details). Alternatively, full degree-disrupting randomization of *C* was examined.

### Simulations

Synthetic lesions were constructed by randomly selecting regions with equal probability *P* = 1/*R* from all regions in the atlas, dilated by including the closest neighboring parcels (*n* = 4), and for voxel-wise LNM further mapped to corresponding voxels in the MNI atlas volume. Lesion sets (*n* = 50) with varying levels of overlap between lesions were created by randomly selecting a first lesion with probability *P* = 1/*R* from all brain regions, with the second to *n*th lesion placed in that region as 1/*R* × *q*, and in all other brain regions with probability *P* ~ 1/*R*. As such, parameter *q* ensured a variable level of overlap between some of the lesions in the set, while all other lesions remained completely randomly distributed across the brain. The level of lesion overlap within each set as a function of *q* was quantified by means of the average Dice coefficient among all lesion pairs in the set, ranging from zero (no overlap) to one (complete overlap; see Supplementary Note 16 for details). We refer to Supplementary Note 16 for null-model simulations using synthetic lesions and randomizing patient lesions, preserving modular assignment.

### Reporting summary

Further information on research design is available in the Nature Portfolio Reporting Summary linked to this article.

## Online content

Any methods, additional references, Nature Portfolio reporting summaries, source data, extended data, supplementary information, acknowledgements, peer review information; details of author contributions and competing interests; and statements of data and code availability are available at 10.1038/s41593-025-02196-7.

## Supplementary information

Supplementary Information Supplementary Notes 1–20, Supplementary Figs. 1–8 and Supplementary Tables 1–4.

Reporting Summary

## Data Availability

All data used in the present study are publicly available. The preprocessed normative FC time series from the GSP1000 dataset are available at 10.7910/DVN/ILXIKS and in the Lead-DBS toolbox^54^. Neuroimaging data from the Human Connectome Project are available at https://www.humanconnectome.org. All LNM maps used in this study are available at https://neurovault.org and on GitHub (https://github.com/dutchconnectomelab/lesionnetworkmapping). Lesion masks associated with amnesia, hypersomnia, insomnia, neglect syndrome and Alice in Wonderland syndrome are available at https://www.lesionbank.org/. All other reported lesion or LNM data are directly available from the referenced papers.

## Code availability

Voxel-wise LNM was applied using the open-source Lead-DBS toolbox^54^ (https://www.lead-dbs.org/). Spin-null permutation was conducted using the BrainSpace toolbox^111^. Network analysis of the normative connectome was performed using the Brain Connectivity Toolbox^110^. Steps of the streamlined LNM procedure, mathematical derivation, simplification of the method and example code are presented in Supplementary Notes 17–20 and at https://github.com/dutchconnectomelab/lesionnetworkmapping.
