# Supplementary material for: Investigating the methodological foundation of lesion network mapping
Source: Nat Neurosci. 2026 Jan 15;29(5):1237–47. doi: 10.1038/s41593-025-02196-7 (PMC13156034; doi:10.1038/s41593-025-02196-7)
Supplement: Supplementary file 1 — Supplementary Notes 1–20, Supplementary Figs. 1–8 and Supplementary Tables 1–4. [file 41593_2025_2196_MOESM1_ESM.pdf]

nature neuroscience

Supplementary information https://doi.org/10.1038/s41593-025-02196-7

Investigating the methodological foundation of lesion network mapping

In the format provided by the authors and unedited

Supplementary Notes of

Investigating the methodological foundation of Lesion Network Mapping

Authors
Martijn P. van den Heuvel, Ilan Libedinsky, Sebastian Quiroz Monnens, Jonathan Repple, Iris Sommer, Luca Cocchi

Corresponding author: Martijn P. van den Heuvel
E-mail: martijn.vanden.heuvel@vu.nl

S1. Systematic review of LNM papers, grants, clinical trials
S2. Data resources
S3. LNM tools and Lead-DBS
S4. Normative functional connectivity data
S5. LNM analysis and comparison of published LNM maps
S6. Comparison of LNM maps from real and randomized lesion locations
S7. LNM analysis on randomized functional connectome matrix and synthetic matrices
S8. Step-by-step analysis of LNM procedure and formal notation
S9. LNM methodological variants
S10. Spatial convergence of LNM networks to degree of *C*
S11. Lesion overlap and LNM similarity within and between conditions
S12. Similar networks systematically result running replication analyses
S13. Regression model
S14. LNM (non) specificity
S15. Extended discussion on LNM statistical procedures
S16. Null-models
Supplementary Figures 1-8
S17. Pseudocode for Lesion Network Mapping
S18. Mathematical derivation of LNM
S19. Mathematical derivation of sLNM variant
S20. Code examples
References
Supplementary Tables 1-4

S1. Systematic review of LNM papers, grants, clinical trials

A systematic review of LNM literature (December 2025) was conducted to identify neuroimaging studies that implemented lesion network mapping (LNM). The PubMed search included the terms: “lesion network mapping”, “causal brain mapping”, “causal network localization”, “lesion network localization”, “coordinate network mapping”, “causal brain circuit”, “lesion-derived network”, “connectome-based lesion mapping”, “lesion network map”, “causal circuits”, and “mapping lesion” (all searched in the Title/Abstract field). Each paper was reviewed to confirm the involvement of LNM, and results were supplemented by manual searches in PubMed and Google Scholar. Study eligibility was determined using a two-stage screening procedure. First, for all candidate studies, the Methods section and Supplementary information and materials were systematically reviewed to confirm the involvement of (i) a normative connectivity dataset; (ii) network-based mapping of focal brain perturbations using that normative dataset; and (iii) aggregation of the resulting normative connectivity maps, derived from brain lesions, stimulation targets, reported coordinates, regional atrophy, or related perturbations. Second, when available,

accompanying code repositories were inspected to verify implementation details. For studies without publicly available code, inclusion decisions were based solely on the Methods section. This procedure identified 201 studies (including 9 review articles and 5 commentaries) published between 2015 and 2025 that discussed, applied or otherwise involved LNM and/or proposed variants of the method. These studies described 101 different conditions and study designs, including neuropsychiatric, neurological, and neurodegenerative disorders, as well as cognitive brain functions. Supplementary Table 1 provides a list of the identified 201 LNM studies that discussed or used LNM. We were able to extract data from 72 of these studies (Supplementary Table 2), including lesion and/or LNM, sLNM or group-contrast sLNM data (see Supplementary Note S9 for variants) for analysis. See Supplementary Note S2 for data resources and data extraction and Supplementary Note S3 for (s)LNM analysis.

The NIH RePORTER database (https://reporter.nih.gov/) was used to identify grants related to LNM applications. Fourteen NIH-funded projects were found that applied LNM to identify disorder-related brain circuits, support diagnostic efforts, or guide stimulation targets for interventions such as transcranial magnetic stimulation (TMS), transcranial electrical stimulation (tES), and deep brain stimulation (DBS). These grants spanned a range of neurological and psychiatric conditions, including depression, anxiety, stroke, psychosis, schizophrenia, autism spectrum disorder, epilepsy, addiction, obsessive-compulsive disorder, Parkinson's disease, and Alzheimer's disease.

Ten clinical trials utilizing LNM were identified through ClinicalTrials.gov, including eight ongoing and two completed. Of these trials, nine were interventional, including seven with a double-blind design, and one was observational (retrospective). These trials address a range of conditions such as bipolar mania, depression, anxiety, obsessive-compulsive disorder, psychosis, Parkinson's disease, and stroke. The primary aim is to use LNM to guide stimulation targets for interventions (TMS, tES, DBS).

S2. Data resources

In total, 102 (99 unique) LNM maps based on extracted lesion set data, lesion prevalence data, neuroimaging abnormalities coordinates data, and LNM maps of 72 published LNM studies were extracted (listed in Supplementary Table 2).

Published LNM maps. A total of 18 published LNM maps were obtained directly from the respective papers, for, among others, conditions of depression [1], post-traumatic stress disorder [2], anxiety-depression symptoms [3], psychosis [4], anosognosia [5], impaired verbal memory in multiple sclerosis [6], depression-related symptoms in multiple sclerosis [7], epilepsy [8], substance abuse disorder [9], aphasia [10], political involvement [11], transdiagnostic psychiatric illness [12], depression remission [13], as well as DBS-derived or related networks for obsessive-compulsive disorder [14], cognitive decline in Parkinson's disease [15], tremor relief (probabilistic map of symptom severity)[16], Alzheimer's disease [17], dystonia [18](see for resources Supplementary Table 2). To facilitate direct comparison across studies, maps with arbitrary sign direction [3, 5, 15] were inverted. The similarity of these LNM maps was quantified by calculating Pearson's correlation coefficient at the voxel level. For atlas-based LNM applications and correlations, LNM maps were mapped to the Yeo-Schaefer1000 brain atlas.
Lesion masks. 129 published MNI masks for lesions associated with nicotine smoking addiction [19], 48 with disrupted agency [20], and 28 with disrupted volition [21], along with 145 additional case reports related to amnesia, hypersomnia, insomnia, neglect syndrome, and Alice in Wonderland syndrome [22] were downloaded from their respective data resources (Supplementary Table 2).

Lesion segmentation. For resources of which the lesion masks were not directly available, but lesions were presented in the papers, lesion masks were segmented from the presented lesion figures and/or from the original documented case reports (studies listed in Supplementary Table 2). Lesions were segmented in the Montreal Neurological Institute (MNI) brain template using the FMRIB Software Library (FSL) [23]. In total, over 900 lesions were segmented across 37 conditions from 36 studies.

Published prevalence scores. Published disorder-related LNM maps and prevalence data on cortical deviations for schizophrenia, major depressive disorder, attention-deficit/hyperactivity disorder, bipolar disorder, autism spectrum disorder, obsessive-compulsive disorder, and healthy controls [24] were obtained across 1,000 cortical regions based on the Yeo-Schaefer1000 brain atlas [25]. For other studies of which lesion prevalence maps were reported (Supplementary Table 2), regional prevalence scores were extracted and lesions were generated by sampling brain regions based on the prevalence scores and dilating each lesion by a standardized 5 neighboring regions. In case where lesion prevalence data were not directly available, but were presented in the figure(s) of the papers, the presented MNI slices with prevalence values were sampled from the pdf file, matched to MNI space, and regional prevalence scores were extracted in Melbourne54 [26] and Yeo-Schaefer1000 atlas space.

Coordinates data. For resources of which MNI voxels of cortical and subcortical lesions/deviations were reported (Supplementary Table 2), MNI coordinates were extracted from the main report and/or the documented individual papers. Standardized volumetric masks were created by dilating each point by 5 voxels in all directions, following the original steps of the LNM papers (e.g.,[8, 27-33]).

S3. LNM tools and Lead-DBS

Voxel-wise LNM was performed using Lead-DBS [34] using Lead-DBS v3.1 (source: https://www.lead-dbs.org/download-lead-dbs/) and MATLAB R2024b, along with the SPM dependency (e.g., source: https://www.fil.ion.ucl.ac.uk/spm/software/spm12/). No modifications to the source code of Lead-DBS were made. The installation instructions available in the Lead-DBS user guide (https://netstim.gitbook.io/leaddbs) were used (section: data description) to download and install the *GSP1000 Preprocessed Connectome for Lead DBS* (Version 3.0) normative connectome was downloaded from the Harvard Dataverse (source: https://doi.org/10.7910/DVN/KKTJQC). LNM was conducted using the Lead-DBS lead_mapper GUI and by using the function *cs_fmri_conseed_seed_tc.m.* Lesions were run through Lead-DBS in the MNI152 coordinate system.

S4. Normative functional connectivity data

GSP1000 connectome dataset. The normative connectome data of the GSP1000 dataset [35] was used as the default of the Lead-DBS toolbox. The GSP1000 connectome dataset contains voxel-wise pre-processed functional time-series of 1000+ subjects, used for computation of lesion individual and group-based LNM functional connectivity maps. A group-based GSP1000 connectome matrix was made by mapping all individual voxel-wise time-series to atlas space, computation of a region-to-region connectivity matrix for each individual dataset by correlating region-wise time-series, and followed by averaging the obtained individual connectivity matrices into one average group connectome matrix *C.*

HCP connectome dataset. As an alternative normative functional connectivity dataset, field-standard data from the Human Connectome Project was used, similarly to the GSP1000 dataset containing high-quality functional connectivity data of a large dataset of healthy control young adult [36]. Pre-processed T1, diffusion MRI and minimal preprocessed functional

time-series were downloaded from the HCP portal, mapped to the anatomical atlas, and processed into individual functional connectivity matrices [37], including band-pass filtering (0.01-0.1 Hz), motion scrubbing [38], after which a single group connectivity matrix C was computed by averaging all individual functional connectivity matrices.
1000 functional connectivity data. As a further alternative, functional connectivity data of the 1000 functional connectome project [39] was used (source: www.nitrc.org/projects/fcon_1000/) and processed using the same pipeline as the HCP connectome data (see previous paragraph).

S5. LNM analysis and comparison of published LNM maps
In total, LNM data from 72 studies reporting on 102 LNM circuits were reconstructed, including originally published voxel-wise LNM maps, lesion locations and/or masks, and lesion prevalence data across patient cohorts (see Supplementary Note S2 and Supplementary Table 2 for resources). For those studies of which voxel-wise LNM sensitivity maps were publicly available, we directly downloaded the maps and only normalized them to a standard normal distribution (mean = 0, SD = 1) for cross-study comparison. For studies providing original lesion masks, lesions were downloaded and processed using Lead-DBS to generate LNM maps by averaging lesion t-maps (or equivalently r-maps). When lesion masks from a study were unavailable but lesion locations were provided in figures, supplementary materials, or original case reports, lesions were manually segmented onto the standardized MNI152 template and analyzed using Lead-DBS. For studies where only lesion prevalence data was made available, 100 synthetic lesions (each comprising five parcels) were generated based on the prevalence maps, which were then similarly analyzed with Lead-DBS. For each of the lesion sets, a LNM sensitivity map was created using voxel-wise Lead-DBS and atlas-based LNM. For the latter analysis, maps were projected onto the cortical Yeo-Schaefer1000 [25] and subcortical Melbourne54 atlases [26]. Spatial overlap between the published LNM circuits was quantified by calculating Pearson correlation coefficients between pairs of LNM maps, in voxel-wise space for available voxel-wise LNM maps and atlas-space for LNM maps generated using atlas-based LNM. To account for spatial autocorrelation in the LNM maps, the statistical significance of correlations was further assessed by using spin-null model [40] (10,000 permutations, two-sided) and by means of the BrainSMASH generative null-model [41] (10,000 permutations, two-sided). Comparison across all considered 102 LNM maps (99 unique) revealed a high spatial overlap. Across maps, we observed an average spatial correlation of $|r| = 0.40$ (SD = 0.25) between voxel-wise Lead-DBS maps. An $|r|$ = 0.43 (SD = 0.26) was observed between maps at atlas-based resolution.

LNM overlap map. An LNM overlap map was generated across the LNM maps, binarizing the top 10% of most correlated and most anti-correlated voxels and averaging across published studies. Several brain regions were found repeatedly reported across a large number of published LNM maps, with the peak voxel overlap in the insula (left/right hemisphere = 74/74% of LNM maps) and adjacent pars opercularis (73/73%), precentral (74/72%) and postcentral gyrus (64/60%), caudal anterior cingulate gyrus (cACC, 67/71%) and posterior cingulate cortex (63/68%), putamen (63/71%), anterior PFC/frontal pole (BA10, 52/55%), and cerebellum (62/65%), indicating limited disease specificity.

S6. Comparison of LNM maps from real and randomized lesion locations
The effect of randomizing lesion locations on the resulting LNM map was examined as follows. First, per condition, lesions were mapped to the Yeo-Schaefer1000 atlas [25]. Atlas-based LNM (Figure 2C) was used to compute the LNM map of the original lesions. Second, random lesions were generated (1,000 times) and the LNM maps from the real and random

lesions were compared using Pearson's correlation. Three different methods of randomization were assessed:

Spatial randomization. For each lesion, a random location in the Yeo-Schaefer1000 atlas was selected, and a simulated lesion of the same size was created by including the selected region and its closest neighboring regions matching the size of each lesion.

Spin-model randomization. Alternatively, the spin-null model [40] was used to rotate the original lesion across the cortical surface keeping the original number of regions with lesions. This null-model was implemented with the BrainSpace toolbox [42].

Biologically-informed null-model: random mix of patient lesions. As a third condition, LNM maps were computed based on equally sized sets of lesions (n = 100) randomly drawn across the total set of segmented lesions across all conditions (n = 1,285 across 47 conditions, Supplementary Table 2). This null-model provides a real-world comparison that accounts for clinically-informed prevalence of lesion locations across the brain.

Complete randomization. Finally, a fourth null condition was tested in which a number of Yeo-Schaefer1000 regions, equal to the number of regions in the original lesion, was randomly selected across the cortex. Unlike real lesions, these simulated lesions were spatially scattered, with selected regions not necessarily neighboring each other, in contrast to the more anatomically plausible distributions used in the other two randomization approaches.

S7. LNM analysis on randomized functional connectome matrix and synthetic matrices

Atlas-based LNM analysis (Figure 2C) was performed by parcellating both the brain lesions and the normative connectome into 1,000 distinct regions using the Yeo-Schaefer1000 atlas [25]. For the normative connectome, the group average functional connectivity matrix derived from GSP1000 [35] was used, thresholding connections at $r > 0.2$.

Degree-preserving randomization. Degree-preserving randomized connectomes were generated using degree-preserved Maslov and Sneppen (MS) randomization [43] of the normative connectome, with 100 random swaps of connections. Running LNM on the original lesions using the randomized matrix C resulted in highly similar maps as compared to running the LNM using the human functional connectome C. Results and examples of these are shown in the main text and in Figure 1. Equation 3 provides a formal explanation for this observation of obtaining the same LNM map when a randomized normative connectome is used (Figure 1T-V, Supplementary Figure 3). The MS randomization randomizes the edges of the network, but preserves the total degree distribution of the matrix. With the LNM map converging to the sum of the matrix, LNM using a MS randomized matrix will again converge to the same LNM map.

Degree-disrupting randomization. An additional condition was analyzed in which the normative data underwent degree-disrupting randomization, specifically through randomization of connectivity in the normative connectome C'. As predicted, only when the degree sequence of nodes was fully disrupted and no other clear structure remained in C', the convergence of LNM to overall degree was disrupted, leaving random LNM outcomes.

Synthetic connectivity matrices C. The behavior of LNM was evaluated using randomly generated synthetic connectivity matrices C', having no reflection of biologically-informed connectivity. The generative models used to construct C' included synthetic Barabási–Albert scale-free networks [44], synthetic block networks with four distinct blocks [45], and synthetic star-shaped networks (Supplementary Figure 3). LNM was used again on the same lesion

sets, with the only difference of changing *C* to *C′*. As predicted from Equation 3 (main text), LNM maps resulting from using these alternative matrices *C′* again resulted in highly consistent maps. In all cases, the LNM procedure converged to basic properties of the used connectome matrix, here the alternative connectivity matrix *C′*. Results of exemplary cases are shown in Supplementary Figure 3.

S8. Step-by-step analysis of LNM procedure and formal notation
Figure 2A presents a schematic version of the three core steps of the LNM procedure, as implemented in popular LNM toolboxes such as Lead-DBS [34] and used in many LNM studies. First, (**Step 1**) lesions or brain deviations [24, 46] are aligned to a standard brain template. Next, (**Step 2**) the standardized lesions are mapped onto functional brain networks by propagating the lesions through the functional brain connections of a large normative functional connectome dataset, after which (**Step 3**) the resulting functional connectivity maps are combined to reveal an underlying shared LNM circuit [47]. See Supplementary Note S17 for pseudocode describing the LNM procedure.

Voxel-wise lead-DBS implementation. More formally, we can consider a group of patients *S,* with each patient *s* having one (or more) lesion(s) *m*. We further have a large normative connectome dataset *H* representing all the resting-state fMRI time-series of the large normative group in a standard space. In many LNM studies, standardized fMRI data of 1,000+ healthy control subjects from the GSP1000 dataset [35] or from the Human Connectome Project [36] are used as a high quality normative dataset. For a lesion *m* of individual patient *s,* **Step 1** involves projecting lesion *m* onto the corresponding set of voxels *i* in the standardized space of the normative dataset *H*. Next, in **Step 2** the level of functional connectivity of the time-series of voxels *i* to all other voxels in the brain is computed to find the functional connections of the regions corresponding to lesion *m*. This process is repeated for all datasets in the normative dataset *H.* In the Lead-DBS toolbox for example, this process is repeated for all 1000+ normative subjects in the GSP1000 dataset, after which all of the correlation *r*-maps are standardized by applying a Fisher *r*-to-*z* transformation [48]. The resulting 1,000+ normative FC maps are combined by applying a one-sample *t*-test*,* testing for each voxel in the brain how strong the functional connectivity deviates from zero across the total group *H*. An optional follow-up step is thresholding the resulting *t*-map with a threshold $|t|$ to identify all significant functional connections of the lesion at hand (Figure 2A). Often a stringent *t*-threshold of 7 or higher is used to account for the large number of voxel-wise tests performed [49]. **Step 1** and **2** are repeated for all lesions across all the patients in the dataset, resulting in a (optionally thresholded) functional connectivity whole-brain connectivity map for each lesion *m* of patient *s*.

In the group analysis **Step 3**, all these lesion *t*-maps are joined into a final LNM brain map by selecting all functionally connected areas that are consistently found across the total set of lesion maps. Often a group threshold of 75% or higher is used to mark only the highest consistent regions in the proposed LNM network [46]. The resulting final LNM sensitivity map is presented as a map that unites the diverse set of lesions onto a common underlying brain circuit.

Accelerating the steps. We note that this procedure can be accelerated by streamlining several intermediate steps in Step 2 (formal derivation provided in Supplementary Note S18), without losing any information ($r = 0.99$ empirical equivalence between both implementations). First, rather than using a voxel-wise approach [34], one could adopt an atlas-based approach by applying a high-resolution brain atlas, such as the Yeo-Schaefer1000 atlas [25] to both the lesion locations and the resting-state fMRI time-series [24]. This has several advantages. Functional connectivity between only e.g., a 1,000 regions instead of 200,000+

voxels have to be considered, which allows for the pre-computation of all possible R x R lesion-to-region functional connectivity values for all datasets in H beforehand. A Fisher r-to-z transformation can be optionally applied to each of the individual matrices. With the majority of FC values to lie within the [-0.5 to 0.5] range the transformation is mostly linear, and leaving out this step will lead to similar results (see below for validation) for the group matrix C (we verified this step, see below). The resulting pre-computed functional connectivity matrices can then be directly substituted in Step 2 (Figure 2B), and the commonly used one-sample t-test [34] in Step 2 across all normative subjects in H can be replaced by taking the mean of the pre-computed matrices, when we approximate the variance by equal variance across the edges of the matrix, scaled now by only a fixed constant sqrt(H)/std(FC). We verified the validity of this assumption, showing that using the voxel-wise t-maps output of Lead-DBS or taking the mean over the raw correlation r-maps results in virtually identical results (Lead-DBS, mean $r = 0.99$). Using the mean of the raw r-maps allows the 1,000+ individual connectome maps of the normative dataset H to be replaced by a single group-averaged functional connectivity matrix C, which can then be used uniformly in Step 2. This eliminates the need to repeat the procedure for all 1,000 individuals in H for each lesion (Figure 2B), without any loss of information. We also verified that the Fisher r-to-z step could be approximated as a linear step (see Supplementary Note S18), by performing first the Fisher transformation on each of the 1000+ individual matrices, computing the mean over the matrices and performing the inverse transformation to obtain a new group matrix C. The two group matrices (i.e. with and without the Fisher r-to-z transformation) were virtually identical ($r = 0.99$).

This streamlining of the LNM procedure reduces the computation time for a standard dataset of 50 lesions from ~12 hours using the Lead-DBS toolbox [34] to under 10 seconds using the streamlined implementation, while yielding virtually identical results. We mathematically (Supplementary Note S18) and empirically validated the equivalence of this streamlined approach and the full voxel-wise LNM implementation of the Lead-DBS toolbox [34], comparing both methods across 500 real lesions [22] and 500 simulated lesions. The resulting lesion wise LNM maps were virtually identical (mean $r = 0.96$, p, p_{spin}, $p_{brainsmash} < 0.001$).

Empirical validation of voxel-wise LNM and streamlined atlas-based LNM. Lead-DBS voxel-wise LNM and atlas-based LNM results were compared. Analyses were conducted using 100 lesions randomly selected from the total set of 1,285 lesions and 100 simulated lesions. For each simulated lesion, a region was randomly selected from the Yeo-Schaefer1000 atlas [25]. Each lesion was first run through the Lead-DBS toolbox (Figure 2A, main text), with default settings, computing an average resting-state time-series across all voxels within each lesion mask, correlating this time-series with all other voxels in the brain using the GSP1000 normative connectome, followed by Fisher r-to-z transformation and a one-sample t-test to assess whether the resulting functional connectivity values significantly deviated from zero across the GSP1000 subjects. For the streamlined atlas-based LNM (Figure 2C), lesion masks were mapped to the Yeo-Schaefer1000 atlas, and the LNM map was computed by means of the linear matrix form $M \times C$ (Equation 3). Across the 100 random clinically-informed lesions and 100 synthetic lesions, the two resulting outcome LNM maps were compared, resulting in virtually identical maps (real lesions: mean $r = 0.96$; simulated lesions: mean $r = 0.97$).

Validation of similar variation of signal across connections. The one-sample t-test performed in Step 3 can be approximated by computing the mean of the connectivity values, with equal variance across edges, as equal variance across edges makes the denominator of the t-test a fixed constant. This approximation was empirically validated by comparing the obtained t-

maps with maps obtained by estimating the mean over the functional connectivity correlation coefficients across subjects. Virtually identical maps were observed in both simulated and real lesion datasets (100 simulated lesions: mean $r = 0.99$; 100 real lesions: mean $r = 0.99$). Equally, performing a one-sample t-test across all Fisher r-to-z GSP1000 matrices yielded t-scores virtually identical to those obtained by averaging the connectivity matrices into the group matrix C, scaled with a fixed constant ($r = 0.99$).

S9. LNM methodological variants

In the main text we describe step-by-step the most applied form of the LNM methodology used in literature (see Supplementary Table 1 for literature review). Methodological variants of the LNM methodology have been proposed, including *symptom-based LNM* (sLNM), a variant that takes further into account individual patient symptom scores to determine the LNM map (~16% of articles) and a related group-contrast *symptom LNM* that specifically contrast dichotomous sub-groups of patients (for example contrasting patients with high and low symptoms, ~11% of articles). Below we describe in more detail the sLNM variants, and show by means of methodological reduction and empirical analysis of published sLNM maps that they similarly lead to artifactual results.

Symptom-based LNM (sLNM). Lesion network-symptom-mapping [50, 51] or 'symptom-based lesion network mapping', ' (sLNM) is a modification of the standard LNM procedure designed to incorporate information about individual symptom scores into the construction of the group LNM map. In the sLNM procedure, the first two steps mirror those of standard LNM. Step 1 again involves transforming the individual patient lesions into standard space, and Step 2 uses the normative functional connectome (e.g., +1,000 individuals from the GSP1000) to compute individual lesion functional connectivity maps.

In the main text and Supplementary Note S18, we have seen that Step 2 can be described by selecting the lesion-matching rows Cm from the group normative matrix C, which can further be described as a matrix multiplication $M \times C$, with M the joint matrix of all $\{ \vec{m}_1, \vec{m}_2, ..., \vec{m}_S \}$ lesion vectors $\vec{m}_s$ of the patients S, and C the used connectivity matrix.

In the standard approach, in Step 3, the final LNM map is produced by taking the row-wise sum of the resulting matrix, summarizing the entire procedure to $LNM \propto \Sigma\ (M \times C)$. (Equation 3 main text, Supplementary Note S18). In the sLNM variant, Step 3 is modified (Supplementary Figure 1). The same row matrix Cm is selected (and thus can similarly be streamlined by $M \times C$, Equation 3), but the region-wise connectivity values in Cm are now correlated column-wise with a symptom vector sv, which contains a clinical score for each patient. The direction of sv depends on the scale used and can include various types of patient information. Examples used in sLNM studies include higher or lower risk of post-traumatic stress disorder [2], networks hypothesized to reduce anxiety and depression symptoms with neurostimulation [3], symptom improvement or worsening of depression [1], or improvement in treatment-related symptoms [52, 53]. Correlating the clinical variable sv with each column of Cm (that is, each voxel in R), produces the final sLNM r-map of size $1 \times R$, containing an r value for each voxel or region.

The sLNM procedure can also be approximated as a linear expression between the symptom scores sv, the lesion matrix M and the connectivity matrix C. With the correlation coefficient r between two vectors x and y given by the standard equation

$$r = \frac{\sum_{i=1}^{n}(x_i - \bar{x})(y_i - \bar{y})}{\sqrt{\sum_{i=1}^{n}(x_i - \bar{x})^2}\sqrt{\sum_{i=1}^{n}(y_i - \bar{y})^2}}$$

, the correlation between sv when standardized (mean = 0, SD = 1) and the functional connectivity across the set of lesions to a specific region j in the atlas R (= one column r in the Cm matrix) can be derived as $r_j \propto \sum_{i=1}^{S} Cm_{i,j} \times sv_i$, given a constant $k = 1./((S\text{-}1) \text{ x std}(Cm) \text{ x } 1)$, with S the number of patients/lesions. *Note:* lesion m_i of subject i

matching row i of C is now used to indicate the lesions and thus the matching rows taken from matrix C, and index j is now further used to indicate the columns of each of rows in Cm corresponding to the different regions/voxels in the total set of brain regions R; $\propto$ is used to indicate that the two scale with a fixed constant k. We present a visual representation of the steps of sLNM in Supplementary Figure S1. In addition, in Supplementary Note S19 we provide a detailed mathematical derivation. With the variance of connectivity of brain regions to be approximately equal across brain regions, k can be taken constant across all regions with only an effect on the magnitude of the correlation values not the pattern across R.

Replacing now Cm further with $M \times C$ (Step 1 and 2, Equation 3 of the main text) results in

$$\text{sLNM} \propto sv \times (M \times C), \qquad \text{(Equation S1)}$$

with sv the standardized row vector of the symptom scores, M the lesion matrix and C the group connectivity matrix. As such, sLNM can be seen as a weighted version of the standard LNM, where the lesions are weighted with the symptom scores in sv. We provide a MATLAB exemplary code to show the scaled equivalence between the r-map implementation and Equation S1 ($r = 0.97$, $n = 100$ lesions).

It follows from Equation S1 that the sLNM variant thus similar to LNM samples data repetitively from one and the same connectivity matrix C. Consequently, the resulting sLNM r-map is likewise prone to reflect only basic properties of C. It is important to note that sv needs to be zero-centered to derive at Equation S1, meaning that its weights include equal numbers and sum of negative and positive values. When summing over all weighted rows, the connectivity vectors of individual lesions are therefore no longer uniformly positive but contribute positively or negatively depending on the sign and weight of sv. At first glance, across the weighted rows, this is expected to converge to zero, and thus the values across brain regions R would not automatically display a pattern or ordering.

This theoretical independence can break down in practice when C is low-dimensional. In a low-rank situation (that is, a dataset where the variance in the examined data can be described by a limited number of underlying latent factors), the computation of the correlation between sv and all columns of Cm yields a restricted range of possible patterns. This arises because mapping a high-dimensional factor (sv) into a lower-dimensional space (low-rank C and Cm) necessarily reduces the expressive variety of possible outcomes, with distinct high-dimensional configurations collapsing to one and the same lower-dimensional representation.

Matrix C representing the functional connectivity architecture of the brain is a low-rank matrix, structured according to a degree, modular and gradient-like structure of brain functional connectivity. Accordingly, a PCA reveals that the first principal components (PC1-3 reflecting the first cortical gradients) of the GSP1000 C matrix explains a substantial proportion (first 3 PC/gradients > 70%, first 5 > 80%) of the variance of the regional connectivity profiles in C. These latent factors are often referred to in literature as gradients of functional brain organization [54]. The first dominant components of C for example reflects the degree of C (PC1, $|r| = 0.82$) and the brain's resting-state FC modular architecture [55]. Hence, even when the symptom vector sv has no true relationship with the values in Cm, its pattern across the brain in sLNM will randomly align—positively or negatively—with the limited factors describing the structure of C. This is particularly the case for the first component, which explains the most variance of the connectivity profiles of the regions of C. Consequently, applying sLNM to a set of lesions and a symptom vector systematically incorporates traces of the main components of C, most prominently the latent factors explaining the most variance of the connectivity profiles of C.

Empirical validation. We validated the above derivation (see also Supplementary Note S19) empirically by testing published LNM maps that were based on using sLNM, followed by a simulation where we show the above described consequence of sampling from low-rank *C* (next paragraph). We downloaded published sLNM maps for e.g., post-traumatic stress disorder [2], depression [1], treatment for anxiety-depression symptoms [3], depression circuit in multiple sclerosis [7], political involvement [11], DBS-derived LNM maps for obsessive-compulsive disorder [14], cognitive decline in Parkinson's disease [15], and tremor [16] (all data sources listed in Supplementary Table 2). As a consequence of the first component (PC1) of *C* to predominantly describe degree of the GSP1000 FC matrix ($|r| = 0.82$), these sLNM maps showed a strong trace of voxel-wise degree of the normative connectome, as predicted ($|r|$ = 0.27-0.70, $p < 0.001$, $p_{spin} < 0.01$, $p_{brainsmash} < 0.001$). Furthermore, evaluating also other basic properties of *C,* the spatial pattern of the published sLNM maps could again be explained in terms of only a linear mixture of basic properties of *C,* with elementary factors of *C* (sub-cortical and cortical degree, basic modular and gradient structure PC1-PC3; see Supplementary Note S13) explaining on average as much as 79% of the variance in each of these maps (R^2 = 0.57-0.97).

We also observed the same effect when re-analyzing published lesion/seed and clinical data. We re-applied sLNM to a dataset reporting individual MNI coordinates representing TMS stimulation sites with respect to treatment efficacy of neuromodulation therapy of patients with depression [52, 56, 57] and a dataset with lesions associated to recovery of aphasia ($n = 227$) [58] (no sLNM paper has been reported on this aphasia dataset as we know, but the dataset was used as a second validation dataset). We downloaded the reported MNI coordinates of dorsolateral prefrontal cortex (DLPFC) TMS locations [52, 56] and aphasia lesions with corresponding patient symptom scores. These coordinates/lesions were processed with Lead-DBS, and performed sLNM with *sv* describing the provided patient clinical efficacy scores. The resulting sLNM *r*-map revealed significant alignment with the voxel-wise degree ($|r| = 0.53$ and $|r| = 0.50$, respectively p, $p_{spin} < 0.001$) and PC1 of the GSP connectivity matrix ($|r|$ = 0.84/0.71).

Simulation. By means of randomization and simulation we further examined whether this overlap would occur systematically, as would be predicted from Equation S1 and Equation 4. We again examined the application of sLNM to the TMS DLPFC stimulation sites as an example. We re-run the sLNM procedure 1,000 times, but now each time shuffling the values of *sv* and computing the matching voxel-wise *sLNM_random* map and correlating the overlap with 1) the sLNM DLPFC map (obtained with the real *sv* [52, 56]) and 2) with the voxel-wise GSP1000 degree map. Across 1,000 runs, 74% of the runs using randomized *sv* showed a correlation of $|r| > 0.3$ with the original map ($p < 0.001$) and 80% resulted in a significant correlation higher than $|r| > 0.3$ with PC1. We provide a code example of this in S20. As further expected, the model of basic properties of *C* (see main text and Supplementary Note S13) explained on average 75% of variance (SD = 13%) of the random sLNM maps.

To further illustrate that this behavior of sLNM occurs systematically due to the low dimensionality of *C*, irrespective of the lesion set at hand, we computed for 1,000 times a random sLNM map, each time now randomly selecting $n = 50$ lesions matching 50 random rows from the connectivity matrix *C* and using each time a random vector *sv* (e.g., random numbers $sv \sim N(0,1)$). As the direction of the projection of *sv* on *Cm* is randomly positive or its complete inverse, we aligned the *r*-maps by flipping the sign of the (random) correlation values when the projection was $r < 0$. As expected, across the 1,000 runs, 74% of the random sLNM maps showed $|r| > 0.3$ with degree (PC1 of *C*) with 85% (SD = 11%) of the variance in the spatial patterns of the resulting sLNM maps explained if we further incorporated the other basic properties of *C*. We observed the same phenomena for the other empirical sLNM maps

(Supplementary Table 2), which similarly revealed a strong similarity between the original sLNM maps and their random counterparts. We computed 1,000 random sLNM maps based on random lesions (n = 50) and random *sv* scores and computed the main shared component across the random runs (using PCA and taking the first component across the random runs). Empirical sLNM maps (n = 14, Supplementary Table S4) showed a strong correlation to the dominant pattern of their random counterparts with an average correlation of mean $|r|$ = 0.57 (SD = 0.28). This illustrates that predictable outcomes of sLNM are expected when applied to sets of lesions and symptom scores, whether they are patient informed or whether they involve random lesions and/or random symptom values. We provide a code example in S20.

Group-contrast symptom based LNM. We note the existence of a third LNM variant, sometimes referred to as *"lesion-symptom network mapping"* [50, 51, 59, 60] where the lesion functional connectivity maps are compared between dichotomous conditions or patient sub-groups [61-63]. Examples include a comparison of lesion maps between patients with (e.g., group *a*) and without (group *b*) remission of smoking [19]. The comparison between groups in this variant is often performed by means of a two-sample *t*-test on the individual LNM Fisher *r*-to-*z* maps or *t*-maps across the groups or by fitting (which is equivalent) a linear regression model. The sLNM map is the resulting map obtained by contrasting the two groups. In terms of *M* and *C,* this variant can be described as sLNM ~ ttest($Ma \times C$, $Mb \times C$) between two defined groups *a* and *b*. A pooled two-sample *t*-test is mathematically equivalent to a linear regression with a binary predictor, making this approach similar to the sLNM variant that uses a symptom vector *sv* as a contrast vector with e.g., group *a* defined as 1 and group *b* as -1 (the only difference with using a binary vector [0 1] is that the fitted coefficients are halved, the *t*-statistic and *p*-values remain identical). Indeed, the contrast sLNM map published for remission to smoking addiction again strongly aligned with the summation vector of matrix *C* (r = 0.63, p, $p_{spin} < 0.001$).

LNM on MNI coordinates. LNM was originally defined as a method to study underlying networks of heterogeneous lesion locations, but a large body of studies have also started to apply the LNM approach to MNI coordinates of brain atrophy [12] or functional activation [28] (17 studies identified, Supplementary Table 1), or cortical alterations [24], with the same rationale to study how spatially heterogeneous alterations converge onto common underlying circuits [8, 12, 27, 28, 30]. The MNI coordinates can be derived from meta-analyses of a given condition, for example voxels identified in large-scale Voxel Based Morphometry (VBM) studies [12] or from functional activation meta-analyses [28], coordinates of extreme cortical deviations identified by normative modeling [24], or normalized MNI coordinates of TMS locations of individual patients [56]. Compared to lesion studies, which generally involve a relatively small number of lesions (< 50-100), most coordinate-based LNM studies analyze a much larger set of coordinates. Applying standard LNM, running each coordinate as a lesion through the LNM pipeline, LNM circuit maps derived from coordinate-based LNM studies are equally affected by the discussed limitations of the methodology. Throughout the main text, we describe several of these studies in which the derived LNM maps of e.g., depression [30], schizophrenia [24], creativity [27], epilepsy [8], facial emotional processing [28] strongly align with the summation vector of *C* (r = 0.58-0.97, p, p_{spin}, $p_{brainsmash} < 0.001$). See main text and Supplementary Table 4 for all *r*-values and statistics.

S10. Spatial convergence of LNM networks to degree of *C*

The overlap between each LNM map and functional degree of the normative connectome was calculated as follows. The normative connectome *C* was derived from the preprocessed functional connectivity of the GSP1000 dataset [35]. For the voxel-wise analysis, the degree of

each cortical (and as a validation analysis, also subcortical, and cerebellum) voxel was computed as the sum of the functional connectivity with all other voxels in the brain. Overlap between each condition's LNM map and the global degree map was quantified by means of the Pearson's correlation coefficient calculated across all voxels in the brain mask. An atlas-based LNM analysis was conducted by parcellating the brain according to the Yeo-Schaefer1000 atlas [25] and Melbourne54 Subcortex Atlas [26]. The degree of each region was calculated as the sum of its functional connectivity with all other regions, and overlap with the atlas-based LNM map for each condition was quantified using Pearson's correlation at the atlas level. Spin-null permutation [40] was used to further assess statistical significance accounting for spatial autocorrelation effects (10,000 permutations). For each condition, the correlation was computed between the LNM map and the row sum of the subset of the normative connectome *C* corresponding to the voxels (or regions) affected by lesions. This analysis assesses the extent to which LNM maps reflect the inherent structure of the sampled connectivity matrix, particularly in cases with few or spatially clustered lesions (Figure 4). Results are reported in the main text; all correlations and *p*-values are reported in Supplementary Table 4.

Effect of lesion set size. We examined the number of spatially heterogeneous lesions that would be needed to obtain a significant trace of degree in the group-analysis LNM map ($|r| > 0.3$, $p < 0.001$, $p_{spin} < 0.05$). We simulated synthetic lesion sets of increasing size (n = 1-150) for 1,000 iterations, with varying lesion size (1, 10, 25, 50) and computed for each set the correlation between the atlas-based LNM and degree of the connectome matrix *C*. Results are shown in Supplementary Figure 2, showing that already at small sets of $n > 10$ spatially heterogeneous lesions the LNM procedure significantly converges to degree of *C*.

Very small set size/spatially homogeneous/focal lesions. The above simulation assess the convergence to degree for the examination of a set of spatially heterogeneous brain alterations, the typical starting point of LNM studies (our literature survey, see section S1 above, indicated that 50-55% of work make an explicit statement on the heterogeneity of the examined lesions/brain alterations as the motivation for the study) (e.g., [8, 9, 24, 27, 28, 30, 64-66]).We also examined the case of when lesions would be more focal and/or homogeneously placed, or equally, when the set of lesions would show high spatial proximity. We note that these cases show resemblance of an investigation of a very small lesion set with all lesions in close proximity, with in the most extreme case all lesions falling into the same area resulting in the same LNM map as the investigation of a single lesion. Here, the LNM map would resemble a single row of matrix *C* (the first example case we present in the main text). We examined whether these situations would also carry a trace of degree, by computing the correlation of each row to the matrix sum of *C* (Yeo-Schaefer1000). In total, 74% of these cases showed a correlation $|r| > 0.3$ ($p_{spin}<0.001$) suggesting that even in the extreme case of highly focal lesions, where one or more lesions are restricted to a single atlas parcel, the resulting LNM map is still highly likely to retain a detectable trace of degree.

S11. Lesion overlap and LNM similarity within and between conditions

The effect of lesion overlap on LNM networks similarity was examined. In the LNM procedure, lesion sets that spatially overlap will naturally yield more similar LNM networks, since lesions in the same regions produce comparable functional connectivity profiles. To rule out this potential confound, we quantified lesion and LNM network overlap across all conditions with available lesion data. Within each condition, the sum of lesions across subjects was computed for each voxel, and Pearson's correlation was used to quantify the similarity of lesion distributions across conditions. Overall, lesion overlap between conditions was low (mean $|r|$ = 0.04, *SD* = 0.05), while LNM network similarity was substantially higher

(mean $|r|$ = 0.42, SD = 0.27). Even when restricting to the condition pairs with the 10% lowest lesion overlap (mean $|r|$ = 0.001, SD = 0.0007), LNM networks remained highly similar (mean $|r|$ = 0.42, SD = 0.23). For example, lesions associated with freezing of gait [67] showed negligible overlap with those linked to hypersomnia [22] (r = -0.0005) and insomnia [22] (r = 0.002), yet their LNM networks were highly similar (r = 0.79 and 0.73, respectively, p, p_{spin} < 0.001). Likewise, migraine [31] lesions showed minimal overlap with those involved in vertigo [68] (r = -0.001) and neglect syndrome [22] (r = -0.00005), while still yielding highly similar LNM maps (r = 0.86 and 0.82, respectively, p, p_{spin}< 0.001).

S12. Similar networks systematically result running replication analyses
Equation 3 explains the consistency of LNM maps reported across common validation approaches used in LNM studies—such as split-half analyses, replication in independent datasets, and other robustness checks [1, 2, 8]—with random sets of spatially heterogeneous lesions converging on essentially the same LNM map, one that reflects the degree sequence of the underlying connectivity matrix (Supplementary Figure 3). Hence, robustness analyses, such as comparing maps between cohorts [1, 2, 7, 8, 20, 69, 70], sub-group analyses [9, 28, 71], and leave-one out or split-half cross-validations [11, 72, 73] will inevitably yield high consistency, reflecting effects of the underlying methodology rather than consistency in the data.

S13. Regression model
A simple regression model describing only the elementary factors of the connectome matrix C was formed. Subcortical degree was computed by selecting the subcortical areas from the Melbourne54 atlas [26] and computing their degree as the sum over the selected regions. Total degree was computed as the total sum over the matrix C. Modular degree was computed by performing modular decomposition to the GSP1000 matrix C (Newmann's modularity, Brain Connectivity Toolbox [74], four modules identified) and the degree of each of the modules was computed as the sum over the regions. The brain's gradient structure was captured by taking the first three gradients of a principal component analysis on C [54]. Together, these nine factors describe the well-documented global structure of the human connectome. We note that an even simpler model with less factors would perform almost as equal. The first gradient is reflecting the degree of C ($|r|$ = 0.82), and the four modules combined also provide the total sum of the matrix. As such, a model of seven factors (subcortical degree, four modules, two components) performs almost as equal, showing that even fewer factors are needed to describe the variance in LNM and sLNM maps. For simplicity, we refer to the nine factors in the main text and analyses. We discuss the contribution of degree, modularity and gradient structure on LNM in more detail below in S14.

S14. LNM (non) specificity
We examined the level of purported disease specificity of LNM as follows. In Supplementary Note S15 we discuss the statistical specificity test as part of the LNM procedure from a more technical perspective.

Uniqueness of LNM maps relative to other LNM maps. The main analysis showed that many of the LNM maps exhibited a high spatial overlap with other presented LNM maps. To quantify this, we correlated each presented LNM map with all other LNM maps (n = 102), demonstrating that LNM maps showed on average a correlation of $|r|$ > 0.6 to 24 other LNM circuits. We further tested for disease specificity by taking each LNM and then examining how much variance would be left by regressing out the spatial overlap of the other voxel-wise LNMs, with this leftover variance reflecting the level of specificity of the LNM map of interest. Across all voxel-wise LNM maps, 98.7% of the variance could be explained by the

other maps, with only 1.3% of the variance, the disease *specific* part, left unexplained by the LNMs of the other disorders (*SD* = 2.5%), indicating an extreme low level of possible disease specificity of LNM maps.

Degree. We next correlated each of LNM maps (Supplementary Table 2) with the voxel-wise sum of degree obtained from the normative GSP1000 dataset. 78 of the 102 LNM maps showed a significant overlap with the degree map ($p_{spin} < 0.05$; mean $|r| = 0.59$, $SD = 0.23$). On average, 33.4% of the variance in the available voxel-wise LNM maps could be explained simply by the sum of all voxel-wise correlations (which is, FC degree) alone, with some of the maps explained by degree as much at 70% and higher (addiction to smoking: 57% [19], epilepsy: 71% [8], vertigo: 54% [68], amusia 65% [75]). We show that this level of explained variance by simple properties of matrix *C* is even further increased when also basic properties as the modular architecture of the connectome *C* are taken into account (e.g., see S13 and below).

Gradient and modular architecture. We examined the remaining level of variance by taking into account the global resting-state and gradient architecture of the functional connectome (see main text, section *Specificity and explained variance of LNM networks*). Resting-state networks (RSNs) and functional gradients were determined by applying modular decomposition to the connectivity matrix *C*, revealing four major RSNs, combined with three components describing the principal functional gradients of the FC connectome [54] derived by principal component analysis. A general linear regression was applied to each LNM map, with a basic linear model consisting of cortical and subcortical degree, the degree of each of the four identified RSNs, and the three principal functional gradients as independent variables. This linear model, irrespective of lesion location and/or association with any specific condition, explained 93% (*SD* = 5.0%) of the variance in the examined 80 atlas-based LNM maps where lesion data was available (results presented in main text). For completeness, we also computed the mean explained variance across all 102 voxel-wise LNM maps available, which also included signal from lesions and parts of lesions overlapping the white matter which could not be modelled by the atlas-based method. Across the 102 LNM networks, the mean explained variance was 84% (*SD* = 9.1%). This demonstrates that the majority of the signal in each re-analyzed LNM map can be attributed to the degree and modular structure of the FC connectome, with none of the LNM maps showing disease-specific information. In fact, the remaining level of unexplained variance falls well within the level of methodological noise that can be expected in rest-state fMRI and functional connectivity data [76].

Network matrix C. After demonstrating the absence of specific information in the LNM maps, we addressed a potential follow-up question of whether the non-specific convergence of LNM maps to the degree of *C* might be biologically meaningful. To test this, we examined the application of LNM using multiple versions of matrix *C*. Equation 3 (main text) predicts that convergence occurs towards the summation vector of *C* in all cases and, in cases of lesion overlap, also further to the modular structure of *C*, irrespective of which matrix *C* is used. We tested this, by replacing *C* with various artificially generated connectivity matrices *C'*, including synthetic Barabási scale-free, modular, and star-like networks (Supplementary Figure 3). Indeed, in all cases, LNM applied to sets of real lesions converged completely to the degree distribution of the presented matrix *C'*, indicating that convergence to the degree distribution of *C* is a consequence of the LNM method itself and not indicative of a biologically meaningful signal. Results are shown in Supplementary Figure 3.

S15. Extended discussion on LNM statistical procedures

LNM studies typically report the obtained LNM maps and a series of statistical tests aimed at assessing the sensitivity and specificity of the observed effects [21, 77, 78] (Supplementary Figure 5). Below, we examine these statistical procedures in light of our findings that LNM outcomes largely reflect the degree distribution of the normative connectome *C*. As we will demonstrate, LNM analyses will almost invariably yield statistically 'significant' results.

Sensitivity Test. The first step in the LNM statistical pipeline is the sensitivity test. Implemented in the Lead-DBS toolbox [34] this involves: (1) performing a one-sample *t*-test to assess whether the functional connectivity of each voxel with a lesion significantly differs from zero across the 1,000 GSP1000 subjects of the normative dataset *H*; (2) thresholding the resulting *t*-scores (commonly using $|t| > 7$, correcting at Bonferroni level [49]); and (3) identifying voxels that surpass this threshold in a majority of lesion cases (often ≥ 75% [46]).

Although earlier methodological studies have questioned the validity of the $|t|$ threshold [79], some have argued that LNM results remain stable across thresholds [49]. However, we and others [79] note that these *t*-scores trivially scale with the size of the normative sample *H*. For instance, comparing *t*-maps derived from the same lesion using 100 versus 1,000 subjects in the GSP dataset yields identical spatial patterns ($r = 0.99$, $p < 0.001$), with *t*-values differing only by a fixed factor. This dependency indicates that the threshold should be manually adjusted when performing LNM on different sized normative datasets, which undermines the reliability.

Regardless, this step adds little statistical value: the one-sample *t*-test can be replaced with the mean connectivity map, which yields equivalent spatial patterns ($r = 0.99$, $p < 0.001$, based on 50 random lesions). A $|t| \geq 7$ threshold, though seemingly conservative, frequently results in widespread significance simply due to the sample size of the normative dataset. Running 50 randomly selected lesions through Lead-DBS, we found that on average 64% of the voxels in the brain exceeded $|t| > 7$, illustrating the method's low specificity.

The third step in the LNM sensitivity analysis involves applying a group-level threshold *G*, selecting only voxels that exceed the $|t| > 7$ threshold in a defined proportion of lesions, commonly 75% [46], which can be considered as performing a binomial statistical test. This step aims to identify voxels with consistent functional connectivity across the lesion set. While this joint thresholding again appears stringent, it is not uncommon for voxels to surpass both thresholds, especially when lesions exhibit minimal spatial overlap. We examined this as follows. 50 synthetic lesions were simulated and randomly distributed across the brain, and LNM was conducted using the Lead-DBS toolbox. As expected, many voxels exceeded the $|t| > 7$ threshold individually (see above), but none surpassed the combined $|t| > 7$ and group threshold of $G = 75\%$, as intended. However, introducing even minimal overlap between lesions (average Dice coefficient between lesions = 0.1; see Supplementary Figure 6) was sufficient for voxels to start meeting both thresholds. With moderate overlap (Dice = 0.25–0.30), the number of voxels exceeding the joint sensitivity threshold increased rapidly, reaching over 1,000 voxels. We will return to the implications of this observation after discussing the specificity test in the LNM procedure.

Specificity Test. The second statistical step in the LNM procedure assesses the specificity of the results [77, 78], typically through a two-sample *t*-test comparing the correlation maps of lesions of interest against a large set of control lesions unrelated to the condition. While this appears to serve as a valid null test, it is important to note that the specificity test is not statistically independent from the sensitivity test. As shown above, LNM applied to any heterogeneously distributed lesion set will converge to the degree of the normative connectome *C*. Consequently, the specificity test effectively compares a similar degree-related signal as tested in the binomial group test in the sensitivity test, but now tested against a different baseline. Rather than contrasting the lesions of interest with a meaningful control

condition, this thus results in a test of the same underlying signal. We confirmed this lack of independence by performing LNM on both real and simulated lesion sets, revealing strong correlations between sensitivity and specificity maps ($r = 0.37$, $p < 0.001$).

The specificity test is presented by LNM studies [77, 78] as a specific test to evaluate the level of specificity of the LNM map regarding the lesions associated with the disorder of interest. Many LNM studies attempt to test this level of specificity by contrasting the LNM map obtained from the lesions of interest to a LNM map resulting from running a large set of lesions not related to the condition of interest [4] or, as an alternative, randomizing the lesion or voxel coordinates across the brain [30]. In many (but not all) of studies, a large set of control lesions is taken (often from the Harvard Lesion Repository, a closed-source database), with lesions coming from all different types of sources and related to all sorts of non-related conditions. This examined control set is often of size n = 500-1000 lesions. We note that in some occasions, LNM studies directly contrast lesion sets between specific conditions [9, 12, 20], for example contrasting an LNM map calculated on the basis of lesions associated with criminality with lesions associated with neurodegenerative disorders.

Following Equation 1, 2 and 3 from the main text, computing LNM on a large number of lesions, randomly associated to a variety of conditions, is equal to selecting random rows from the connectivity matrix C. With lesions randomly placed, mostly covering the entire brain, each lesion matches a random row in C and hence the set as a whole is taking a random, large, subsampling of the rows of C, leading to a joint mean LNM map matching the summation vector of C. Indeed, to approach the control lesion dataset of the Harvard Lesion Repository, we took the collection of segmented lesions analyzed in this study (Supplementary Table 2), which in total included 1, 285 lesions across 47 conditions. Running these lesions as a control dataset revealed a joint mean LNM map highly correlated to the summation vector of C (atlas-based LNM, $r = 0.75$ ($SD = 0.07$), $p < 0.001$), as expected from Equation 1 and 3 (main text).

Conjunction map. The final step in LNM statistical procedures often involves computing a conjunction map [9, 77], also called a convergence map [4], by combining the sensitivity and specificity tests. Voxels that survive both tests are considered the core regions of the LNM circuitry [4, 9, 77] and are interpreted as having passed a highly stringent statistical threshold [4]. However, we argue that given the ease of surpassing the sensitivity test (as demonstrated above) and the interdependence between sensitivity and specificity scores (noted previously), it is not uncommon for brain regions to meet this conjunction criterion. We tested this as follows. We again simulated lesions, but now we examined 10 sets, each consisting of 50 lesions, randomly sampled across cortical and subcortical areas of the brain. We started by sampling a first set s_1 of 50 lesions, with the lesions completely randomly selected across the brain. We then sampled another 50 random lesions for s_2 to s_{10}, but across the sets, introducing a stepwise increase in the probability of spatial overlap to occur between some of the lesions in the set. This effectively introduced overlap in a small proportion of the lesions in each set, but with the rest of the lesions remaining completely randomly selected across the brain, creating chance-level overlap of 0-5% for s_1 to around 30% overlap for s_{10} (Dice coefficient, see Methods). Indeed, in the case of low levels of lesion overlap, only a very small percentage (0-1%) of brain regions managed to surpass both the joint sensitivity ($|t| > 7$, $G = 75\%$) and specificity test ($|t| > 10$), as is intended by the procedure. However, when the level of overlap between the lesions increased from s_2 to s_{10} the number of regions to survive both tests was rapidly increasing. For random lesions, even mild to moderate levels of overlap (10%-25%) already resulted in 20 to 25 brain regions (out of the tested $R = 1000$, 2% of the brain) to survive the conjunction procedure and together formed a ‘significant’ LNM circuitry (Supplementary Figure 6).

In the above simulation, all lesions were randomly simulated across the brain, meaning that they were not linked to any specific neurological condition or neuropsychiatric disorder. This suggested that many lesion sets could falsely produce an LNM circuit. Along this line of thought, we argued that this might be true for *all* possible sets of lesions. We examined this hypothesis in a final simulation, where we simulate multiple sets of lesions with again varying levels of overlap, but now with their random center point of overlap in all possible locations in the brain (using the Yeo-Schaefer1000 atlas, see Methods). We simulated again 50 lesions per set, for 10 levels of overlap (again with 0 to 0.35 overlap, and the rest of lesions selected randomly across the brain), with the center point of overlap for each of the 1,000 cortical regions (and an additional 54 subcortical), running LNM for a total of around 500,000 lesions. We run LNM on all the lesion sets, computed the sensitivity ($|t| > 7$, $G = 75\%$) and specificity map ($|t| > 10$) using common settings, and finally determined for each set the number of regions that ended up in the final LNM map, without being related to any specific disease condition.

Across all 500,000 tested lesion sets, we found LNM to systematically result in a 'significant' map, surpassing both the sensitivity and specificity test. Even at marginal levels of lesion overlap (mean/*SD* = 0.08/0.02, Dice coefficient), 10% of lesion sets already showed regions that surpass both tests and would be considered to produce a 'significant LNM circuitry'. At minimal overlap of lesions in a set (Dice ~0.15-0.20), 64% of sets turned out to result in a significant circuitry, with 97% of all lesion sets found to form a 'significant' LNM circuitry at moderate levels of overlap (Dice>0.25). At Dice>0.3 levels of overlap virtually all simulated heterogenous lesion sets (>99% of all tested sets) revealed a significant LNM map. We replicated this effect for multiple settings.

These results illustrate that almost any possible sets of lesions with a minimum level overlap can be expected to produce a 'significant' LNM map, surviving the proposed statistical procedures for LNM. This conclusion is consistent with the reports of 'significant' LNM circuitry for lesions associated to many, widely varying brain conditions, from established neurological or mental conditions [4, 9, 77] to more popular science topics such as lesion locations associated with political involvement [11].

S16. Null-models

We examined whether applying LNM to random lesions could serve as a potential framework for developing an improved null-model for LNM, or whether the LNM effects would fail to survive even the most liberal null-model. In the latter case, further efforts to develop more complex and conservative null-models that preserve additional features of the original distribution would be unwarranted, as this is typically the starting point and rationale for refining null models [80].

Generative null-model. As a starting point, we compared LNM maps against a liberal null-model of using random lesions across the brain with random levels of spatial overlap. We generated 500,000 lesion sets, each consisting of 50 uniformly selected locations across the brain, from which lesions of size of 5 parcels of the Yeo-Schaefer1000 atlas (in total describing 1,000 brain areas) were expanded, with the probability of overlap between the lesions in each lesion set randomly determined by uniformly sampling the probability of overlap between zero and one. Practically, this sampled a dense solution space of possible LNM outcomes with zero to maximum level of spatial overlap between lesions in each set. Each of the lesion sets were run through the atlas-based LNM procedure (see main text), resulting in 1,000,000 random atlas-based LNM maps, which were used as a null-distribution to test the regions in all published LNM maps (as listed in Supplementary Table 2) and to examine whether any of the 1,000 regions within each of the LNM maps would exceed this

lenient null-model. Even at a most liberal setting, using a nominal alpha of 0.05 (that is, not corrected for the 1,000 tests performed within each of the LNM maps and not corrected for the 78 atlas LNM maps studied) 70 published maps showed zero regions to pass this simple null-model (either positively or negatively). Setting a nominal alpha of 0.01 only 3 of all examined LNM maps were found to pass the null-condition with a few small brain regions. When alpha was corrected for the 1,000 tests performed (not yet correcting for the LNM maps tested), none of regions in any of the LNM maps passed the null-model.

Permutation null-model. We also tested LNM maps against a permutation-based null-model, where real lesions were randomly permuted, preserving their participation in resting-state fMRI networks. We used the atlas-based GSP1000 matrix for this, based on the data presented in the Lead-DBS toolbox [34] in Yeo-Schaefer1000 atlas resolution. We performed modular decomposition to the GSP1000 matrix *C*, using standard Newmann's modularity as part of the Brain Connectivity Toolbox [74] and selected 16 modules comprising an average of 65 nodes (*SD* = 27). All unique cortical lesions were randomly sampled across the brain, preserving modular participation, for 1,000 permutations, obtaining a null-distribution of random LNM maps for each included LNM dataset. For all LNM maps and across all parcels, it was tested how many of the parcels differed from the null-distributions. FDR was used to correct for the 1,000 tests performed within each lesion set (corrected alpha = 0.05). Out of the 78 lesion sets that could be tested, only 7 LNM maps showed 1 or more regions to reach the significance threshold, 71 maps showed no effects across the 1,000 regions tested. Second, across the LNM maps, we tested whether the pattern of the LNM map would be different from the random maps. Across the 1,000 random runs, the correlation between the original and the random LNM map was computed. Across all runs and tested maps, the average correlation between the real and random LNM maps was 0.93 (*SD* = 0.052), indicating that in the majority of cases the LNM maps derived from random lesions showed highly similar maps to LNM derived on the basis of patient lesions. We finally tested whether clinical and random maps would be similar- i.e., $r > 0.8$ correlation between the clinical and random map. Performing this for 1,000 random runs, it was examined whether the LNM map would differ from at least 50 of the 1,000 random maps with a liberal alpha of 0.05. Results showed that 76 out of the 78 tested LNM maps were statistically indistinguishable from their random counterparts.

Supplementary Figures

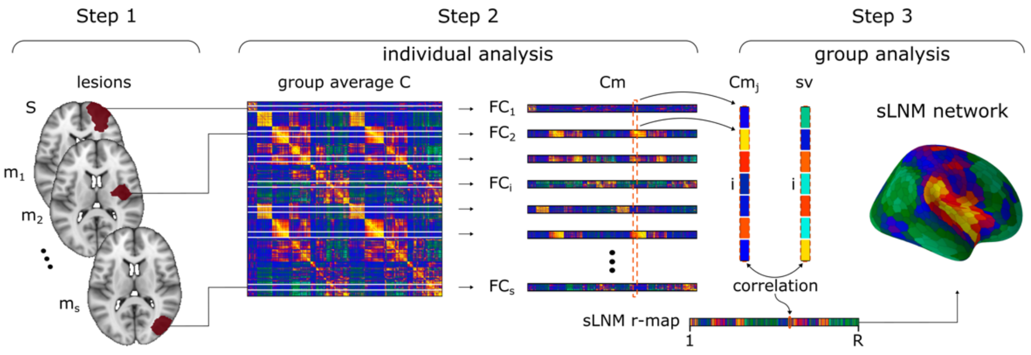

Supplementary Figure 1. Symptom Lesion Network Mapping. From left to right: Similar to the LNM approach, individual lesions are processed in Step 1 and Step 2 of the symptom Lesion Network Mapping (sLNM) procedure, and can be similarly described by selecting matching row(s) *i*, corresponding to lesion *m* of patient *s*, from the group average connectome matrix *C* , and repeating this for across all subjects *S*. This results in the row matrix *Cm* (middle column). Next in the group-analysis Step 3 of the sLNM procedure, for each voxel or region *j* in the brain mask (i.e., the columns of *Cm)* the functional connectivity values of the selected lesions in column *j* of *Cm* are correlated with the patients' symptom values *sv*, yielding a correlation coefficient per region *j*. This is repeated for all regions in the brain mask and these coefficients across all brain regions together form the group-analysis sLNM *r*-map. C, group connectivity matrix; FC, functional connectivity; R, number of voxels or regions in the used brain mask; r, correlation coefficient; s, patients; sLNM, symptom Lesion Network Mapping; sv, symptom vector.

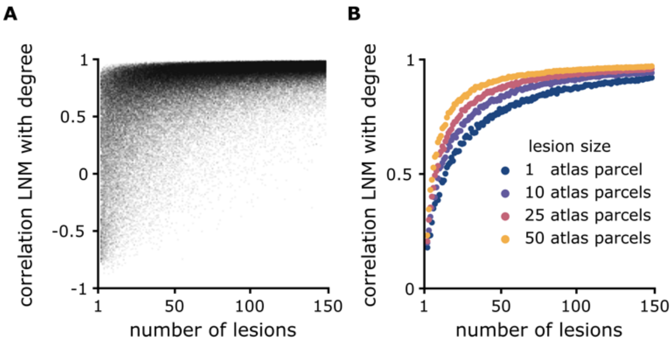

Supplementary Figure 2. Lesion Network Mapping converges to the degree of the functional connectome as a function of lesion number. (A) Simulations were conducted using 1,000 sets of randomly distributed single-parcel lesions on the Yeo-Schaefer1000 and Melbourne54 atlas. For each lesion count from 1 to 150, corresponding Lesion Network Mapping (LNM) maps were computed and correlated with the degree of the normative connectome *C*. Each dot represents one simulation. As the number of lesions increases, more rows of *C* are sampled, and the correlation (*r*) with the degree map approaches one. (B) The same analysis was repeated with increasing lesion sizes (10, 25, and 50 parcels), defined by selecting a random parcel and its nearest neighbors. Dots indicate the mean correlation across 1,000 simulations for each lesion size and count. Larger lesions sample more rows of *C*, leading to faster convergence toward the degree map. LNM, Lesion Network Mapping.

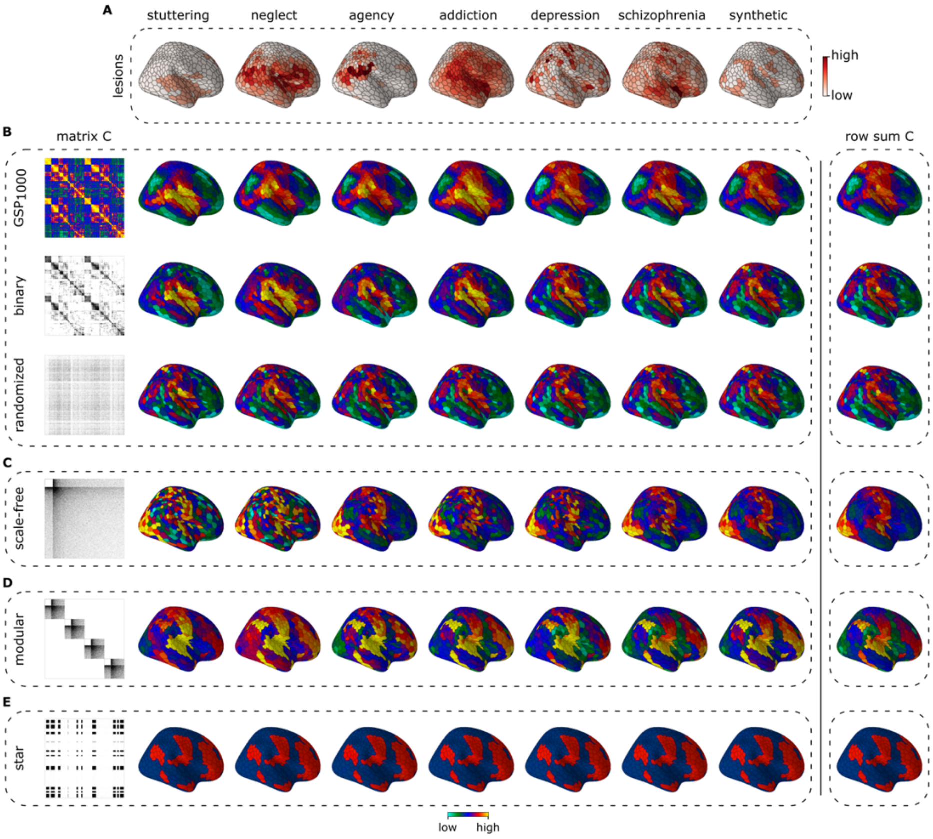

Supplementary Figure 3. Lesion Network Mapping converges to the row sum of the used normative connectivity matrix, regardless of its biological validity. Panels show Lesion Network Mapping (LNM) analysis of lesion or cortical deviations associated with different conditions discussed in the main text (from left to right: neurogenic stuttering [81], neglect syndrome [22], disrupted agency [21], addiction [19], depression [24], schizophrenia [24], and a set of 50 synthetic simulated random lesions based on the Yeo-Schaefer1000 atlas), using multiple versions of matrix *C* and synthetic matrices *C′*. (A) Lesions associated with each condition. Red indicates regions with high lesion overlap across patients; white indicates low overlap. (B) First row: LNM sensitivity maps derived using a weighted functional connectome matrix from the GSP1000 data. Second row: LNM sensitivity maps derived using a binary functional connectome matrix ($r > 0.2$) from the same dataset. Third row: LNM maps computed using the same lesion sets, but with a Maslov–Sneppen-randomized version of the GSP1000 functional connectivity matrix *C*, preserving the row sum (degree sequence) of *C*. This panel highlights the high similarity of LNM results across conditions (left to right) and the high similarity of maps derived using the randomized connectome. (C-E) LNM applied to the same lesion sets as in panel B, using alternative connectome matrices: a synthetic Barabási–Albert scale-free network (panel C; toolbox: [82]), a synthetic modular network with four distinct communities (panel D), and a synthetic star-network (panel E). In all cases, LNM converges to a consistent network map across all diverse conditions examined, reflecting the row sum structure of the respective adjacency matrix *C′*. Note that while the row sum distributions differ across the randomly generated variants of *C′*, the lesion sets consistently yield similar LNM maps across conditions (left to right). The most right column shows the row sum of the matrix *C* and *C′,* illustrating all LNM runs to converge to the row sum of the used connectivity matrix *C′*. LNM, Lesion Network Mapping.

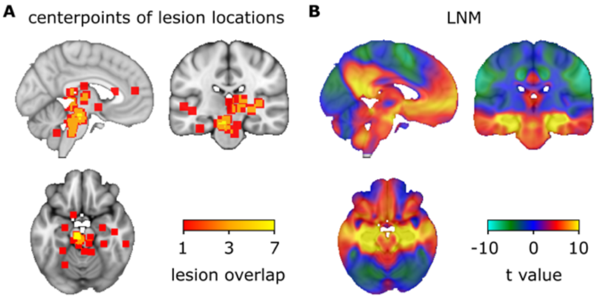

Supplementary Figure 4. Lesions associated with psychosis. (A) Brain plots depict the spatial locations of the center points of psychosis-inducing lesions, identified from [4]. Each lesion center point was estimated from published images and dilated by 5 voxels in all directions, ensuring equal size across lesions. Lesions were most frequently observed in the midbrain (33 out of 104, 32% of total) and thalamus (22 out of 104, 21%) (B) Published Lesion Network Mapping (LNM) map related to psychosis from [4]. LNM, Lesion Network Mapping.

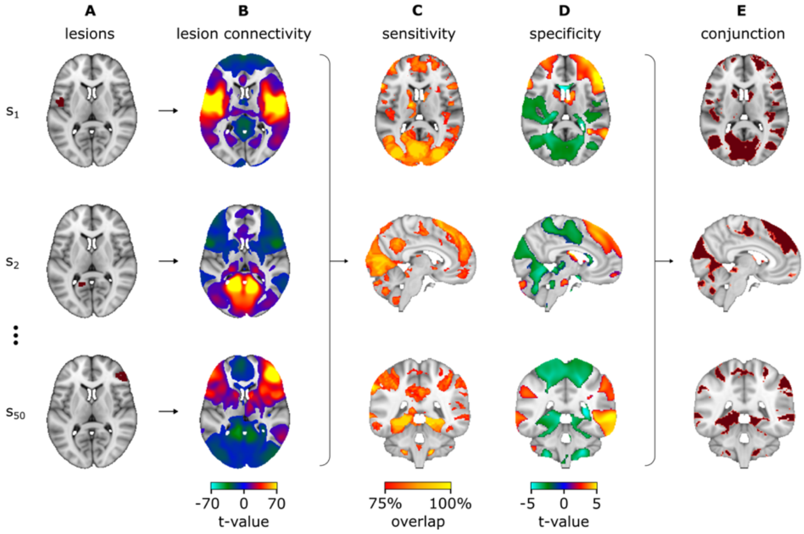

Supplementary Figure 5. Sensitivity, specificity, and conjunction test. Key statistical steps involved in Lesion Network Mapping (LNM) analysis. Each row represents a different simulated lesion from a set of 50 lesions (s_1, s_2,.. s_{50}), with columns (A-E) illustrating sequential steps in the LNM statistical testing pipeline. (A) Spatial location of the simulated lesions. (B) Functional connectivity maps derived from each lesion using a normative connectome. Each voxel is tested for significant connectivity ($r > 0$) across the normative sample. (C) *Sensitivity* test identifies voxels where lesion t-maps exceed $|t| > 7$ and where connectivity is observed in more than 75% of cases. (D) *Specificity* test determines whether observed connectivity patterns are specific to the lesion group by comparing r-maps with those from an independent reference lesion set (e.g., across various conditions) via a voxel-wise two-sample t-test. (E) *Conjunction* map combines the sensitivity and specificity maps, identifying voxels that meet both criteria, defining the core network associated with the lesion pattern. s, patients.

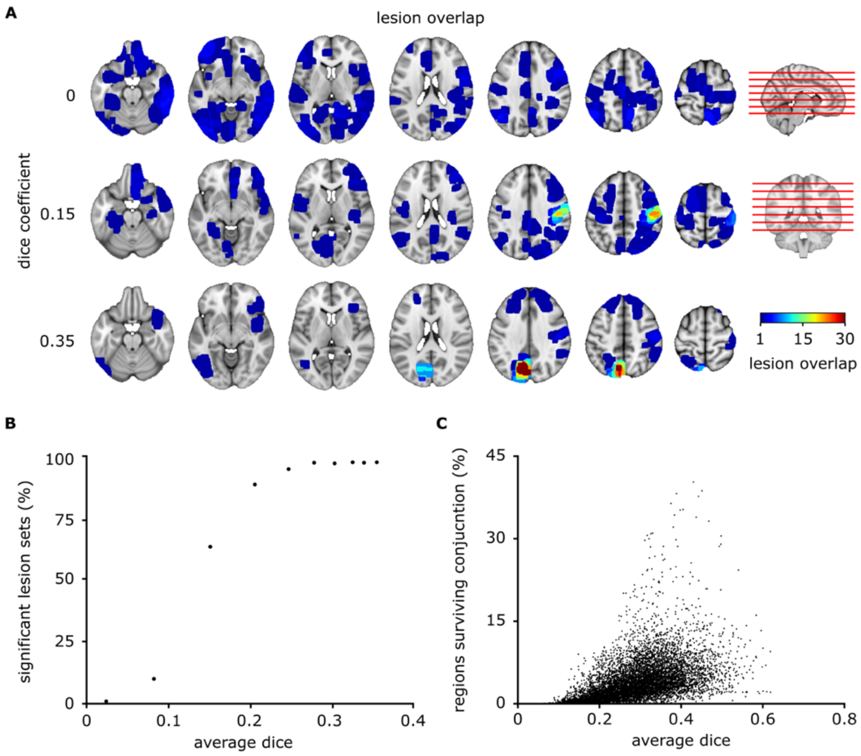

Supplementary Figure 6. Spurious significance of Lesion Network Mapping conjunction maps driven by lesion overlap. (A) Spatial overlap of lesions from three example simulated datasets (50 lesions each) with increasing levels of spatial overlap, quantified using pairwise Dice coefficients (~0, ~0.15, ~0.35). Upper panel: lesions randomly distributed across the brain with near-zero overlap. Middle panel: mild overlap among a subset of lesions. Lower panel: moderate spatial overlap. In total, 50 lesion sets were generated for each of 10 overlap levels (Dice coefficients 0–0.4), using all 1,000 regions of the Yeo-Schaefer1000 atlas. (B) Lesion Network Mapping (LNM) sensitivity, specificity, and conjunction maps were computed for all simulated lesion sets. Panel shows the proportion of simulated lesion sets (out of 1,000) per overlap level (y-axis) yielding a significant conjunction map, defined as surviving both sensitivity ($|t| > 7$, group threshold ≥ 75%) and specificity ($|t| > 10$) tests. Even low to moderate lesion overlap significantly increases the likelihood of passing the conjunction test, despite lesions being randomly assigned and unrelated to any actual condition, highlighting substantial inflation of statistical significance in LNM. (C) Percentage of brain regions (out of 1,000) identified as part of a 'significant' LNM circuitry (surviving conjunction test; y-axis) as a function of lesion overlap (average Dice coefficient). As overlap increases, the number of brain regions identified in the significant LNM conjunction map rises considerably, further underscoring how lesion clustering alone can drive inflated LNM results even in the absence of meaningful underlying pathology. Sensitivity test settings used were $|t| > 7$ and a group threshold of 75%; specificity test setting was $|t| > 10$.

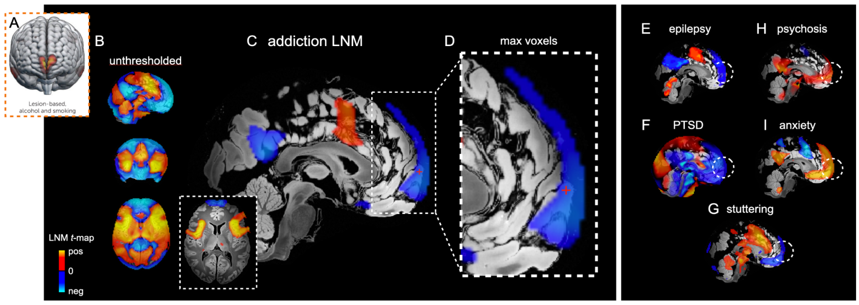

Supplementary Figure 7. Lesion Network Mapping proposed transcranial magnetic stimulation site for substance abuse disorder. Studies have openly advocated the use of Lesion Network Mapping (LNM) to target new stimulation sites, based on 'most negative' (for excitatory transcranial magnetic stimulation [TMS]) and 'most positive (for inhibitory TMS) sites in the LNM maps. The main analysis shows that LNM maps, and with that their negative and positive areas are only due to the degree and modular structure of the connectome, with the LNM pipeline resulting in trivial results. Comparison analysis between LNM maps of different disorders

shows that the LNM maps are indeed non-specific, providing no biological evidence or support for the most positive or most negative areas as TMS target sites. (A) Panel shows a figure from Joutsa et al. on LNM on lesions associated with smoking remission [19], with the frontal pole proposed as a new target area for TMS for substance abuse disorder. (B) Panel shows the LNM map as downloaded from [19], unthresholded. (C, D) Panels show the same LNM map as in B, now thresholded to indicate the highest negative (and highest positive) areas in the LNM map, with the red + indicating the target stimulation site as proposed by [19]. (E, F, G, H, I) Following the rationale of using the highest and lowest areas of the LNM map as stimulation sites, the same area would be proposed for treatment of epilepsy [8], post-traumatic stress disorder (PTSD) [2], neurogenic stuttering [81], or inversely for psychosis [4] and anxiety [3]. Panels show the downloaded and computed voxel-wise LNM maps computed for epilepsy, PTSD, neurogenic stuttering, psychosis and anxiety (sources Supplementary Table 2) as derived by means of Lead-DBS [34](see Methods). LNM, Lesion Network Mapping; PTSD, post-traumatic stress disorder; SUD, substance abuse disorder; TMS, transcranial magnetic stimulation.

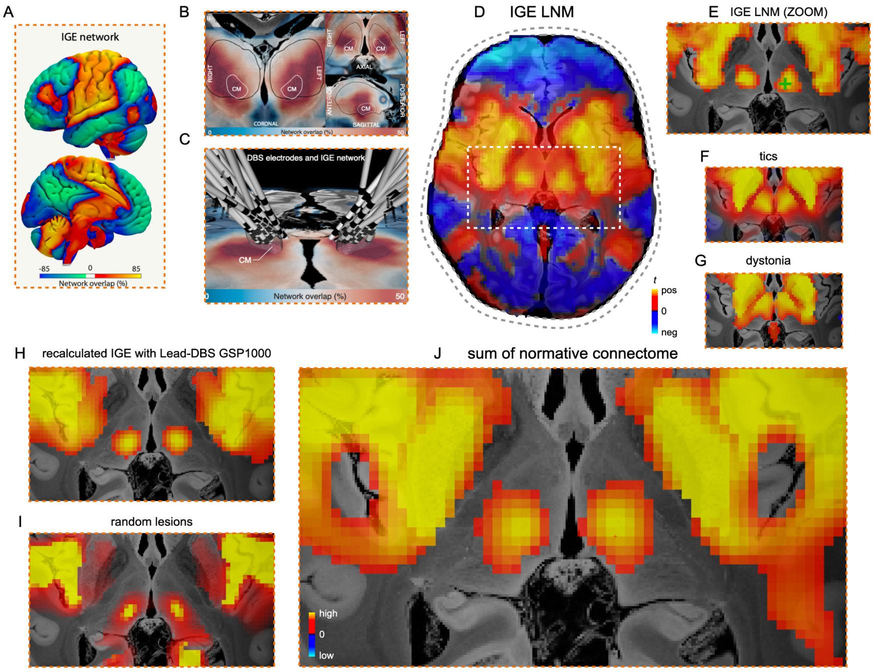

Supplementary Figure 8. Lesion Network Mapping proposed deep brain stimulation site central medial nucleus for idiopathic generalized epilepsy. Lesion Network Mapping (LNM) studies are also actively advocating the use of LNM derived stimulation sites for deep brain stimulation (DBS), for example, for the treatment of idiopathic generalized epilepsy (IGE) [8]. (A,B,C) Panels from the work by Ji et al. [8], showing the presented LNM network and stimulation sites of patients treated with DBS in the centromedian nucleus (CM) of the thalamus. Panels and data are described by Ji et al. in support of using the highest activated voxels in the LNM to place electrodes for DBS stimulation. (D) Panel shows the LNM map downloaded from the original paper [8] (2x2x2 mm), plotted on a 0.5x0.5x0.5 mm high-resolution T2 anatomical MRI image, as done in the paper by Ji et al. The LNM network recomputed by means of Lead-DBS and GSP1000 data is shown in panel H. (E) Panel shows a zoom of the white dotted box in D, with the highest voxel in the LNM proposed as stimulation site by Ji et al. voxel [−9.05, −21.07, −0.07] shown by the green +. (F-G). Panels show that a high signal in CM is not specific to IGE, with the same areas also showing high (and highest) voxels in LNM maps computed for lesions related to tics [83, 84] and cervical dystonia [85] (source of data Supplementary Table 2). (H) Panel shows the recalculated LNM map using Lead-DBS and GSP1000 data based on the coordinates reported by Ji et al., with CM again among the highest voxels in the LNM map. (I) Panel shows LNM map based on random region locations in the brain, again showing CM as one of the highest connected areas of the brain. This finding further confirms a lack of specificity for IGE. (J) Panel shows the sum of the voxel-wise connectivity matrix *C*, with CM voxels exhibiting the highest connectivity signal across all the brain, irrespective of lesion location or disease association. It further demonstrates that the peak voxels in the LNM map presented by Ji et al. for IGE, and proposed as a new DBS stimulation target, are non-specific and unrelated to IGE. CM, centromedian nucleus; DBS, deep brain stimulation; IGE, idiopathic generalized epilepsy; LNM, Lesion Network Mapping.

S17. Pseudocode for Lesion Network Mapping

Pseudocode for Lesion Network Mapping

Input:

- Preprocessed resting-state fMRI time-series of normative subjects $h = 1, \ldots, H$ (e.g., GSP_{1000}) for all brain voxels R.
- Patients $s = 1, \ldots, S$, each with a lesion location m_s, where each $m_s \subseteq \{1, \ldots, R\}$ lists the indices of a lesion.

for each patient $s = 1, \ldots, S$ **do**
  for each normative subject $h = 1, \ldots, H$ **do**
    Extract the average time-series of voxels $i \in m_s$.
    Compute the functional connectivity of the average time-series of voxels $i \in m_s$ with all other voxels in the brain.
    Apply Fisher r-to-z transformation to the functional connectivity values.
  end for
  Aggregate the Fisher-transformed connectivity values by performing a one-sample t-test across H normative subjects.
  Optionally threshold the t-statistic.
end for
Average the lesion t-maps across patients S.
Output:: LNM map

S18. Mathematical derivation of LNM

The methodological steps of Lesion Network Mapping (LNM) implemented in Lead-DBS (Horn et al. 2015) can also be obtained as a linear matrix multiplication:

$$\text{LNM} \propto \sum_{s=1}^{S}(M \times C),$$

with $C \in \mathbb{R}^{R\times R}$ representing the group-averaged functional connectivity matrix and $M \in [0,1]^{S\times R}$ the lesion matrix, where each row corresponds to the lesion vector $\vec{\mathbf{m}}_s$ of patient s. The lesion vector $\vec{\mathbf{m}}_s \in [0,1]^{1\times R}$ contains one entry for each of the R brain voxels and assigns a value of $1/|m_s|$ if voxel $i \in m_s$, where $|m_s|$ is the lesion size, and assigns zero to all other voxels.

This linear form can be obtained as follows:
In Step 1 of voxel-wise LNM, the lesion m_s of patient s is mapped to the matching voxels i in the standard space of subjects in the normative dataset H. Next, in Step 2, the average time-series $x^h(t)$ across the voxels $i \in m_s$ is computed

$$x_s^h(t) = \sum_{i=1}^{R} \mathbf{m}_{s,i}\, y_i^h(t),$$

where $\mathbf{m}_{s,i}$ represents the value in the lesion vector of patient s at voxel i and $y_i^h(t)$ is the time-series of normative subject h at voxel i. The functional connectivity $FC_{s,r}^h$ between the lesion $\vec{\mathbf{m}}_s$ of patient s and voxel r in normative subject h is calculated as:

$$FC_s^h = \text{corr}\big(x_s^h(t),\, y_r^h(t)\big) \quad \text{for all } r \in R,$$

where $x_s^h(t)$ is the average time-series across voxels $i \in m_s$ for patient s, in normative subject h, and R is the set of all voxels in the brain mask. The functional connectivity $FC_{s,r}^h$ of the lesion vector $\vec{\mathbf{m}}_s$ of patient s at voxel r can also be obtained by first pre-computing the functional connectivity matrix C^h of normative subject h

$$C^h = \text{corr}\big(y_i^h(t),\, y_r^h(t)\big) \quad \text{for all } i, r \in R,$$

and then averaging the correlation values C^h between voxels $i \in m_s$ and voxel r:

$$FC_{s,r}^h = \text{corr}\big(x_s^h(t),\, y_r^h(t)\big) \quad \approx \quad \sum_{i=1}^{R} \mathbf{m}_{s,i} \times \text{corr}\Big(y_i^h(t),\, y_r^h(t)\Big) = \sum_{i=1}^{R} \mathbf{m}_{s,i} \times C_{i,r}^h,$$

where $C_{i,r}^h$ is the functional connectivity between voxel i and voxel r in normative subject h.
The functional connectivity $FC_{s,r}^h$ value is next standardized using the Fisher r-to-z transformation:

$$\tilde{FC}_{s,r}^h = \text{arctanh}\big(FC_{s,r}^h\big) \quad \approx \quad \sum_{i=1}^{R} \mathbf{m}_{s,i} \times \tilde{C}_{i,r}^h,$$

where $\tilde{C}_{i,r}^h = \text{arctanh}\big(C_{i,r}^h\big)$.

Step 1 and Step 2 are repeated for all normative subjects $h \in H$, and the resulting $\tilde{FC}_s^h$ maps are combined with a one-sample t-test. Approximating the standard deviation across connections equal $\sigma \approx \sigma_r$ (the assumption was empirically validated, see Supplementary Information S8), the denominator in the t-statistic is equal across all voxels $i \in R$, and the t-map can be obtained by the mean of the Fisher r-to-z transformed functional connectivity values multiplied by the scale factor $\frac{\sqrt{H}}{\sigma}$:

$$t\text{-map}_r^s = \frac{\frac{1}{H}\sum_{h=1}^{H} \tilde{FC}_{s,r}^h}{\sigma_r/\sqrt{H}} \quad \approx \quad \frac{\sqrt{H}}{\sigma}\frac{1}{H}\sum_{i=1}^{R} \mathbf{m}_{s,i} \times \big(\tilde{C}_{i,r}^1 + \tilde{C}_{i,r}^2 + \cdots + \tilde{C}_{i,r}^H\big) = \frac{\sqrt{H}}{\sigma}\sum_{i=1}^{R} \mathbf{m}_{s,i} \times \tilde{C}_{i,r}.$$

In the group analysis of Step 3, the mean $\tilde{FC}$ of a single voxel r across patients $s \in S$ is calculated as:

$$\text{LNM}_r \propto \sum_{s=1}^{S}\sum_{i=1}^{R}(\mathbf{m}_{s,i} \times \tilde{C}_{i,r}),$$

where $\mathbf{m}_{s,i}$ is the value in the lesion vector $\vec{\mathbf{m}}_s$ of patient s at voxel i, and $\tilde{C}$ is the group average of the Fisher r-to-z transformed functional connectivity matrix across normative subjects $h \in H$.

We can define an intermediate matrix $Cm \in \mathbb{R}^{S\times R}$, where Cm represents the selection of rows from the group-averaged functional connectivity matrix C that match the set of lesion vectors $\vec{\mathbf{m}}_s$ across all patients $s \in S$:

$$Cm = \begin{bmatrix} \vec{\mathbf{m}}_1 \\ \vec{\mathbf{m}}_2 \\ \vdots \\ \vec{\mathbf{m}}_S \end{bmatrix} \times \tilde{C} = M \times \tilde{C},$$

where $M \in [0,1]^{S\times R}$ is the lesion matrix containing all $\vec{\mathbf{m}}_s$ as rows. The group-level LNM network is obtained by averaging over all patients $s \in S$:

$$\begin{aligned} \text{LNM} &\propto \sum_{s=1}^{S} Cm \\ &\propto \sum_{s=1}^{S} (M \times \tilde{C}). \end{aligned}$$

For correlation values within the range -0.5 $< C_{i,r} <$ 0.5 of the normative dataset GSP1000, the Fisher r-to-z transformation is approximately linear ($\tilde{C}_{i,r} \approx C_{i,r}$) and C can similarly be used to compute the group-level LNM network:

$$\text{LNM} \propto \sum_{s=1}^{S} (M \times C).$$

S19. Mathematical derivation of sLNM variant

The methodological steps of symptom Lesion Network Mapping (sLNM) can be obtained as a linear matrix multiplication:

$$\text{sLNM} \propto \vec{sv} \times (M \times C),$$

where $C \in \mathbb{R}^{R \times R}$ is the group-averaged functional connectivity matrix and $M \in [0,1]^{S \times R}$ is the lesion matrix, with rows corresponding to the lesion vector $\vec{\mathbf{m}}_s$ of patient s, and $\vec{sv} \in \mathbb{R}^{1 \times S}$ is a vector of the patient symptom scores.

This linear form can be obtained as follows:
Step 1 and Step 2 of both LNM and sLNM are the same, which can be derived (see Supplementary Information S18) as:

$$Cm \ = \ M \times C \ \in \mathbb{R}^{S \times R},$$

where Cm represents the selection of rows from the group-averaged functional connectivity matrix C that match the set of lesion vectors $\vec{\mathbf{m}}_s$ in M across all patients $s \in S$.

The group-level analysis in Step 3 of sLNM involves correlating the symptom scores for voxel j with the functional connectivity of the selected lesions (illustrated in Supplementary Figure 1). The Pearson correlation coefficient r between any two variables x and y is given by:

$$r = \frac{\sum_{i=1}^{n} \left(x_i - \bar{x}\right) \ \times \left(y_i - \bar{y}\right)}{\sqrt{\sum_{i=1}^{n} \left(x_i - \bar{x}\right)^2} \sqrt{\sum_{i=1}^{n} \left(y_i - \bar{y}\right)^2}},$$

where $\bar{x}$ and $\bar{y}$ are the means of x and y. Thus, for each voxel j, sLNM$_j$ is computed by the Pearson correlation coefficient between the j-th column of Cm and $\vec{sv}$:

$$\text{sLNM}_j = \frac{\sum_{s=1}^{S} \left(Cm_{s,j} - \bar{Cm}_j\right) \times \left(sv_s - \bar{sv}\right)}{\sqrt{\sum_{s=1}^{S} \left(Cm_{s,j} - \bar{Cm}_j\right)^2} \sqrt{\sum_{s=1}^{S} \left(sv_s - \bar{sv}\right)^2}},$$

where $\bar{Cm}_j$ is the mean functional connectivity of lesion $\vec{\mathbf{m}}_s$ across patients $s \in S$ at voxel j, and $\bar{sv}$ is the mean symptom score across patients $s \in S$.

When the standard deviation in Cm_j is approximated by the standard deviation C_j and as a constant across all voxels $j \in R$, sLNM$_j$ can be approximated by the numerator multiplied by a fixed scale factor $1/(S-1) \times \sigma_C \times \sigma_{sv}$, with S the number of patients, σ_C is the standard deviation of C, and σ_{sv} is the standard deviation of $\vec{sv}$, sLNM$_j$ can be expressed as:

$$\begin{aligned} \text{sLNM}_j &\propto \sum_{s=1}^{S} Cm_{s,j} \times (sv_s - \bar{sv}) \ - \ \bar{Cm}_j \times \sum_{s=1}^{S} (sv_s - \bar{sv}) \\ &\propto \sum_{s=1}^{S} Cm_{s,j} \times (sv_s - \bar{sv}). \end{aligned}$$

After substituting $Cm = M \times C$, and standardizing $\vec{sv}$ to ensure a zero mean, the sLNM network at voxel j can be expressed as:

$$\text{sLNM}_j \ \propto \sum_{s=1}^{S} \sum_{i=1}^{R} M_{si} \times C_{ij} \times sv_s.$$

Extending this to all voxels $j \in R$ the sLNM network takes the compact matrix form:

$$\text{sLNM} \propto \vec{sv} \times (M \times C).$$

S20. Code examples

Repository
The code and data repository can be downloaded from:

https://github.com/dutchconnectomelab/lesionnetworkmapping

The LNM code examples and data repository.
The LNM repository contains example code for LNM and sLNM, original published and extracted patient lesion masks, pre-computed Lead-DBS output, voxel-wise GSP1000 connectivity degree files, atlas-based connectivity matrices *C*, and published LNM and sLNM maps.

Tutorial code examples include:

Example_LNM_step123.m
- Performs individual (Step 1 & 2) and group analysis (Step 3) for voxel-wise LNM.
- Demonstrates the linear form: LNM = sum(M x C).
- Demonstrates that the two implementations lead to the same LNM network.
- Several example lesion sets are included.

Example_LNM_randomspinLesions.m
- Shows LNM overlap for a set of lesions.
- Demonstrates the quality of the LNM map when lesions are randomized across the brain using a spin-model.
- Several example lesion sets are included.

Example_LNM_specificity_randomLesions.m
- Compares the similarity of an LNM network for patient lesions of a specific disorder to LNM maps generated from random lesions.
- Several example lesion sets are included.

Example_sLNM_step123.m
- Demonstrates the 3-step symptom LNM (sLNM) procedure.
- Steps 1 & 2 are identical to Example1_LNM_step123.m.
- Step 3 correlates individual functional connectivity lesion maps with symptom scores vector sv: sLNM = sv x (M x C)
- Includes 4 example lesion sets

Example_sLNM_randomSv_randomLesions.m
- Shows that similar sLNM maps to published literature can be obtained using both random lesions and random 'sv' scores.
- Includes several published sLNM maps.

Example_regressionModel.m
- Demonstrates that the majority of variance in LNM and sLNM maps can be explained by structural properties of the connectivity matrix C.
- Includes several published LNM and sLNM maps for testing.

Supplementary Tables

Supplementary Table 1. Summary of studies discussing or using the Lesion Network Mapping framework identified through systematic review. Summary of studies identified through a systematic review of literature, discussing and/or studying LNM, methodologically equivalent methods under a different label (seventh column), and/or variants of the method, identifying in total 201 LNM publications between 2015 and 2025. Resulting papers were manually reviewed. Study eligibility was assessed through a two-stage screening process, including a systematic review of the Methods and Supplementary information and materials to verify the use of a normative connectivity dataset, network-based mapping of focal brain perturbations, and/or aggregation of resulting connectivity maps into a joint group map. When a code repository was available, it was used to confirm the method implementation; for studies without publicly available code, eligibility was determined based on the written text only. The last update of the literature overview was done December 2025. We note that this list is likely non-exhaustive, and despite systematic screening, some relevant studies may have been missed or misclassified; an up-to-date overview of the list is presented on the GitHub repository page (see Supplementary Note S20).

Identified studies span 101 distinct conditions, including neuropsychiatric, neurological, neurodegenerative disorders, and cognitive functions. Of 72 of these identified studies (Supplementary Table 2) lesion and/or LNM data could be extracted. A summary table of identified studies is given, together with the results of an additional search of NIH research projects and clinical trials (RePORTER and ClinicalTrials.gov), which identified 14 projects involving clinical applications, and 10 registered clinical studies (8 ongoing); number of treated conditions are listed. Full list of search terms and references given in Supplementary Note S1 and Supplementary Table 1. Full paper table includes year of publication (first column), article reference (second column), article title (third column), condition studied (fourth column), lesion type from which LNM was computed (fifth column; e.g., lesion locations, stimulation sites, or brain coordinates), method used to compute LNM (sixth column; categorized as standard LNM, symptom LNM [sLNM], contrast LNM where LNM maps are compared between sub-groups of patients [cLNM], or review/commentary), LNM nomenclature used by authors in original publication (seventh column; dash denotes no specific term specified), and reported lesion distribution classified as homogeneous or heterogeneous (eighth column). In the sLNM variant, networks are derived by correlating patient symptom scores with lesion-derived functional connectivity maps. In the cLNM variant, group-level maps are obtained by contrasting subgroups with different symptom severity. ADHD, attention-deficit/hyperactivity disorder; OCD, obsessive-compulsive disorder; PTSD, post-traumatic stress disorder; REM, rapid eye movement; TBI, traumatic brain injury. cLNM, contrast Lesion Network Mapping; LNM, Lesion Network Mapping; sLNM, symptom Lesion Network Mapping. *Table is presented as an online file.*

Table Summary *(Full table is presented below).*

**Conditions**	Depression/Anxiety (17), Stroke (11), Parkinson's disease (10), Cognition (9), Psychosis (9), Epilepsy (8), Cognitive impairment (7), Alzheimer's disease (5), Dystonia (5), OCD (4), Schizophrenia (4), Aphasia (3), Tourette syndrome/Tics (3), Tremor (3), ADHD (2), Alien limb (2), Anosognosia (2), Bipolar/Mania (2), Disorientation (2), Frontotemporal dementia (2), Multiple sclerosis (2), Neglect syndrome (2), Physiology (2), Psychopathy (2), REM sleep behavior disorder (2), Vertigo (2), Addiction (1), Agression (1), Alcohol use disorder remission (1), Alice in Wonderland Syndrome (1), Amnesia (1), Amusia (1), Aphantasia (1), Apnea (1), Apraxia (eye-opening) (1), Ataxia (1), Autism spectrum disorder (1), Autoscopic phenomena (1), Bipolar disorder (1), Blindsight (1), Cerebellar cognitive affective syndrome (1), Cerebral small vessel disease (1), Coma (1), Confabulation (1), Cortical dysplasia (1), Corticobasal syndrome (1), Creativity (1), Criminality (1), Delusional misidentifications (1), Delusions (1), Depression treatment (1), Dyskinetic cerebral palsy (1), Dysnomia (1), Emotion regulation (1), Executive function (1), Fall risk (1), Foreign accent syndrome (1), Freezing gait (1), Functional and somatic symptoms (1), Fundamentalism (1), Gait (1), Head mislocalization (1), Hemichorea-hemiballismus (1), Holmes tremor (1), Hyperkinetic seizures (1), Loss consciousness (1), Migraine

	(1), Mind-wandering (1), Mutism (1), Neonatal arterial ischemic stroke (1), Neurogenic stuttering (1), Oculogyric crises (1), PTSD (1), Paramnesia (1), Pathological urge (1), Pedophilia (1), Peduncular hallucinosis (1), Persisting symptoms after concussion (1), Personality (1), Political involvement (1), Post-stroke depression (1), Post-stroke spasticity (1), Progressive supranuclear palsy (1), Prosopagnosia (1), REM sleep (1), Religiosity (1), Resistance training (1), Seizures (1), Sensory symptoms (1), Spasms (1), Step length asymmetry (1), Substance abuse disorder (1), Suicide (1), Survival glioblastoma (1), TBI (1), Time orientation (1), Transdiagnostic (1), Transient global amnesia (1), Vestibular graviception (1), Visuospatial neglect (1), Other (15). *Total = 201.*
**Pre-registered projects**	Depression/Anxiety (6), OCD (2), Bipolar disorder (1), Schizophrenia (1), Autism spectrum disorder (1), Epilepsy (1), Addiction (1), Psychosis (1), Stroke (1), Parkinson's disease (1), Alzheimer's disease (1). *Total = 14.*
**Clinical trials**	Depression/Anxiety (5), OCD (3), Schizophrenia (1), Psychosis (1), PTSD (1), Bipolar disorder (1), Parkinson's disease (1), Stroke (1). *Total = 10.*

Table S1

Year	Reference	Article	Condition	Lesion type	LNM type	Nomenclature	Reported lesion distribution
2015	Brain. 2015 Oct;138(Pt 10):3061-75. doi: 10.1093/brain/awv228	Network localization of neurological symptoms from focal brain lesions	Peduncular hallucinosis	Lesions	LNM	Lesion network mapping	Heterogeneous
2016	Neurology. 2016 Dec 6;87(23):2427-2434. doi: 10.1212/WNL.0000000000003404	A human brain network derived from coma-causing brainstem lesions	Coma	Lesions	LNM	-	Homogeneous
2016	Cortex. 2016 May;78:31-43. doi: 10.1016/j.cortex.2016.02.002	Canceled connections: lesion-derived network mapping helps explain differences in performance on a complex decision-making task	Cognition	Lesions	LNM	Lesion-derived network mapping	Heterogeneous
2016	Neurology. 2016 Jun 7;86(23):2187-95. doi: 10.1212/WNL.0000000000002741	Network localization of hemichorea-hemiballismus	Hemichorea-hemiballismus	Lesions	LNM	Lesion network mapping	Heterogeneous
2017	Ann Neurol. 2017 Jul;82(1):67-78. doi: 10.1002/ana.24974	Connectivity predicts deep brain stimulation outcome in Parkinson disease	Parkinson's disease	Stimulation sites	sLNM	Connectivity estimation	Not applicable
2017	Brain. 2017 Feb;140(2):497-507. doi: 10.1093/brain/aww288	Finding the imposter: brain connectivity of lesions causing delusional misidentifications	Delusional misidentifications	Lesions	LNM	Lesion network mapping	Not mentioned
2017	Ann Neurol. 2017 Jan;81(1):129-141. doi: 10.1002/ana.24845	Lesions causing freezing of gait localize to a cerebellar functional network	Freezing gait	Lesions	LNM	Lesion network mapping	Heterogeneous
2018	Ann Neurol. 2018 Jul;84(1):153-157. doi: 10.1002/ana.25285	Identifying therapeutic targets from spontaneous beneficial brain lesions	Tremor	Lesions	LNM	Lesion network mapping	Heterogeneous
2018	Proc Natl Acad Sci U S A. 2018 Jan 16;115(3):601-606. doi: 10.1073/pnas.1706587115	Lesion network localization of criminal behavior	Criminality	Lesions	LNM	Lesion network mapping	Heterogeneous
2018	Proc Natl Acad Sci U S A. 2018 Oct 16;115(42):10792-10797. doi: 10.1073/pnas.1814117115	Lesion network localization of free will	Alien limb, Mutism	Lesions	LNM	Lesion network mapping, Lesion network localization	Heterogeneous
2018	Brain. 2018 Aug 1;141(8):2445-2456. doi: 10.1093/brain/awy161	Localizing parkinsonism based on focal brain lesions	Parkinson's disease	Lesions	LNM	Lesion network mapping	Heterogeneous
2018	N Engl J Med. 2018 Dec 6;379(23):2237-2245. doi: 10.1056/NEJMra1706158	Mapping symptoms to brain networks with the human connectome	Not applicable	Not applicable	Review	Lesion network mapping	Not applicable
2018	Neuroimage. 2018 Feb 1;166:317-324. doi: 10.1016/j.neuroimage.2017.11.011	The neuronal network involved in self-attribution of an artificial hand: a lesion network-symptom-mapping study	Cognition	Lesions	cLNM	Lesion network symptom mapping	Heterogeneous
2019	Biol Psychiatry. 2019 Nov 15;86(10):749-758. doi: 10.1016/j.biopsych.2019.07.023	A human depression circuit derived from focal brain lesions	Depression	Lesions	cLNM	Lesion network mapping	Heterogeneous
2019	Nat Commun. 2019 Aug 2;10(1):3497. doi: 10.1038/s41467-019-11353-z.	A human memory circuit derived from brain lesions causing amnesia	Amnesia	Lesions	LNM	Lesion network mapping	Heterogeneous
2019	Brain. 2019 Dec 1;142(12):3975-3990. doi: 10.1093/brain/awz332.	Looking beyond the face area: lesion network mapping of prosopagnosia	Prosopagnosia	Lesions	LNM	Lesion network mapping	Heterogeneous
2019	Ann Neurol. 2019 Dec;86(6):812-820. doi: 10.1002/ana.25618	Mapping holmes tremor circuit using the human brain connectome	Holmes tremor	Lesions	LNM	Lesion network mapping	Heterogeneous
2019	Brain. 2019 Jun 1;142(6):1660-1674. doi: 10.1093/brain/awz112	Network localization of cervical dystonia based on causal brain lesions	Dystonia	Lesions	LNM	Lesion network mapping	Heterogeneous
2019	Brain. 2019 Jan 1;142(1):70-79. doi: 10.1093/brain/awy292	Network localization of heterogeneous neuroimaging findings	Alzheimer's disease, Frontotemporal dementia, Corticobasal syndrome, Aphasia	Lesions	LNM	Lesion network mapping, Network localization meta-analysis	Heterogeneous
2019	Brain Commun. 2019;1(1):fcz006. doi: 10.1093/braincomms/fcz006	Neuroimaging in Parkinson's disease dementia: connecting the dots	Parkinson's disease	Lesions	LNM	Coordinate-based network mapping	Heterogeneous
2019	Neurology. 2019 Oct 15;93(16):e1561-e1571. doi: 10.1212/WNL.0000000000008326	Pediatric postoperative cerebellar cognitive affective syndrome follows outflow pathway lesions	Cerebellar cognitive affective syndrome	Lesions	LNM	Lesion network mapping	Homogeneous
2019	Brain. 2019 Aug 1;142(8):2198-2206. doi: 10.1093/brain/awz152	Subthalamic neuromodulation improves short-term motor learning in parkinson's disease	Parkinson's disease	Stimulation sites	LNM	-	Not applicable
2019	Brain. 2019 Aug 1;142(8):2207-2214. doi: 10.1093/brain/awz179	The antero-dorsal precuneal cortex supports specific aspects of bodily awareness	Cognition	Lesions	cLNM	Lesion network mapping	Homogeneous
2020	Hum Brain Mapp. 2020 Apr 15;41(6):1520-1531. doi: 10.1002/hbm.24892	Cortical lesions causing loss of consciousness are anticorrelated with the dorsal brainstem	Loss consciousness	Lesions	LNM	Lesion network mapping	Heterogeneous
2020	Am J Psychiatry. 2020 May 1;177(5):435-446. doi: 10.1176/appi.ajp.2019.19090915	Distinct symptom-specific treatment targets for circuit-based neuromodulation	Anxiety-depression symptoms	Stimulation sites	sLNM	-	Not applicable
2020	Neuroimage. 2020 Mar;208:116485. doi: 10.1016/j.neuroimage.2019.116485	Hippocampal diaschisis contributes to anosognosia for hemiplegia: evidence from lesion network-symptom-mapping	Anosognosia	Lesions	cLNM	Lesion network symptom mapping	Not mentioned
2020	Sci Rep. 2020 Jul 3;10(1):10989. doi: 10.1038/s41598-020-67626-x	Identification of neural networks preferentially engaged by epileptogenic mass lesions through lesion network mapping analysis	Epilepsy	Lesions	LNM	Lesion network mapping	Heterogeneous
2020	World Neurosurg. 2020 Jan;133:278-282. doi: 10.1016/j.wneu.2019.10.020	Lesion network mapping analysis identifies potential cause of postoperative depression in a case of cingulate low-grade glioma	Depression	Lesions	LNM	Connectomic mapping	Not applicable
2020	J Clin Invest. 2020 Oct 1;130(10):5209-5222. doi: 10.1172/JCI136096	Mapping mania symptoms based on focal brain damage	Mania	Lesions	cLNM	Lesion network mapping	Heterogeneous
2020	Brain. 2020 Feb 1;143(2):541-553. doi: 10.1093/brain/awz405	Mapping migraine to a common brain network	Migraine	Lesions	LNM	Coordinate-based network mapping	Heterogeneous
2020	Ann Neurol. 2020 Dec;88(6):1118-1131. doi: 10.1002/ana.25901	Network localization of alien limb in patients with corticobasal syndrome	Alien limb	Lesions	LNM	Atrophy network mapping	Heterogeneous
2020	Brain. 2020 Apr 1;143(4):1249-1260. doi: 10.1093/brain/awaa058	Network localization of clinical, cognitive, and neuropsychiatric symptoms in Alzheimer's disease	Alzheimer's disease	Coordinates	LNM	Atrophy network mapping	Heterogeneous
2020	J Cogn Neurosci. 2020 Dec;32(12):2303-2319. doi: 10.1162/jocn_a_01628	Network localization of executive function deficits in patients with focal thalamic lesions	Executive function	Lesions	LNM	Lesion network mapping	Not mentioned
2020	Brain. 2020 Jul 1;143(7):2173-2188. doi: 10.1093/brain/awaa156	Post-stroke deficit prediction from lesion and indirect structural and functional disconnection	Stroke	Lesions	sLNM	Functional disconnection map	Not mentioned
2020	Hum Brain Mapp. 2020 Oct 1;41(14):3984-3992. doi: 10.1002/hbm.25102	Rapid eye movement sleep patterns of brain activation and deactivation occur within unique functional networks	Rapid eye movement sleep	Lesions	LNM	-	Homogeneous
2020	Brain. 2021 Feb 12;144(1):e6. doi: 10.1093/brain/awaa351	Reply: Lesion network mapping: where do we go from here?	Not applicable	Not applicable	Commentary	Lesion network mapping	Not applicable
2020	Brain. 2020 May 1;143(5):e41. doi: 10.1093/brain/awaa095	Reply: The influence of sample size and arbitrary statistical thresholds in lesion-network mapping	Not applicable	Not applicable	Commentary	Lesion network mapping	Not applicable
2020	Brain. 2020 May 1;143(5):e40. doi: 10.1093/brain/awaa094	The influence of sample size and arbitrary statistical thresholds in lesion-network mapping	Not applicable	Not applicable	Commentary	Lesion network mapping	Not applicable
2021	Brain Commun. 2021 Nov 13;3(4):fcab259. doi: 10.1093/braincomms/fcab259	A novel stroke lesion network mapping approach: improved accuracy yet still low deficit prediction	Stroke	Lesions	LNM	Functional disconnection mapping	Not mentioned
2021	Biol Psychiatry. 2021 Nov 15;90(10):701-713. doi: 10.1016/j.biopsych.2021.04.006	A unified functional network target for deep brain stimulation in obsessive-compulsive disorder	Obsessive-compulsive disorder	Stimulation sites	sLNM	-	Not applicable
2021	Nat Hum Behav. 2021 Dec;5(12):1707-1716. doi: 10.1038/s41562-021-01161-1	Brain stimulation and brain lesions converge on common causal circuits in neuropsychiatric disease	Depression	Coordinates, Stimulation sites	sLNM	Causal mapping	Not mentioned
2021	Alzheimers Dement. 2021 May;17(5):777-787. doi: 10.1002/alz.12238	Brain structures and networks responsible for stimulation-induced memory flashbacks during forniceal deep brain stimulation for alzheimer's disease	Alzheimer's disease	Stimulation sites	sLNM	Connectomic mapping	Not applicable
2021	J Neurosurg Pediatr. 2021 Aug 27;28(5):600-608. doi: 10.3171/2021.5.PEDS20990	Categorizing cortical dysplasia lesions for surgical outcome using network functional connectivity	Cortical dysplasia	Lesions	cLNM	Lesion network mapping	Heterogeneous
2021	Neuroimage Clin. 2021;30:102612. doi: 10.1016/j.nicl.2021.102612	Common and distinct brain networks of autoscopic phenomena	Autoscopic phenomena	Lesions	LNM	Lesion network mapping	Heterogeneous
2021	Am J Psychiatry. 2021 Dec;178(12):1119-1128. doi: 10.1176/appi.ajp.2021.21010088	Coordinate-based network mapping of brain structure in major depressive disorder in younger and older adults: a systematic review and meta-analysis	Depression	Coordinates	LNM	Coordinate-based network mapping	Not mentioned
2021	Brain Imaging Behav. 2021 Oct;15(5):2681-2692. doi: 10.1007/s11682-020-00442-z	Idiopathic and acquired pedophilia as two distinct disorders: an insight from neuroimaging	Pedophilia	Lesions	LNM	Lesion network mapping	Heterogeneous
2021	J Neurosci Res. 2021 Jan;99(1):361-373. doi: 10.1002/jnr.24648	Lesion network mapping demonstrates that mind-wandering is associated with the default mode network	Mind-wandering	Lesions	sLNM	Lesion network mapping	Not mentioned
2021	Brain. 2021 May 7;144(4):e35. doi: 10.1093/brain/awab002	Lesion network mapping predicts post-stroke behavioural deficits and improves localization	Cognitive impairment	Lesions	LNM	Lesion network mapping	Not mentioned
2021	NeuroRehabilitation. 2021;48(1):133-138. doi: 10.3233/NRE-201555	Lesion-based structural and functional networks in patients with step length asymmetry after stroke	Step length asymmetry	Lesions	LNM	Lesion network mapping	Not mentioned
2021	Mol Psychiatry. 2021 Apr;26(4):1299-1309. doi: 10.1038/s41380-019-0565-3	Lesions causing hallucinations localize to one common brain network	Psychosis	Lesions	LNM	Lesion network mapping	Heterogeneous
2021	Brain Stimul. 2021 Mar-Apr;14(2):273-276. doi: 10.1016/j.brs.2021.01.009	Lesions causing self-injurious behavior engage putative networks modulated by deep brain stimulation	Depression	Lesions	LNM	Lesion network mapping	Heterogeneous
2021	Neuroimage Clin. 2021;31:102760. doi: 10.1016/j.nicl.2021.102760	Neural mechanisms of foreign accent syndrome: lesion and network analysis	Foreign accent syndrome	Lesions	LNM	Lesion network mapping	Heterogeneous
2021	Neuroimage. 2021 Aug 1;236:118089. doi: 10.1016/j.neuroimage.2021.118089	Painting by lesions: white matter hyperintensities disrupt functional networks and global cognition	Cognitive impairment	Lesions	LNM	Lesion network mapping	Not mentioned
2021	Brain. 2021 Dec 16;144(11):3529-3540. doi: 10.1093/brain/awab232	Potential optimization of focused ultrasound capsulotomy for obsessive compulsive disorder	Obsessive-compulsive disorder	Stimulation sites	sLNM	Lesion network mapping	Not applicable
2021	Brain. 2021 Feb 12;144(1):44-52. doi: 10.1093/brain/awaa341	Subthalamic stimulation impairs stopping of ongoing movements	Parkinson's disease	Stimulation sites	sLNM	-	Not applicable
2021	Ann Neurol. 2021 Apr;89(4):726-739. doi: 10.1002/ana.26015	Tuber locations associated with infantile spasms map to a common brain network	Spasms	Lesions	LNM	Lesion network mapping	Heterogeneous
2021	Ann Neurol. 2021 Jun;89(6):1181-1194. doi: 10.1002/ana.26079	Unravelling the neural basis of spatial delusions after stroke	Delusions	Lesions	LNM	Lesion network mapping	Not mentioned
2022	Brain. 2022 May 24;145(4):1410-1421. doi: 10.1093/brain/awac012	A brain network for deep brain stimulation induced cognitive decline in Parkinson's disease	Parkinson's disease	Stimulation sites	LNM	-	Not applicable
2022	Brain Struct Funct. 2022 Nov;227(8):2787-2795. doi: 10.1007/s00429-022-02573-z	A depression network caused by brain tumours	Depression	Lesions	LNM	Lesion network mapping	Heterogeneous
2022	Biol Psychiatry. 2022 Feb 15;91(4):380-388. doi: 10.1016/j.biopsych.2021.06.016	A neural circuit for spirituality and religiosity derived from patients with brain lesions	Religiosity	Lesions	sLNM	Lesion network mapping	Heterogeneous
2022	Brain. 2022 Dec 19;145(12):4385-4397. doi: 10.1093/brain/awac009	A neural network for tics: insights from causal brain lesions and deep brain stimulation	Tics	Lesions, Stimulation sites	LNM	Lesion network mapping	Heterogeneous
2022	Nat Hum Behav. 2022 Oct;6(10):1417-1429. doi: 10.1038/s41562-022-01371-1	Activation network mapping for integration of heterogeneous fMRI findings	Cognition	Coordinates	LNM	Activation network mapping	Heterogeneous
2022	Nat Med. 2022 Jun;28(6):1249-1255. doi: 10.1038/s41591-022-01834-y	Brain lesions disrupting addiction map to a common human brain circuit	Addiction	Lesions	cLNM	Lesion network mapping	Heterogeneous
2022	Nat Rev Neurosci. 2022 Jun;23(6):361-375. doi: 10.1038/s41583-022-00583-8	Causal mapping of human brain function	Not applicable	Not applicable	Review	Lesion network mapping, Causal brain mapping	Not applicable
2022	Brain Struct Funct. 2022 Dec;227(9):3099-3108. doi: 10.1007/s00429-022-02556-0	Central precuneus lesions are associated with impaired executive function	Cognitive impairment	Lesions	LNM	Lesion network mapping	Not mentioned
2022	Brain Struct Funct. 2022 Dec;227(9):3063-3073. doi: 10.1007/s00429-022-02530-w	Evaluating the rare cases of cortical vertigo using disconnectome mapping	Vertigo	Lesions	LNM	Lesion network mapping, Disconnectome mapping	Homogeneous
2022	Neurology. 2023 Jan 31;100(5):e485-e496. doi: 10.1212/WNL.0000000000201488	Involvement of thalamocortical networks in patients with poststroke thalamic aphasia	Aphasia	Lesions	cLNM	Lesion network symptom mapping	Not mentioned
2022	Curr Opin Neurol. 2022 Aug 1;35(4):453-459. doi: 10.1097/WCO.0000000000001085	Lesion network mapping for symptom localization: recent developments and future directions	Not applicable	Not applicable	Review	Lesion network mapping	Not applicable
2022	Acta Neurochir (Wien). 2022 Dec;164(12):3285-3289. doi: 10.1007/s00701-022-05355-y	Lesion network mapping of ectopic craniopharyngioma identifies potential cause of psychosis: a case report	Psychosis	Lesions	LNM	Lesion network mapping	Not applicable
2022	Brain Struct Funct. 2022 Dec;227(9):3121-3127. doi: 10.1007/s00429-022-02508-8	Lesion network mapping of mania using different normative connectomes	Mania	Lesions	LNM	Lesion network mapping	Not applicable
2022	Front Neuroanat. 2022 Sep 29;16:819412. doi: 10.3389/fnana.2022.819412	Lesions causing central sleep apnea localize to one common brain network	Apnea	Lesions	LNM	Lesion network mapping	Heterogeneous
2022	Front Aging Neurosci. 2022 Oct 25;14:1011812. doi: 10.3389/fnagi.2022.1011812	Lesions causing post-stroke spasticity localize to a common brain network	Post-stroke spasticity	Lesions	LNM	Lesion network mapping	Heterogeneous
2022	Neurobiol Aging. 2022 Jan;109:166-175. doi: 10.1016/j.neurobiolaging.2021.09.023	Mind the gaps: functional networks disrupted by white matter hyperintensities are associated with greater falls risk	Fall risk	Lesions	LNM	Lesion network mapping	Not mentioned
2022	Sci Rep. 2022 Dec 27;12(1):22400. doi: 10.1038/s41598-022-26945-x	Multimodal and multidomain lesion network mapping enhances prediction of sensorimotor behavior in stroke patients	Stroke	Lesions	sLNM	Lesion network mapping	Not mentioned
2022	Ann Neurol. 2022 Nov;92(5):834-845. doi: 10.1002/ana.26468	Network effects of brain lesions causing central poststroke pain	Stroke	Lesions	cLNM	Lesion network mapping	Not mentioned
2022	Ann Neurol. 2022 Feb;91(2):217-224. doi: 10.1002/ana.26292	Network localization of unconscious visual perception in blindsight	Blindsight	Lesions	cLNM	Lesion network mapping	Not mentioned
2022	Clin Psychol Rev. 2022 Aug;96:102189. doi: 10.1016/j.cpr.2022.102189	Neural substrates of motivational dysfunction across neuropsychiatric conditions: evidence from meta-analysis and lesion network mapping	Anhedonia/Apathy	Lesions	LNM	Lesion network mapping	Heterogeneous
2022	Proc Natl Acad Sci U S A. 2022 Apr 5;119(14):e2114985119. doi: 10.1073/pnas.2114985119	Optimal deep brain stimulation sites and networks for cervical vs. generalized dystonia	Dystonia	Stimulation sites	sLNM	DBS network mapping	Not applicable
2022	Nat Commun. 2022 Dec 14;13(1):7707. doi: 10.1038/s41467-022-34510-3	Optimal deep brain stimulation sites and networks for stimulation of the fornix in alzheimer's disease	Alzheimer's disease	Stimulation sites	LNM	DBS network mapping	Not applicable
2022	Brain. 2022 May 24;145(4):1338-1353. doi: 10.1093/brain/awac010	Post-stroke outcomes predicted from multivariate lesion-behaviour and lesion network mapping	Stroke	Lesions	LNM	Lesion network mapping	Not mentioned
2022	Neuroimage. 2022 May 1;251:119001. doi: 10.1016/j.neuroimage.2022.119001	Resolution of diaschisis contributes to early recovery from post-stroke aphasia	Stroke	Lesions	LNM	Lesion network mapping	Not mentioned
2022	Brain. 2022 Mar 29;145(1):251-262. doi: 10.1093/brain/awab258	Subthalamic and pallidal deep brain stimulation: are we modulating the same network?	Parkinson's disease	Stimulation sites	sLNM	DBS network mapping	Not applicable
2022	medRxiv [Preprint]. 2022 Mar 03.22.22272566. doi: 10.1101/2022.03.22.22272566	The brain mechanisms of self-identification & self-location in neurosurgical patients using virtual reality and lesion network mapping	Cognition	Lesions	LNM	Lesion network mapping	Not mentioned
2022	J Neurodev Disord. 2022 Mar 12;14(1):19. doi: 10.1186/s11689-022-09433-1	Using causal methods to map symptoms to brain circuits in neurodevelopment disorders: moving from identifying correlates to developing treatments	Not applicable	Not applicable	Review	Lesion network mapping	Not applicable
2022	J Alzheimers Dis. 2022;90(2):553-563. doi: 10.3233/JAD-220142	Weight for it: resistance training mitigates white matter hyperintensity-related disruption to functional networks in older females	Resistance training	Lesions	LNM	Lesion network mapping	Not mentioned
2023	Biol Psychiatry. 2023 Oct 15;94(8):640-649. doi: 10.1016/j.biopsych.2023.02.007	A lesion-derived brain network for emotion regulation	Emotion regulation	Lesions	LNM	Lesion network mapping	Not mentioned
2023	Asian J Psychiatr. 2023 Oct;88:103750. doi: 10.1016/j.ajp.2023.103750	A pilot study to investigate the efficacy and tolerability of lesion network guided transcranial electrical stimulation in outpatients with psychosis spectrum illness	Psychosis	Stimulation sites	LNM	Lesion network mapping	Not applicable
2023	Nat Hum Behav. 2023 Mar;7(3):420-429. doi: 10.1038/s41562-022-01501-9	A transdiagnostic network for psychiatric illness derived from atrophy and lesions	Transdiagnostic	Lesions, Coordinates	LNM	Coordinate network mapping	Heterogeneous
2023	Brain Commun. 2023 Mar 17;5(2):fcad071. doi: 10.1093/braincomms/fcad071	A vertigo network derived from human brain lesions and brain stimulation	Vertigo	Lesions	LNM	Lesion network mapping	Heterogeneous
2023	Nat Ment Health. 2023 Mar;1(3):174-185. doi: 10.1038/s44220-023-00038-8	Altered brain activity in unipolar depression unveiled using connectomics	Depression	Lesions	LNM	Network-based meta-analytic approaches	Heterogeneous
2023	Front Neurol. 2023 May 17;14:1144228. doi: 10.3389/fneur.2023.1144228	Associations of lesion location, structural disconnection, and functional diaschisis with depressive symptoms post stroke	Depression	Lesions	sLNM	Lesion network symptom mapping	Not mentioned
2023	Brain Struct Funct. 2023 Dec;228(9):2067-2087. doi: 10.1007/s00429-023-02702-2	Comprehensive voxel-wise, tract-based, and network lesion mapping reveals unique architectures of right and left visuospatial neglect	Visuospatial neglect	Lesions	sLNM	Network-based lesion mapping	Not mentioned
2023	medRxiv [Preprint]. 2023 Apr 3:2023.03.31.23287980. doi: 10.1101/2023.03.31.23287980	Efficacy and tolerability of lesion network guided transcranial electrical stimulation in outpatients with psychosis spectrum illness: a nonrandomized controlled trial	Psychosis	Stimulation sites	LNM	Lesion network mapping	Not applicable
2023	Front Neurol. 2023 Jun 30;14:1100067. doi: 10.3389/fneur.2023.1100067	Functional and structural lesion network mapping in neurological and psychiatric disorders: a systematic review	Not applicable	Not applicable	Review	Lesion network mapping	Not applicable
2023	Stroke. 2023 Nov;54(11):2918-2922. doi: 10.1161/STROKEAHA.123.044470	Functional lesion network mapping of sensory deficits after ischemic stroke	Cognitive impairment	Lesions	cLNM	Lesion network mapping	Not mentioned
2023	Nat Ment Health 2023 Oct;1(10):772-81. doi: 10.1038/s44220-023-00128-7	Heterogeneous neuroimaging findings across substance use disorders localize to a common brain network	Substance abuse disorder	Lesions	LNM	Coordinate network mapping	Heterogeneous
2023	Neuroimage. 2023 Aug 15;277:120243. doi: 10.1016/j.neuroimage.2023.120243	Human anterior thalamic stimulation evoked cortical potentials align with intrinsic functional connectivity	Physiology	Stimulation sites	LNM	-	Not applicable
2023	Sci Rep. 2023 Mar 11;13(1):4052. doi: 10.1038/s41598-022-21905-x	Individualized precision targeting of dorsal attention and default mode networks with rtms in traumatic brain injury-associated depression	Traumatic brain injury	Stimulation sites	LNM	-	Not applicable
2023	Brain. 2023 Apr 19;146(4):1672-1685. doi: 10.1093/brain/awac361	Large-scale lesion symptom mapping of depression identifies brain regions for risk and resilience	Depression	Lesions	LNM	Lesion network mapping	Not mentioned
2023	bioRxiv [Preprint]. 2023 Jun 7:2023-06. doi: 10.1101/2023.06.05.543261	Lesion network localization of a stable personality trait.	Personality	Lesions	LNM	Lesion network mapping, Lesion network localization	Heterogeneous
2023	Nat Ment Health. 2023 Jan;1(3):36-44. doi: 10.1038/s44220-022-00002-y	Lesion network localization of depression in multiple sclerosis	Multiple sclerosis (depression)	Lesions	sLNM	Lesion network mapping, Lesion network localization	Not mentioned
2023	Ann Neurol. 2023 Sep;94(3):572-584. doi: 10.1002/ana.26721	Lesion network mapping for neurological deficit in acute ischemic stroke	Stroke	Lesions	sLNM	Functional disconnection mapping, Lesion network mapping	Not mentioned
2023	Brain Commun. 2023 Oct 27;5(6):fcad288. doi: 10.1093/braincomms/fcad288	Lesion network mapping of eye-opening apraxia	Apraxia (eye-opening)	Lesions	LNM	Lesion network mapping	Heterogeneous
2023	Ann Neurol. 2023 Sep;94(3):421-433. doi: 10.1002/ana.26681	Localization of a medial temporal lobe-precuneus network for time orientation	Time orientation	Lesions	LNM	Lesion network mapping	Not mentioned
2023	Brain Commun. 2023 Apr 4;5(3):fcad105. doi: 10.1093/braincomms/fcad105	Mapping a network for tics in tourette syndrome using causal lesions and structural alterations	Tics (Tourette syndrome)	Lesions	LNM	Lesion network mapping, Coordinate-based network mapping	Homogeneous
2023	Neurology. 2023 Oct 10;101(15):e1483-e1494. doi: 10.1212/WNL.0000000000207701	Mapping essential tremor to a common brain network using functional connectivity analysis	Tremor	Coordinates	LNM	Lesion network mapping, Coordinate network mapping	Heterogeneous
2023	JAMA Neurol. 2023 Sep 1;80(9):891-902. doi: 10.1001/jamaneurol.2023.1988	Mapping lesion-related epilepsy to a human brain network	Epilepsy	Lesions	cLNM	Lesion network mapping	Heterogeneous
2023	J Neurol. 2023 Nov;270(11):5211-5222. doi: 10.1007/s00415-023-11907-8	Multiple sclerosis lesions that impair memory map to a connected memory circuit	Multiple sclerosis (memory)	Lesions	sLNM	Lesion network mapping	Not mentioned
2023	J Neurol. 2023 Dec;270(12):6207-6209. doi: 10.1007/s00415-023-12059-5	Navigating the network: localising the lesion with the advent of lesion network mapping	Not applicable	Not applicable	Commentary	Lesion network mapping	Not applicable
2023	Ann Neurol. 2023 Sep;94(3):434-441. doi: 10.1002/ana.26709	Network localization of awareness in visual and motor anosognosia	Anosognosia	Lesions	LNM	Lesion network mapping	Not mentioned
2023	Neurol Sci. 2023 Feb;44(2):649-657. doi: 10.1007/s10072-022-06439-4	Network localization of transient global amnesia beyond the hippocampus	Transient global amnesia	Lesions	LNM	Lesion network mapping	Homogeneous
2023	Brain Res. 2023 Dec 1;1820:148559. doi: 10.1016/j.brainres.2023.148559	Neuroanatomical correlates of cognitive impairment following basal ganglia-thalamic post-hemorrhagic stroke: uncovering network-wide alterations in hemispheric gray matter asymmetry	Cognitive impairment	Lesions	LNM	Lesion network mapping	Homogeneous
2023	Korean J Radiol. 2023 Mar;24(3):247-258. doi: 10.3348/kjr.2022.0712	Neuroanatomical localization of rapid eye movement sleep behavior disorder in human brain using lesion network mapping	Rapid eye movement sleep behavior disorder	Lesions	LNM	Lesion network mapping	Heterogeneous
2023	Nat Neurosci. 2023 Sep;26(9):1613-1629. doi: 10.1038/s41593-023-01404-6	Regional, circuit and network heterogeneity of brain abnormalities in psychiatric disorders	Depression, Schizophrenia, Obsessive-compulsive disorder, Attention-deficit/ hyperactivity disorder, Autism spectrum disorder, Bipolar disorder	Coordinates	LNM	Lesion network mapping	Heterogeneous
2023	Biol Psychiatry. 2024 Mar 15;95(6):502-509. doi: 10.1016/j.biopsych.2023.11.011	Targeting symptom-specific networks with transcranial magnetic stimulation	Not applicable	Not applicable	Review	Causal brain mapping, Causal target mapping	Not applicable
2023	Cortex. 2023 Oct;167:12-24. doi: 10.1016/j.cortex.2023.06.006	The neuropsychology and neuroanatomy of reduplicative paramnesia	Paramnesia	Lesions	LNM	Lesion network mapping	Homogeneous
2023	Brain. 2023 Aug 1;146(8):3146-3155. doi: 10.1093/brain/awad123	The return of the lesion for localization and therapy	Not applicable	Not applicable	Review	Lesion network mapping	Not applicable
2023	J Neurosci Res. 2023 Feb;101(2):245-255. doi: 10.1002/jnr.25142	Unruptured brain arteriovenous malformations causing seizures localize to one common brain network	Seizures	Lesions	LNM	Lesion network mapping	Heterogeneous
2023	Nat Commun. 2023 Mar 29;14(1):1740. doi: 10.1038/s41467-023-37330-1	White matter disconnection of left multiple demand network is associated with post-lesion deficits in cognitive control	Cognition	Lesions	LNM	Functional connectivity disconnection scores	Not mentioned
2024	Nat Commun. 2024 Dec 16;15(1):10687. doi: 10.1038/s41467-024-55242-6	A critical role of action-related functional networks in gilles de la tourette syndrome	Tourette syndrome	Stimulation sites	LNM	Lesion network mapping	Not applicable
2024	Cereb Cortex. 2024 Jan 14;34(1):bhad486. doi: 10.1093/cercor/bhad486	A neural circuit for spatial orientation derived from brain lesions	Disorientation	Lesions	LNM	Lesion network mapping	Heterogeneous
2024	Proc Natl Acad Sci U S A. 2024 Sep 3;121(36):e2322399121. doi: 10.1073/pnas.2322399121	A neural network for religious fundamentalism derived from patients with brain lesions	Fundamentalism	Lesions	sLNM	Lesion network mapping	Heterogeneous
2024	Nat Neurosci. 2024 Nov;27(11):2231-2239. doi: 10.1038/s41593-024-01772-7	A potential target for noninvasive neuromodulation of PTSD symptoms derived from focal brain lesions in veterans	Post-traumatic stress disorder	Lesions	sLNM	Lesion network mapping	Heterogeneous
2024	Neurocase. 2024 Apr;30(2):73-76. doi: 10.1080/13554794.2024.2356892	Acute transitory head mislocalization - a novel syndrome of pathological embodiment in a patient with traumatic brain injury - a case study	Head mislocalization	Lesions	LNM	Lesion network mapping	Not applicable
2024	Brain. 2024 Sep 3;147(9):3048-3058. doi: 10.1093/brain/awae067	Atrophy network mapping of clinical subtypes and main symptoms in frontotemporal dementia	Frontotemporal dementia	Coordinates	LNM	Atrophy network mapping	Heterogeneous
2024	Brain Commun. 2024 Jun 5;6(3):fcae196. doi: 10.1093/braincomms/fcae196	Brain lesions causing parkinsonism versus seizures map to opposite brain networks	Parkinson's disease, Epilepsy	Lesions	cLNM	Lesion network mapping	Not applicable
2024	Brain Stimul. 2024 Nov-Dec;17(6):1171-1177. doi: 10.1016/j.brs.2024.10.004	Brain metabolic response to repetitive transcranial magnetic stimulation to lesion network in cervical dystonia	Dystonia	Lesions, Stimulation sites	LNM	Lesion network mapping	Not applicable
2024	JAMA Neurol. 2024 Sep 30;81(11):1199-209. doi: 10.1001/jamaneurol.2024.2952	Brain networks for cortical atrophy and responsive neurostimulation in temporal lobe epilepsy	Epilepsy	Coordinates	LNM	Atrophy network mapping	Heterogeneous
2024	Biol Psychiatry. 2024 Jun 15;95(12):1091-1099. doi: 10.1016/j.biopsych.2024.01.003	Brain structural and functional damage network localization of suicide	Suicide	Coordinates	LNM	Functional connectivity network mapping	Not mentioned
2024	Res Sq [Preprint]. 2024 Apr 9:rs.3.rs-4221074. doi: 10.21203/rs.3.rs-4221074/v1	Causal network localization of brain stimulation targets for trait anxiety	Anxiety	Lesions, Stimulation sites	sLNM	Lesion network mapping, Causal network localization	Not mentioned
2024	Neuroimage. 2024 Jul 1;294:120641. doi: 10.1016/j.neuroimage.2024.120641	Common neural dysfunction of economic decision-making across psychiatric conditions	Cognition	Coordinates	LNM	Lesion network mapping, Activation network mapping	Heterogeneous
2024	medRxiv [Preprint]. 2024 May 25:2024.05.24.24307896. doi: 10.1101/2024.05.24.24307896	Engaging dystonia networks with subthalamic stimulation	Dystonia	Stimulation sites	LNM	Network mapping	Not applicable
2024	Brain. 2024 Dec 3;147(12):4265-4279. doi: 10.1093/brain/awae315	Enhancing cognitive performance prediction by white matter hyperintensity connectivity assessment	Cerebral small vessel disease	Lesions	LNM	Lesion network mapping	Not mentioned
2024	J Neurosci. 2024 Apr 10;44(15):e1922232024. doi: 10.1523/JNEUROSCI.1922-23.2024	Focal brain lesions causing acquired amusia map to a common brain network	Amusia	Lesions	cLNM	Lesion network mapping	Heterogeneous
2024	Commun Biol. 2024 Jun 7;7(1):700. doi: 10.1038/s42003-024-06399-9	Fronto-thalamic networks and the left ventral thalamic nuclei play a key role in aphasia after thalamic stroke	Aphasia	Lesions	cLNM	Lesion network mapping	Not mentioned
2024	Neurosci Biobehav Rev. 2024 Aug;163:105779. doi: 10.1016/j.neubiorev.2024.105779	Functional brain networks associated with the urge for action: implications for pathological urge	Pathological urge	Lesions	LNM	Coordinate network mapping	Heterogeneous
2024	Schizophr Bull. 2024 Apr 30;50(3):545-556. doi: 10.1093/schbul/sbae003	Heterogeneous brain abnormalities in schizophrenia converge on a common network associated with symptom remission	Schizophrenia	Coordinates	LNM	Coordinate-based network mapping	Heterogeneous
2024	Depress Anxiety. 2024 Apr 2;2024:3827870. doi: 10.1155/2024/3827870	Heterogeneous brain atrophy sites in anxiety disorders map to a common brain network	Anxiety	Coordinates	LNM	Coordinate network mapping	Heterogeneous
2024	Commun Biol. 2024 Oct 5;7(1):1270. doi: 10.1038/s42003-024-06969-x	Heterogeneous brain activations across individuals localize to a common network	Cognition	Coordinates	LNM	Activation network mapping	Heterogeneous
2024	Ann Neurol. 2024 May;95(5):929-940. doi: 10.1002/ana.26894	Increased cortical thickness in Alzheimer's disease	Alzheimer's disease	Coordinates	LNM	Atrophy network mapping, Growth network mapping	Homogeneous
2024	Neurobiol Dis. 2024 Jun 15;196:106521. doi: 10.1016/j.nbd.2024.106521	Indirect functional connectivity does not predict overall survival in glioblastoma	Survival glioblastoma	Lesions	LNM	Functional disconnectivity	Not mentioned
2024	Brain. 2024 Mar 1;147(3):1100-1111. doi: 10.1093/brain/awad402	Integrating direct electrical brain stimulation with the human connectome	Physiology	Stimulation sites	LNM	Lesion network mapping, DBS network mapping	Not applicable
2024	Asian J Psychiatr. 2024 Feb;92:103887. doi: 10.1016/j.ajp.2023.103887	Lesion network guided delta frequency neuromodulation improves cognition in patients with psychosis spectrum disorders: a pilot study	Psychosis	Stimulation sites	LNM	Lesion network mapping	Not applicable
2024	Cortex. 2024 Sep;178:245-248. doi: 10.1016/j.cortex.2024.06.013	Lesion network guided neuromodulation to the extrastriate visual cortex in Charles Bonnet syndrome reduces visual hallucinations: a case study	Psychosis	Stimulation sites	LNM	Causal lesion network, Lesion network analysis	Not applicable
2024	Brain. 2024 Jun 3;147(6):1975-1981. doi: 10.1093/brain/awae094	Lesion network of oculogyric crises maps to brain dopaminergic transcriptomic signature	Oculogyric crises	Lesions	LNM	Lesion network mapping	Heterogeneous
2024	Cortex. 2024 Mar;172:109-113. doi: 10.1016/j.cortex.2023.12.006	Lesion voxels to lesion networks: the enduring value of the vietnam head injury study	Not applicable	Not applicable	Review	Lesion network mapping	Not applicable
2024	Ann Neurol. 2024 Oct;96(4):662-674. doi: 10.1002/ana.27015	Lesions causing Alice in Wonderland Syndrome map to a common brain network linking body and size perception	Alice in Wonderland Syndrome	Lesions	LNM	Lesion network mapping	Heterogeneous
2024	Mov Disord. 2024 Nov;39(11):1971-1981. doi: 10.1002/mds.29940	Linking invasive and noninvasive brain stimulation in Parkinson's disease: a randomized trial	Parkinson's disease	Stimulation sites	LNM	DBS network mapping, Connectomic DBS	Not applicable
2024	Neurology. 2024 Sep 24;103(6):e209803. doi: 10.1212/WNL.0000000000209803	Localization and network connectivity of lesions causing limb ataxia in patients with stroke	Ataxia	Lesions	cLNM	Lesion network mapping	Heterogeneous
2024	Brain. 2024 Jun 3;147(6):2203-2213. doi: 10.1093/brain/awae059	Localization of stuttering based on causal brain lesions	Neurogenic stuttering	Lesions	LNM	Lesion network mapping	Heterogeneous
2024	Biol Psychiatry. 2024 Jun 15;95(12):1072-1080. doi: 10.1016/j.biopsych.2023.11.010	Mapping the relationship of white matter lesions to depression in multiple sclerosis	Depression	Lesions	LNM	Lesion network mapping	Not mentioned
2024	J Neuropsychiatry Clin Neurosci. 2024 Winter;36(1):45-52. doi: 10.1176/appi.neuropsych.20220160	Network localization of spontaneous confabulation	Confabulation	Lesions	LNM	Lesion network mapping	Heterogeneous
2024	Neuropsychologia. 2024 Apr 15;196:108820. doi: 10.1016/j.neuropsychologia.2024.108820	Self-motion induced environmental kinetopsia and pop-out illusion - insight from a single case phenomenology	Cognition	Lesions	LNM	Lesion network mapping	Not applicable
2024	Neurophysiol Clin. 2024 Feb;54(1):102953. doi: 10.1016/j.neucli.2024.102953	Sensory symptoms relieved by navigated rTMS, targeted using lesion network mapping based on human brain connectome, in a patient after thalamic stroke	Sensory symptoms	Stimulation sites	LNM	Lesion network mapping	Not applicable
2024	bioRxiv [Preprint] 2024.04.25.591066. doi: 10.1101/2024.04.25.591066	Spatial neglect after subcortical stroke: sometimes a cortico-cortical disconnection syndrome	Neglect syndrome	Lesions	sLNM	Lesion network symptom mapping, Lesion network mapping	Not mentioned
2024	Ann Clin Transl Neurol. 2024 Feb;11(2):520-524. doi: 10.1002/acn3.51967	The neural correlates of topographical disorientation-a lesion analysis study	Disorientation	Lesions	cLNM	Lesion network mapping	Homogeneous
2024	Epilepsia. 2024 Jun;65(6):1644-1657. doi: 10.1111/epi.17947	Why did my seizures start now? influences of lesion connectivity and genetic etiology on age at seizure onset in focal epilepsy	Epilepsy	Lesions	LNM	Lesion network mapping	Heterogeneous
2025	Nat Commun. 2025 Mar 24;16(1):2783. doi: 10.1038/s41467-025-57392-7	A generalized epilepsy network derived from brain abnormalities and deep brain stimulation	Epilepsy	Coordinates	LNM	Coordinate network mapping	Heterogeneous
2025	medRxiv [Preprint]. 2025 Mar 30:2025-03. doi: 10.1101/2025.03.28.25324852	A human gait circuit derived from brain lesions and deep brain stimulation	Gait	Lesions, Stimulation sites	sLNM	Lesion network mapping, DBS network mapping	Heterogeneous
2025	medRxiv [Preprint]. 2025.02.25.25322842; doi: 10.1101/2025.02.25.25322842	An atlas and evidence-based appraisal framework of psychiatric brain stimulation targets generated by causal network mapping	Not applicable	Not applicable	Review	Causal network mapping, Lesion network mapping	Not applicable
2025	Alzheimers Dement. 2025 Jan 9;20(Suppl 9):e093939. doi: 10.1002/alz.093939.	Atrophy network mapping of anxiety in cognitively unimpaired older adults in preclinical AD	Anxiety	Coordinates	LNM	Atrophy network mapping	Not mentioned
2025	Neurobiol Dis. 2025 Oct 1;214:107031. doi: 10.1016/j.nbd.2025.107031	Brain connectivity regions of lesions causing focal to bilateral tonic-clonic seizures correlates with gene expression	Epilepsy	Lesions	LNM	Lesion network mapping	Heterogeneous
2025	Biol Psychiatry. 2025 Jan 15;97(2):148-156. doi: 10.1016/j.biopsych.2024.07.021	Brain network localization of gray matter atrophy and neurocognitive and social cognitive dysfunction in schizophrenia	Schizophrenia	Coordinates	LNM	Functional connectivity network mapping, Network localization	Not mentioned
2025	Front Psychiatry. 2025 May 8;16:1568895. doi: 10.3389/fpsyt.2025.1568895	Case report: lesion network guided transcranial direct current stimulation targeting treatment refractory hallucinations and delusions: a traditional and accelerated stimulation case study	Psychosis	Stimulation sites	LNM	Lesion network mapping	Not applicable
2025	Epilepsia. 2025 Sep 6. doi: 10.1111/epi.18603	Common network related to hyperkinetic seizures revealed by functional lesion network mapping	Hyperkinetic seizures	Lesions	LNM	Lesion network mapping	Heterogeneous
2025	Nat Commun. 2025 May 22;16(1):4772. doi: 10.1038/s41467-025-60089-6	Convergent mapping of a tremor treatment network	Tremor	Lesions, Stimulation sites	LNM	DBS network mapping	Not mentioned
2025	Ann Child Neurol Soc. 2025 Jun;3(2):91-104. doi: 10.1002/cns3.20108	Coordinate network mapping of focal brain volume differences in ADHD reveals common patterns that lack specificity: a systematic review	Attention-deficit/hyperactivity disorder	Coordinates	LNM	Coordinate network mapping	Heterogeneous
2025	Res Sq [Preprint]. 2025.04.01; doi: 10.21203/rs.3.rs-6261102/v1	Distinct antidepressant therapies act on a common brain network	Depression treatment	Coordinates	LNM	Remission network mapping	Heterogeneous
2025	Ann Neurol. 2025 Feb;97(2):242-253. doi: 10.1002/ana.27129	Distinct constructs underlie patient-reported and performance-rated outcomes after stroke	Stroke	Lesions	sLNM	Lesion network mapping	Not mentioned
2025	Brain. 2025 Mar 21:awaf101. doi: 10.1093/brain/awaf101	Effects of focal brain damage on political behaviour across different political ideologies	Political involvement	Lesions	sLNM	Lesion network mapping	Not mentioned
2025	J Neuropsychiatry Clin Neurosci. 2024 Winter;36(1):74-76. doi: 10.1176/appi.neuropsych.20220145	Focal lesion in the intraparietal sulcus: a case for network-dependent release hallucinations	Psychosis	Lesions	LNM	Lesion network mapping	Not applicable
2025	Front Neurosci. 2025 Apr 9;19:1568045. doi: 10.3389/fnins.2025.1568045	Functional network reorganization and memory impairment in unruptured brain arteriovenous malformations	Cognitive impairment	Lesions	LNM	Lesion network mapping	Not mentioned
2025	Alzheimers Dement. 2025 Mar;21(3):e70073. doi: 10.1002/alz.70073	Heterogeneous brain abnormalities in subjective cognitive decline converge on a common network and their transcriptional signature	Cognitive impairment	Coordinates	LNM	Coordinate network mapping	Heterogeneous
2025	Nat Ment Health. 2025 Jan;3(1):19-30. doi: 10.1038/s44220-024-00348-5	Heterogeneous patterns of brain atrophy in schizophrenia localize to a common brain network	Schizophrenia	Coordinates	LNM	Coordinate network mapping	Heterogeneous
2025	Brain. 2025 Mar 6;148(3):776-787. doi: 10.1093/brain/awae322	Lesion and lesion network localization of dysnomia after epilepsy surgery	Dysnomia	Lesions	LNM	Lesion network mapping, Lesion network localization	Heterogeneous
2025	Brain Commun. 2025;7(3):fcaf228. Published 2025 Jun 13. doi:10.1093/braincomms/fcaf228	Lesion distribution and network mapping in dyskinetic cerebral palsy	Dyskinetic cerebral palsy	Lesions	LNM	Lesion network mapping	Heterogeneous
2025	medRxiv [Preprint]. 2025 Mar 7:2025.03.06.25323494. doi: 10.1101/2025.03.06.25323494	Lesion network localization of functional and somatic symptoms	Functional and somatic symptoms	Lesions	sLNM	Lesion network mapping, Lesion network localization	Not mentioned
2025	Neurol India. 2025 Feb 19. doi: 10.4103/neuroindia.NI_697_20	Lesion network mapping - a novel tool for neurologists in localizing single cases with unusual clinical presentations	Not applicable	Not applicable	Commentary	Lesion network mapping	Not applicable
2025	Neuroimage Clin. 2025;45:103751. doi: 10.1016/j.nicl.2025.103751	Lesion network mapping of REM sleep behaviour disorder	Rapid eye movement sleep behavior disorder	Lesions	LNM	Lesion network mapping	Heterogeneous
2025	Stroke. 2025 Sep;56(9):2527-2539. doi: 10.1161/STROKEAHA.125.051858	Lesion-network mapping of post-stroke depressive symptoms: evidence from two prospective ischemic stroke cohorts	Post-stroke depression	Lesions	cLNM	Lesion network mapping	Not mentioned
2025	Biol Psychiatry. 2025 Jun 15;97(12):1175-1185. doi: 10.1016/j.biopsych.2024.09.022	Mapping lesion-related human aggression to a common brain network	Agression	Lesions	sLNM	Lesion network mapping	Heterogeneous
2025	JAMA Psychiatry. 2025 Apr 1;82(4):368-378. doi: 10.1001/jamapsychiatry.2024.4534	Mapping lesions that cause psychosis to a human brain circuit and proposed stimulation target	Psychosis	Lesions	LNM	Lesion network mapping	Heterogeneous
2025	JAMA Netw Open. 2025 Feb 3;8(2):e2459297. doi: 10.1001/jamanetworkopen.2024.59297	Mapping neuroimaging findings of creativity and brain disease onto a common brain circuit	Creativity	Coordinates	LNM	Coordinate network mapping	Heterogeneous
2025	Neurosci Biobehav Rev. 2025 Sep;176:106272. doi: 10.1016/j.neubiorev.2025.106272	Mapping the psychopathic brain: divergent neuroimaging findings converge onto a common brain network	Psychopathy	Coordinates	LNM	Normative network mapping	Heterogeneous
2025	medRxiv [Preprint] 2025.09.28.678605; doi: 10.1101/2025.09.25.678605	Mapping the structural brain network of psychopathy: convergent evidence from humans and chimpanzees	Psychopathy	Coordinates	LNM	Normative network mapping	Heterogeneous
2025	Ann Neurol. 2025;98(1):152-162. doi: 10.1002/ana.27224	Network localization of pediatric lesion-induced dystonia	Dystonia	Lesions	cLNM	Lesion network mapping, Network localization	Heterogeneous
2025	medRxiv [Preprint] 2025.02.10.25322034; doi: 10.1101/2025.02.10.25322034	Neuroanatomical pathways of TMS therapy for depression	Depression	Stimulation sites	sLNM	Normative connectome	Not applicable
2025	Pain. 2025 May 9. doi: 10.1097/j.pain.0000000000003618	Neuroanatomy and lesion networks of central poststroke pain	Stroke	Lesions	LNM	Lesion network mapping	Homogeneous
2025	medRxiv [Preprint]. 2025 Jan 10:2025.01.09.25320060. doi: 10.1101/2025.01.09.25320060	Obsessive-compulsive disorder secondary to focal brain lesions: from lesions to networks	Obsessive-compulsive disorder	Lesions	LNM	Lesion network analysis	Homogeneous
2025	medRxiv [Preprint]. 2025.02.19.25322521; doi: 10.1101/2025.02.19.25322521	Post stroke cognitive impairment: more than a lesion-symptom model	Stroke	Lesions	LNM	-	Not mentioned
2025	medRxiv [Preprint]. 2025 Aug 08:2025.26.25334501. doi: 10.1101/2025.08.26.25334501	Predicting outcome after newborn stroke: a lesion network mapping study leveraging large-scale data	Neonatal arterial ischemic stroke	Lesions	cLNM	Lesion network mapping	Not mentioned
2025	Epilepsia. 2025 Mar 5. doi: 10.1111/epi.18320	Prognostic application of lesion network mapping to epilepsy surgery outcomes in pediatric tuberous sclerosis complex	Epilepsy	Lesions	sLNM	Lesion network mapping	Not mentioned
2025	Commun Med (Lond). 2025 Mar 12;5(1):69. doi: 10.1038/s43856-025-00760-7	Remission of alcohol use disorder following traumatic brain injury with focal orbitofrontal cortex hemorrhage: case report and network mapping	Alcohol use disorder remission	Lesions	LNM	Lesion network mapping	Not applicable
2025	bioRxiv [Preprint] 2025.02.05.636578. doi: 10.1101/2025.02.05.636578	Remote cortical network for frontal cognitive symptoms derived from Tau accumulation in progressive supranuclear palsy	Progressive supranuclear palsy	Coordinates	LNM	Atrophy network mapping, Lesion network mapping	Homogeneous
2025	Sci Rep. 2025 May 27;15(1):18544. doi: 10.1038/s41598-025-01703-x	Spatial neglect after subcortical stroke may reflect cortico-cortical disconnection	Neglect syndrome	Lesions	sLNM	Lesion network mapping, Lesion network symptom mapping	Not mentioned
2025	Brain Commun. 2025 Aug 8;7(4):fcaf290. doi: 10.1093/braincomms/fcaf290	Structural and functional connectivity of vestibular graviceptive to sensory and motor circuits	Vestibular graviception	Lesions	LNM	Lesion network mapping	Not mentioned
2025	Brain Commun. 2025 May 16;7(3):fcaf191. doi: 10.1093/braincomms/fcaf191	Thalamic disconnection from prefrontal cognitive control networks contributes to thalamic aphasia	Stroke	Lesions	cLNM	Lesion network mapping	Not mentioned
2025	medRxiv [Preprint] 2025.04.07.25325381. doi: 10.1101/2025.04.07.25325381	The deep brain stimulation response network in parkinson's disease operates in the high beta band	Parkinson's disease	Stimulation sites	LNM	DBS network mapping, Electrophysiological network mapping	Not applicable
2025	Nature Mental Health. 2025 Sep 23:1-5. doi: 10.1038/s44220-025-00503-6	The network-based underpinnings of persisting symptoms after concussion: a multimodal neuroimaging meta-analysis	Persisting symptoms after concussion	Coordinates	LNM	Network-based meta-analysis	Heterogeneous
2025	medRxiv [Preprint]. 2025.08.08.25333301; doi: 10.1101/2025.08.08.25333301	Translating the transcriptome: a connectomics approach for gene-network mapping and clinical application	Other	Coordinates	LNM	Gene network mapping	Not applicable
2025	medRxiv [Preprint]. 2025.05.23.25328072; doi: 10.1101/2025.05.23.25328072	Visual mental Imagery and aphantasia lesions map onto a convergent brain network	Aphantasia	Lesions	LNM	Lesion network mapping	Not mentioned

Supplementary Table 2. Source of included lesions and Lesion Network Mapping data. Details of the clinical conditions from which lesion locations and distinct Lesion Network Mapping (LNM) maps (n = 102) were obtained from previously published studies (n = 72). The table includes the condition of the LNM network included (first column), the type of data used to derive the LNM (e.g., segmented lesions, lesion prevalence, brain coordinates, or downloaded LNM networks; second column), the corresponding reference (third column) and data source (fourth column) are indicated. ADHD, attention-deficit/hyperactivity disorder; DBS, deep brain simulation; LNM, Lesion Network Mapping; MS, multiple sclerosis; OCD, obsessive-compulsive disorder; PTSD, post-traumatic stress disorder; sLNM, symptom Lesion Network Mapping; TBI, traumatic brain injury.

Table S2

Condition [map name, Table S4]	Type data	Reference	Source
ADHD<br>[SEGAL_ADHD]	Coordinates	Nat Neurosci. 2023;26(9):1613-1629. doi:10.1038/s41593-023-01404-6	Nat Neurosci. 2023;26(9):1613-1629. doi:10.1038/s41593-023-01404-6
Alcohol use disorder remission<br>[HAQUE_ALCOHOL]	Lesions	Commun Med (Lond). 2025 Mar 12;5(1):69. doi: 10.1038/s43856-025-00760-7	Commun Med (Lond). 2025 Mar 12;5(1):69. doi: 10.1038/s43856-025-00760-7
Alice in Wonderland Syndrome<br>[FRIEDRICH_AIWS]	Lesions	Ann Neurol. 2024;96(4):662-674. doi:10.1002/ana.27015	LesionBank. https://www.lesionbank.org/
Alzheimer's disease<br>[RIOS_ALZHEIMERDBS]	DBS-LNM	Nat Commun. 2022 Dec 14;13(1):7707. doi: 10.1038/s41467-022-34510-3	Lead-DBS - Alzheimer Response Network (Rios 2022)
Amnesia<br>[FERGUSON_AMNESIA]	Lesions	Nat Commun. 2019 Aug 2;10(1):3497. doi: 10.1038/s41467-019-11353-z	LesionBank. https://www.lesionbank.org/
Amusia<br>[SIHVONEN_AMUSIA]	Lesions	J Neurosci. 2024;44(15):e1922232024. Published 2024 Apr 10. doi:10.1523/JNEUROSCI.1922-23.2024	J Neurosci. 2024;44(15):e1922232024. Published 2024 Apr 10. doi:10.1523/JNEUROSCI.1922-23.2024
Anosognosia (visual-motor)<br>[KLETENIK_ANOSOGNOSIA]	sLNM	Ann Neurol. 2023 Sep;94(3):434-441. doi: 10.1002/ana.26709	https://neurovault.org/images/795502/
Anosognosia (visual)<br>[KLETENIK_ANOSOGNOSIA]	Lesions	Ann Neurol. 2023 Sep;94(3):434-441. doi: 10.1002/ana.26709	Ann Neurol. 2023 Sep;94(3):434-441. doi: 10.1002/ana.26709
Anxiety-depression symptoms<br>[SIDDIQI_ANXIETY]	sLNM	Am J Psychiatry. 2020 May 1;177(5):435-446. doi: 10.1176/appi.ajp.2019.19090915	https://neurovault.org/images/787858/
Aphantasia<br>[KUTSCHE_APHANTASIA]	Lesions	medRxiv [Preprint] 2025.05.23.25328072; doi: 10.1101/2025.05.23.25328072	medRxiv [Preprint] 2025.05.23.25328072; doi: 10.1101/2025.05.23.25328072
Aphasia (semantic)<br>[SOUTER_APHASIA]	LNM	Brain Structure and Function, 227(9), 3043-3061. doi: 10.1007/s00429-022-02526-6	https://neurovault.org/images/550103/
Aphasia<br>[ZARIFKAR_APHASIA]	Lesions	Brain Commun. 2023;5(6):fcad288. Published 2023 Oct 27. doi:10.1093/braincomms/fcad288	Brain Commun. 2023;5(6):fcad288. Published 2023 Oct 27. doi:10.1093/braincomms/fcad288
Apnea<br>[YUAN_APNEA]	Lesions	Front Neuroanat. 2022 Sep 29;16:819412. doi: 10.3389/fnana.2022.819412	Front Neuroanat. 2022 Sep 29;16:819412. doi: 10.3389/fnana.2022.819412
Apraxia (eye-opening)<br>[ZARIFKAR_APRAXIA]	Lesions	Brain Commun. 2023;5(6):fcad288. Published 2023 Oct 27. doi:10.1093/braincomms/fcad288	Brain Commun. 2023;5(6):fcad288. Published 2023 Oct 27. doi:10.1093/braincomms/fcad288
Ataxia<br>[LIESMAKI_ATAXIA]	Lesions	Neurology. 2024;103(6):e209803. doi:10.1212/WNL.0000000000209803	Neurology. 2024;103(6):e209803. doi:10.1212/WNL.0000000000209803
Autism spectrum disorder<br>[SEGAL_ASD]	Coordinates	Nat Neurosci. 2023;26(9):1613-1629. doi:10.1038/s41593-023-01404-6	Nat Neurosci. 2023;26(9):1613-1629. doi:10.1038/s41593-023-01404-6
Bipolar disorder<br>[SEGAL_BP]	Coordinates	Nat Neurosci. 2023;26(9):1613-1629. doi:10.1038/s41593-023-01404-6	Nat Neurosci. 2023;26(9):1613-1629. doi:10.1038/s41593-023-01404-6
Blindsight<br>[KLETENIK_BLINDSIGHT]	Lesions	Ann Neurol. 2022;91(2):217-224. doi:10.1002/ana.26292	Ann Neurol. 2022;91(2):217-224. doi:10.1002/ana.26292
Bodily awareness<br>[HERBET_AWARENESS]	Lesions	Brain. 2019;142(8):2207-2214. doi:10.1093/brain/awz179	Brain. 2019;142(8):2207-2214. doi:10.1093/brain/awz179
Coma (cause)<br>[FISCHER_COMACAUSE]	Lesions	Neurology. 2016 Dec 6;87(23):2427-2434. doi: 10.1212/WNL.0000000000003404	Neurology. 2016 Dec 6;87(23):2427-2434. doi: 10.1212/WNL.0000000000003404
Confabulation<br>[ABTEMAN_CONFABULATION]	Lesions	J Neuropsychiatry Clin Neurosci. 2024;36(1):45-52. doi:10.1176/appi.neuropsych.20220160	J Neuropsychiatry Clin Neurosci. 2024;36(1):45-52. doi:10.1176/appi.neuropsych.20220160
Creativity<br>[KUTSCHE_CREATIVITY]	Lesion prevalence	JAMA Netw Open. 2025;8(2):e2459297. Published 2025 Feb 3. doi:10.1001/jamanetworkopen.2024.59297	JAMA Netw Open. 2025;8(2):e2459297. Published 2025 Feb 3. doi:10.1001/jamanetworkopen.2024.59297
Creativity<br>[KUTSCHE_CREATIVITY]	Coordinates	JAMA Netw Open. 2025;8(2):e2459297. Published 2025 Feb 3. doi:10.1001/jamanetworkopen.2024.59297	JAMA Netw Open. 2025;8(2):e2459297. Published 2025 Feb 3. doi:10.1001/jamanetworkopen.2024.59297
Criminality<br>[DARBY_CRIMINALITY]	Lesions	Proc Natl Acad Sci U S A. 2018;115(3):601-606. doi:10.1073/pnas.1706587115	Proc Natl Acad Sci U S A. 2018;115(3):601-606. doi:10.1073/pnas.1706587115
Delusional misidentifications<br>[DARBY_DELUSION]	Lesions	Brain. 2017 Feb;140(2):497-507. doi: 10.1093/brain/aww288	Brain. 2017 Feb;140(2):497-507. doi: 10.1093/brain/aww288
Depression<br>[SEGAL_MDD]	Coordinates	Nat Neurosci. 2023;26(9):1613-1629. doi:10.1038/s41593-023-01404-6	Nat Neurosci. 2023;26(9):1613-1629. doi:10.1038/s41593-023-01404-6
Depression<br>[LI_DEPRESSION]	Lesions	Brain Struct Funct. 2022;227(8):2787-2795. doi:10.1007/s00429-022-02573-z	Brain Struct Funct. 2022;227(8):2787-2795. doi:10.1007/s00429-022-02573-z
Depression<br>[SIDDIQI_DEPRESSION]	sLNM	Nat Hum Behav. 2021 Dec;5(12):1707-1716. doi: 10.1038/s41562-021-01161-1	https://neurovault.org/images/787859/
Depression<br>[FOX_DLPFCTMS]	Coordinates, clinical symptoms	Biol Psychiatry. 2012 Oct 1;72(7):595-603. doi: 10.1016/j.biopsych.2012.04.028	Biol Psychiatry. 2018 Jul 1;84(1):28-37. doi: 10.1016/j.biopsych.2017.10.028
Depression (cognitive, emotional)<br>[CASH_DEPRESSIONCOG, CASH_DEPRESSIONEMO]	Coordinates	Nat Ment Health. 2023 Mar;1(3):174-185. doi: 10.1038/s44220-023-00038-8	JAMA Psychiatry. 2017 Jan 1;74(1):47-55. doi: 10.1001/jamapsychiatry.2016.2783
Depression (risk, resilience)<br>[TRAPP_DEPRESSIONPOS, TRAPP_DEPRESSIONNEG]	Coordinates	Brain. 2023 Apr 19;146(4):1672-1685. doi: 10.1093/brain/awac361	Brain. 2023 Apr 19;146(4):1672-1685. doi: 10.1093/brain/awac361
Depression remission<br>[JI_ANTIDEPRESSANT]	LNM	ResearchSquare [Preprint] 2025.04.01; doi: 10.21203/rs.3.rs-6261102/v1	https://github.com/jigongjun/Depression-therapeutic-network/blob/main/DTN.nii
Disrupted free will (agency, volition)<br>[DARBY_AGENCY, DARBY_VOLITION]	Lesions	Proc Natl Acad Sci U S A. 2018 Oct 16;115(42):10792-10797. doi: 10.1073/pnas.1814117115	Proc Natl Acad Sci U S A. 2018 Oct 16;115(42):10792-10797. doi: 10.1073/pnas.1814117115
Dyskinetic cerebral palsy<br>[MARCELINO_PALSY]	Lesions	Brain Commun. 2025;7(3):fcaf228. Published 2025 Jun 13. doi:10.1093/braincomms/fcaf228	Brain Commun. 2025;7(3):fcaf228. Published 2025 Jun 13. doi:10.1093/braincomms/fcaf228
Dystonia (generalized)<br>[HORN_DYSTONIAGENERALIZEDDBS]	DBS-LNM	Proc Natl Acad Sci U S A. 2022 Apr 5;119(14):e2114985119. doi: 10.1073/pnas.2114985119	Lead-DBS - Dystonia Response Tract Atlas (Horn 2022)
Dystonia (cervical)<br>[CORP_DYSTONIA]	Lesions	Brain. 2019;142(6):1660-1674. doi:10.1093/brain/awz112	Brain. 2019;142(6):1660-1674. doi:10.1093/brain/awz112
Epilepsy<br>[JI_EPILEPSY]	LNM	Nat Commun. 2025 Mar 24;16(1):2783. doi: 10.1038/s41467-025-57392-7	https://github.com/jigongjun/IGENetwork/blob/main/IGEnetwork.nii
Epilepsy<br>[JI_EPILEPSY]	Coordinates	Nat Commun. 2025 Mar 24;16(1):2783. doi: 10.1038/s41467-025-57392-7	Nat Commun. 2025 Mar 24;16(1):2783. doi: 10.1038/s41467-025-57392-7
Epilepsy<br>[MANSOURI_EPILEPSY]	Lesions	Sci Rep. 2020;10(1):10989. Published 2020 Jul 3. doi:10.1038/s41598-020-67626-x	Sci Rep. 2020;10(1):10989. Published 2020 Jul 3. doi:10.1038/s41598-020-67626-x
Epilepsy<br>[SUN_EPILEPSY]	Lesions	Neurobiol Dis. 2025 Oct 1;214:107031. doi: 10.1016/j.nbd.2025.107031	Neurobiol Dis. 2025 Oct 1;214:107031. doi: 10.1016/j.nbd.2025.107031
Facial processing (emotion, non-emotion, control rumination)<br>[GONG_EMOTION, GONG_NONEMOTION, GONG_CONTROLFORRUMINATION]	Coordinates	Nat Hum Behav. 2022 Oct;6(10):1417-1429. doi: 10.1038/s41562-022-01371-1	https://github.com/sailingpeng/2021_ActivationNetworkMapping/blob/main/Coordinate_data.mat
Foreign accent syndrome<br>[HIGASHIYAMA_ACCENT]	Lesions	Neuroimage Clin. 2021;31:102760. doi:10.1016/j.nicl.2021.102760	Neuroimage Clin. 2021;31:102760. doi:10.1016/j.nicl.2021.102760
Freezing gait<br>[FASANO_GAIT]	Lesions	Ann Neurol. 2017;81(1):129-141. doi:10.1002/ana.24845	Ann Neurol. 2017;81(1):129-141. doi:10.1002/ana.24845
Functional and somatic symptoms (set 1, set 2)<br>[MILANO_SOMATICSET1, MILANO_SOMATICSET2]	Lesion prevalence	medRxiv [Preprint]. 2025 Mar 7:2025.03.06.25323494. doi: 10.1101/2025.03.06.25323494	medRxiv [Preprint]. 2025 Mar 7:2025.03.06.25323494. doi: 10.1101/2025.03.06.25323494
Healthy control<br>[SEGAL_HCtest]	Coordinates	Nat Neurosci. 2023;26(9):1613-1629. doi:10.1038/s41593-023-01404-6	Nat Neurosci. 2023;26(9):1613-1629. doi:10.1038/s41593-023-01404-6
Hemichorea-hemiballismus<br>[LAGANIERE_HEMICHOREA]	Lesions	Neurology. 2016;86(23):2187-2195. doi:10.1212/WNL.0000000000002741	Neurology. 2016;86(23):2187-2195. doi:10.1212/WNL.0000000000002741
Holmes tremor<br>[JOUTSA_HOLMES]	Lesions	Ann Neurol. 2019;86(6):812-820. doi:10.1002/ana.25618	Ann Neurol. 2019;86(6):812-820. doi:10.1002/ana.25618
Hypersomnia<br>[LESIONBANK_HYPERSOMNIA]	Lesions	LesionBank. https://www.lesionbank.org/	LesionBank. https://www.lesionbank.org/
Insomnia<br>[LESIONBANK_INSOMNIA]	Lesions	LesionBank. https://www.lesionbank.org/	LesionBank. https://www.lesionbank.org/
Mania<br>[LEE_MANIA]	Lesions	J Affect Disord. 2019;244:115-123. doi:10.1016/j.jad.2018.10.087	J Affect Disord. 2019;244:115-123. doi:10.1016/j.jad.2018.10.087
Mania (set a, set b)<br>[COTOVIO_MANIASETA, COTOVIO_MANIASETB]	Lesion prevalence	J Clin Invest. 2020;130(10):5209-5222. doi:10.1172/JCI136096	J Clin Invest. 2020;130(10):5209-5222. doi:10.1172/JCI136096
Migraine<br>[BURKE_MIGRAINE]	Coordinates	Brain. 2020 Feb 1;143(2):541-553. doi: 10.1093/brain/awz405	Brain. 2020 Feb 1;143(2):541-553. doi: 10.1093/brain/awz405
MS (depression)<br>[SIDDIQI_MSDEPRESSION]	sLNM	Nat Ment Health. 2023 Jan;1(3):36-44. doi: 10.1038/s44220-022-00002-y	https://neurovault.org/images/787866/
MS (memory impairment)<br>[KLETENIK_MS]	sLNM	J Neurol. 2023 Nov;270(11):5211-5222. doi: 10.1007/s00415-023-11907-8	https://neurovault.org/images/840244/
Neglect syndrome<br>[LESIONBANK_NEGLECT]	Lesions	LesionBank. https://www.lesionbank.org/	LesionBank. https://www.lesionbank.org/
Neurogenic stuttering<br>[THEYS_STUTTERING]	Lesions	Brain. 2024 Jun 3;147(6):2203-2213. doi: 10.1093/brain/awae059	Brain. 2024 Jun 3;147(6):2203-2213. doi: 10.1093/brain/awae059
OCD<br>[LI_OCDDBS]	DBS-LNM	Nat Commun. 2020 Jul 3;11(1):3364. doi: 10.1038/s41467-020-16734-3	Lead-DBS - OCD Response Tract Atlas (Li 2020)
OCD<br>[SEGAL_OCD]	Coordinates	Nat Neurosci. 2023;26(9):1613-1629. doi:10.1038/s41593-023-01404-6	Nat Neurosci. 2023;26(9):1613-1629. doi:10.1038/s41593-023-01404-6
OCD<br>[COTOVIO_OCD]	Lesion prevalence	medRxiv [Preprint]. 2025 Jan 10:2025.01.09.25320060. doi: 10.1101/2025.01.09.25320060	medRxiv [Preprint]. 2025 Jan 10:2025.01.09.25320060. doi: 10.1101/2025.01.09.25320060
OCD (cause)<br>[GERMANN_OCDCAUSE]	Lesions	Brain. 2021;144(11):3529-3540. doi:10.1093/brain/awab232	Brain. 2021;144(11):3529-3540. doi:10.1093/brain/awab232
Oculogyric crises<br>[ALFATLY_OCULOGYRIC]	Lesions	Brain. 2024;147(6):1975-1981. doi:10.1093/brain/awae094	Brain. 2024;147(6):1975-1981. doi:10.1093/brain/awae094
Parkinson's disease (cognitive decline)<br>[REICH_PARKINSONDBS]	DBS-LNM	Brain. 2022 May 24;145(4):1410-1421. doi: 10.1093/brain/awac012	Lead-DBS - Cognitive Decline Network Atlas (Reich 2022)
Parkinsonism<br>[JOUTSA_PARKINSONISM]	Lesions	Brain. 2018;141(8):2445-2456. doi:10.1093/brain/awy161	Brain. 2018;141(8):2445-2456. doi:10.1093/brain/awy161
Pathological urge (blink, cough, micturition, swallow)<br>[ZOUKI_BLINK, ZOUKI_COUGH, ZOUKI_MICTURITION, ZOUKI_SWALLOW]	Coordinates	Brain. 2021 Nov 29;144(10):3264-3276. doi: 10.1093/brain/awab224.	https://neurovault.org/collections/GOKZXUGP/
Peduncular hallucinosis<br>[BOES_HALLUCINOSIS]	Lesions	Brain. 2015;138(Pt 10):3061-3075. doi:10.1093/brain/awv228	Brain. 2015;138(Pt 10):3061-3075. doi:10.1093/brain/awv228
Persisting symptoms after concussion (high, low)<br>[MOLLICA_PSAC+, MOLLICA_PSAC-]	Coordinates	Nature Mental Health. 2025 Sep 23:1-5. doi: 10.1038/s44220-025-00503-6	Nature Mental Health. 2025 Sep 23:1-5. doi: 10.1038/s44220-025-00503-6
Political involvement<br>[SIDDIQI_POLITICAL]	sLNM	Brain. 2025 Mar 21:awaf101. doi: 10.1093/brain/awaf101	https://neurovault.org/images/899144/
Post-stroke depression (set a, set b)<br>[RIOS_STROKESETA, RIOS_STROKESETB]	Lesion prevalence	Stroke. Published online July 7, 2025. doi:10.1161/STROKEAHA.125.051858	Stroke. Published online July 7, 2025. doi:10.1161/STROKEAHA.125.051858
Prosopagnosia<br>[COHEN_PROSOPAGNOSIA]	Lesions	Brain. 2019;142(12):3975-3990. doi:10.1093/brain/awz332	Brain. 2019;142(12):3975-3990. doi:10.1093/brain/awz332
Psychopathy<br>[DUGRE_PSYCHOPATHY]	Lesion prevalence	Neurosci Biobehav Rev. 2025 Sep;176:106272. doi: 10.1016/j.neubiorev.2025.106272	Neurosci Biobehav Rev. 2025 Sep;176:106272. doi: 10.1016/j.neubiorev.2025.106272
Psychosis<br>[PINES_PSYCHOSIS]	Lesions	JAMA Psychiatry. 2025 Apr 1;82(4):368-378. doi: 10.1001/jamapsychiatry.2024.4534	JAMA Psychiatry. 2025 Apr 1;82(4):368-378. doi: 10.1001/jamapsychiatry.2024.4534
Psychosis<br>[PINES_PSYCHOSIS]	LNM	JAMA Psychiatry. 2025 Apr 1;82(4):368-378. doi: 10.1001/jamapsychiatry.2024.4534	https://neurovault.org/images/899080/
PTSD<br>[SIDDIQI_PTSD]	Lesion prevalence	Nat Neurosci. 2024 Nov;27(11):2231-2239. doi: 10.1038/s41593-024-01772-7	Nat Neurosci. 2024 Nov;27(11):2231-2239. doi: 10.1038/s41593-024-01772-7
PTSD<br>[SIDDIQI_PTSD]	sLNM	Nat Neurosci. 2024 Nov;27(11):2231-2239. doi: 10.1038/s41593-024-01772-7	https://neurovault.org/images/899936/
Rapid eye movement sleep behavior disorder<br>[YUAN_RBD]	Lesions	Korean J Radiol. 2023;24(3):247-258. doi:10.3348/kjr.2022.0712	Korean J Radiol. 2023;24(3):247-258. doi:10.3348/kjr.2022.0712
Schizophrenia<br>[SEGAL_SCZ]	Coordinates	Nat Neurosci. 2023;26(9):1613-1629. doi:10.1038/s41593-023-01404-6	Nat Neurosci. 2023;26(9):1613-1629. doi:10.1038/s41593-023-01404-6
Smoking addiction (contrast: A-B)<br>[JOUTSA_ADDICTIONCONTRASTAB]	Lesions	Nat Med. 2022 Jun;28(6):1249-1255. doi: 10.1038/s41591-022-01834-y	https://doi.org/10.7910/DVN/8BHHRS
Smoking addiction (A, remission for smoking)<br>[JOUTSA_ADDICTIONGROUPA]	Lesions	Nat Med. 2022 Jun;28(6):1249-1255. doi: 10.1038/s41591-022-01834-y	https://doi.org/10.7910/DVN/8BHHRS
Smoking addiction (B, not quit smoking)<br>[JOUTSA_ADDICTIONGROUPB]	Lesions	Nat Med. 2022 Jun;28(6):1249-1255. doi: 10.1038/s41591-022-01834-y	https://doi.org/10.7910/DVN/8BHHRS
Smoking addiction (C, quit but did not remit)<br>[JOUTSA_ADDICTIONGROUPC]	Lesions	Nat Med. 2022 Jun;28(6):1249-1255. doi: 10.1038/s41591-022-01834-y	https://doi.org/10.7910/DVN/8BHHRS
Spasticity<br>[QIN_SPASTICITY]	Lesions	Front Aging Neurosci. 2022;14:1011812. Published 2022 Oct 25. doi:10.3389/fnagi.2022.1011812	Front Aging Neurosci. 2022;14:1011812. Published 2022 Oct 25. doi:10.3389/fnagi.2022.1011812
Substance abuse disorder<br>[STUBB_SUD]	LNM	Nat Ment Health 2023 Oct;1(10):772-81. doi: 10.1038/s44220-023-00128-7	https://github.com/nimlab/NMH_Stubbs2023
TBI<br>[CRISTOFORI_TBI]	Lesion prevalence	Cortex. 2024 May;174:164-188. doi: 10.1016/j.cortex.2024.03.002	Cortex. 2024 May;174:164-188. doi: 10.1016/j.cortex.2024.03.002
Tics<br>[GANOS_TICS]	Lesions	Brain. 2022;145(12):4385-4397. doi:10.1093/brain/awac009	Brain. 2022;145(12):4385-4397. doi:10.1093/brain/awac009
Tics (Tourette syndrome)<br>[ZOUKI_TICS]	Lesions	Brain Commun. 2023;5(3):fcad105. Published 2023 Apr 4. doi:10.1093/braincomms/fcad105	Brain Commun. 2023;5(3):fcad105. Published 2023 Apr 4. doi:10.1093/braincomms/fcad105
Transdiagnostic psychiatric disorders<br>[TAYLOR_TRANSDIAGNOSTIC]	LNM	Nat Hum Behav. 2023 Mar;7(3):420-429. doi: 10.1038/s41562-022-01501-9	https://github.com/nimlab/NHB_Taylor2023/blob/master/results/TransdiagnosticNetwork_Taylor2023_NHB.nii
Transient global amnesia<br>[WANG_AMNESIA]	Lesions	Neurol Sci. 2023;44(2):649-657. doi:10.1007/s10072-022-06439-4	Neurol Sci. 2023;44(2):649-657. doi:10.1007/s10072-022-06439-4
Tremor<br>[JOUTSA_TREMOR]	sLNM	Ann Neurol. 2018;84(1):153-157. doi:10.1002/ana.25285	Lead-DBS - Essential Tremor Lesion Network Atlas (Joutsa 2018)
Tremor<br>[JOUTSA_TREMOR]	Lesions	Ann Neurol. 2018;84(1):153-157. doi:10.1002/ana.25285	Ann Neurol. 2018;84(1):153-157. doi:10.1002/ana.25285
Vertigo<br>[LI_VERTIGO]	Lesions	Brain Commun. 2023;5(2):fcad071. Published 2023 Mar 17. doi:10.1093/braincomms/fcad071	Brain Commun. 2023;5(2):fcad071. Published 2023 Mar 17. doi:10.1093/braincomms/fcad071

Supplementary Table 3. Overlap between Lesion Network Mapping maps.
For each pair of Lesion Network Mapping (LNM) maps, the corresponding conditions are listed in the first and second columns. The voxel-wise correlation between the two networks was quantified using Pearson's correlation coefficient (third column) and its associated *p*-value (fourth column). To assess significance, two null models were applied: a spin test, in which one network was spatially rotated on the cortical surface while preserving contiguity (fifth column), and BrainSMASH, which generates surrogate brain maps that preserve the spatial autocorrelation of the original data (sixth column). Across these comparisons, all network pairs showed significant correlations, indicating that LNM networks derived from different conditions frequently overlap. MS, multiple sclerosis; LNM, Lesion Network Mapping; OCD, obsessive-compulsive disorder; PTSD, post-traumatic stress disorder.

Table S3

**Map 1**	**Map 2**	r	p	p_{spin}	$p_{brainsmash}$
PTSD	Cognitive decline in Parkinson's disease (DBS)	0.73	<0.0001	<0.0001	<0.0001
Smoking addiction (A, remission for smoking)	Migraine	0.89	<0.0001	<0.0001	<0.0001
Smoking addiction (A, remission for smoking)	Disrupted agency	0.76	<0.0001	<0.0001	<0.0001
Smoking addiction (A, remission for smoking)	Neurogenic stuttering	0.80	<0.0001	<0.0001	<0.0001
Migraine	Disrupted agency	0.65	<0.0001	<0.0001	<0.0001
Migraine	Neurogenic stuttering	0.67	<0.0001	<0.0001	<0.0001
Disrupted agency	Neurogenic stuttering	0.62	<0.0001	<0.0001	<0.0001
Aphasia (semantic)	Epilepsy	0.40	<0.0001	<0.0001	<0.0001
Psychosis	Amnesia	0.80	<0.0001	<0.0001	<0.0001
MS (depression)	Smoking addiction (contrast: A-B)	0.57	<0.0001	<0.0001	<0.0001
OCD (DBS)	Dyskinetic cerebral palsy	0.64	<0.0001	<0.0001	<0.0001

Supplementary Table 4. Overlap between Lesion Network Mapping networks and the degree of the normative connectome and modular organization.

For each of the examined LNM maps (102 LNM maps, 99 unique; first column), corresponding to a specific condition (second column), the number of lesions, coordinates, or subjects is reported (third column; a dash indicates group comparison-based maps). The voxel-wise correlation between each LNM map and the row-summation vector of the normative functional connectome *C* (“degree”) was quantified using Pearson’s correlation coefficient (fourth column) and its associated *p*-value (ninth column). A regression model was used to predict each LNM map from the cortical and subcortical degree of the GSP1000 normative connectome, four functional modules, and three gradients. The maximum correlation with a module is shown (fifth column), together with the percentage of variance explained using degree alone (sixth column) and the full model (voxel-wise, seventh column; atlas-based, eighth column). The atlas-based analysis was performed only for LNM maps with available lesion data. On average, the full model explained 81% (voxel-wise) and 94% (atlas-based) of the variance, suggesting that LNM maps are largely explained by general futures of the normative connectome rather than by disease-specific information. To further evaluate the significance of the association between voxel-wise LNM maps and degree, two complementary null-models were applied: a spin test, in which brain maps were rotated on the cortical surface (tenth column), and BrainSMASH, which generates surrogate brain maps preserving the spatial autocorrelation of the original data (eleventh column). These analyses indicate that nearly all examined LNM networks reflect the total degree sequence of the connectivity matrix, with 78 networks significant under the spin test ($p_{spin} < 0.05$, two-sided) and 91 networks reaching significance under BrainSMASH ($p_{brainsmash} < 0.05$, two-sided). ADHD, attention-deficit/hyperactivity disorder; MS, multiple sclerosis; LNM, Lesion Network Mapping; OCD, obsessive-compulsive disorder; PTSD, post-traumatic stress disorder; TBI, traumatic brain injury. *Table is presented on next page.*

Table S4

Map name	Condition	No. lesions/ coordinates/subjects	r with degree	Max. r with module	Explained variance regress model (degree)	Explained variance regress model (full, voxel-wise)	Explained variance regress model (full, atlas-based)	p (with degree)	p_{spin} (with degree)	$p_{brainsmash}$ (with degree)
ABTEMAN_CONFABULATION	Confabulation	25	-0.12	0.66	27.5%	84.3%	91.1%	<0.0001	0.0073	0.0578
ALFATLY_OCULOGYRIC	Oculogyric crises	14	0.30	0.22	0.4%	86.1%	95.4%	<0.0001	0.6558	<0.0001
BOES_HALLUCINOSIS	Peduncular hallucinosis	23	0.13	0.20	2.7%	83.1%	93.6%	<0.0001	0.2664	0.0365
BURKE_MIGRAINE	Migraine	11	0.70	0.78	41.9%	88.4%	97.7%	<0.0001	<0.0001	<0.0001
CASH_DEPRESSIONCOG	Depression (cognitive)	126	0.58	0.72	41.3%	83.7%	90.3%	<0.0001	<0.0001	<0.0001
CASH_DEPRESSIONEMO	Depression (emotional)	225	0.74	0.71	61.3%	84.7%	88.0%	<0.0001	<0.0001	<0.0001
COHEN_PROSOPAGNOSIA	Prosopagnosia	44	0.34	0.84	6.1%	88.8%	96.9%	<0.0001	0.0760	<0.0001
CORP_DYSTONIA	Dystonia (cervical)	25	0.00	0.13	15.0%	85.0%	94.1%	0.5489	0.1474	0.9835
COTOVIO_MANIASETA	Mania (set a)	41	0.73	0.79	53.9%	89.7%	95.2%	<0.0001	<0.0001	<0.0001
COTOVIO_MANIASETB	Mania (set b)	15	0.71	0.82	84.0%	92.5%	98.9%	<0.0001	<0.0001	<0.0001
COTOVIO_OCD	OCD	40	0.72	0.79	42.5%	89.2%	96.8%	<0.0001	<0.0001	<0.0001
CRISTOFORI_TBI	TBI	134	0.10	0.32	0.7%	90.4%	95.5%	<0.0001	0.9666	0.1091
DARBY_AGENCY	Disrupted agency	48	0.59	0.63	62.0%	80.2%	84.3%	<0.0001	<0.0001	<0.0001
DARBY_CRIMINALITY	Criminality	17	-0.40	0.87	42.0%	85.1%	94.6%	<0.0001	<0.0001	<0.0001
DARBY_DELUSION	Delusional misidentifications	17	0.65	0.67	26.4%	77.3%	90.4%	<0.0001	<0.0001	<0.0001
DARBY_VOLITION	Disrupted volition	28	0.05	0.35	5.5%	86.0%	91.7%	<0.0001	0.1787	0.4198
DUGRE_PSYCHOPATHY	Psychopathy	40	0.45	0.43	2.1%	82.9%	87.7%	<0.0001	0.0808	<0.0001
FASANO_GAIT	Freezing gait	14	0.44	0.44	12.2%	80.5%	89.6%	<0.0001	0.0007	<0.0001
FERGUSON_AMNESIA	Amnesia	53	-0.12	0.46	11.6%	86.3%	93.3%	<0.0001	0.0004	0.0493
FISCHER_COMACAUSE	Coma (cause)	12	-0.08	0.37	16.5%	53.9%	81.3%	<0.0001	0.0083	0.1624
FOX_DLPFCTMS	Depression	131	0.53	0.74	28.1%	75.5%	-	<0.0001	<0.0001	<0.0001
FRIEDRICH_AIWS	Alice in Wonderland Syndrome	24	0.36	0.93	9.9%	92.8%	98.4%	<0.0001	0.0967	<0.0001
GANOS_TICS	Tics	22	0.53	0.60	13.3%	90.1%	97.1%	<0.0001	<0.0001	<0.0001
GERMANN_OCDCAUSE	OCD (cause)	14	0.42	0.40	1.5%	89.3%	94.6%	<0.0001	0.0362	<0.0001
GONG_CONTROLFORRUMINATION	Facial processing (control rumination)	200	0.76	0.85	73.5%	87.3%	89.7%	<0.0001	<0.0001	<0.0001
GONG_EMOTION	Facial processing (emotion)	223	0.77	0.59	54.5%	83.0%	83.4%	<0.0001	<0.0001	<0.0001
GONG_NONEMOTION	Facial processing (non-emotion)	145	0.75	0.87	75.1%	88.0%	93.3%	<0.0001	<0.0001	<0.0001
HAQUE_ALCOHOL	Alcohol use disorder remission	1	-0.36	0.78	40.9%	74.8%	77.1%	<0.0001	<0.0001	<0.0001
HERBET_AWARENESS	Bodily awareness	14	-0.47	0.54	8.8%	76.9%	76.1%	<0.0001	<0.0001	<0.0001
HIGASHIYAMA_ACCENT	Foreign accent syndrome	25	0.78	0.82	63.9%	88.1%	90.0%	<0.0001	<0.0001	<0.0001
HORN_DYSTONIAGENERALIZEDDBS	Dystonia (generalized)	80	-0.10	0.23	3.4%	57.3%	-	<0.0001	0.1615	0.051
JI_ANTIDEPRESSANT	Depression remission	144	0.54	0.62	31.1%	76.6%	-	<0.0001	<0.0001	<0.0001
JI_EPILEPSY	Epilepsy (coordinates)	21	0.82	0.92	71.0%	93.5%	98.7%	<0.0001	<0.0001	<0.0001
JI_EPILEPSY	Epilepsy (LNM)	21	0.66	0.74	50.0%	85.7%	-	<0.0001	<0.0001	<0.0001
JOUTSA_ADDICTIONCONTRASTAB	Smoking addiction (contrast: A-B)	-	0.63	0.88	57.3%	85.2%	-	<0.0001	<0.0001	<0.0001
JOUTSA_ADDICTIONGROUPA	Smoking addiction (A, remission for smoking)	34	0.81	0.81	56.2%	88.7%	97.4%	<0.0001	<0.0001	<0.0001
JOUTSA_ADDICTIONGROUPB	Smoking addiction (B, not quit smoking)	69	0.70	0.66	20.0%	87.9%	95.7%	<0.0001	<0.0001	<0.0001
JOUTSA_ADDICTIONGROUPC	Smoking addiction (C, quit but did not remit)	26	0.82	0.57	48.8%	83.3%	96.5%	<0.0001	<0.0001	<0.0001
JOUTSA_HOLMES	Holmes tremor	36	0.01	0.19	5.1%	78.8%	90.7%	<0.0001	0.1246	0.8163
JOUTSA_PARKINSONISM	Parkinsonism	29	0.29	0.23	0.0%	89.0%	97.3%	<0.0001	0.6711	<0.0001
JOUTSA_TREMOR	Tremor (lesions)	11	0.47	0.62	29.4%	59.5%	95.7%	<0.0001	<0.0001	<0.0001
JOUTSA_TREMOR	Tremor (sLNM)	11	0.70	0.71	51.9%	84.2%	-	<0.0001	<0.0001	<0.0001
KLETENIK_ANOSOGNOSIA	Anosognosia (lesions)	24	0.26	0.61	10.8%	95.1%	96.2%	<0.0001	0.3357	<0.0001
KLETENIK_ANOSOGNOSIA	Anosognosia (sLNM)	267	0.36	0.90	5.5%	90.0%	-	<0.0001	0.0306	<0.0001
KLETENIK_BLINDSIGHT	Blindsight	34	0.28	0.88	5.7%	85.5%	92.0%	<0.0001	0.1686	<0.0001
KLETENIK_MS	MS (memory impairment)	431	-0.04	0.58	0.9%	76.1%	-	<0.0001	0.4356	<0.0001
KUTSCHE_APHANTASIA	Aphantasia	12	0.39	0.88	15.8%	90.3%	98.0%	<0.0001	0.0107	<0.0001
KUTSCHE_CREATIVITY	Creativity (coordinates)	35	0.58	0.76	32.3%	80.3%	86.3%	<0.0001	<0.0001	<0.0001
KUTSCHE_CREATIVITY	Creativity (prevalence)	56	0.55	0.67	23.4%	90.4%	96.3%	<0.0001	<0.0001	<0.0001
LAGANIERE_HEMICHOREA	Hemichorea-hemiballismus	29	0.61	0.62	21.3%	89.6%	97.4%	<0.0001	<0.0001	<0.0001
LEE_MANIA	Mania	23	0.59	0.40	7.6%	78.2%	91.7%	<0.0001	<0.0001	<0.0001
LESIONBANK_HYPERSOMNIA	Hypersomnia	22	0.41	0.47	6.0%	84.2%	89.9%	<0.0001	0.0603	<0.0001
LESIONBANK_INSOMNIA	Insomnia	12	0.49	0.33	2.6%	84.2%	90.5%	<0.0001	0.0003	<0.0001
LESIONBANK_NEGLECT	Neglect syndrome	34	0.70	0.75	53.5%	68.1%	90.5%	<0.0001	<0.0001	<0.0001
LI_DEPRESSION	Depression	18	0.29	0.23	0.0%	80.8%	90.8%	<0.0001	0.9010	<0.0001
LI_OCDDBS	OCD	50	0.39	0.66	15.3%	66.9%	-	<0.0001	0.0006	<0.0001
LI_VERTIGO	Vertigo	23	0.78	0.76	54.1%	89.2%	95.4%	<0.0001	<0.0001	<0.0001
LIESMAKI_ATAXIA	Ataxia	34	0.51	0.63	20.1%	80.8%	96.4%	<0.0001	<0.0001	<0.0001
MANSOURI_EPILEPSY	Epilepsy	23	0.39	0.66	7.4%	83.1%	91.8%	<0.0001	0.0061	<0.0001
MARCELINO_PALSY	Dyskinetic cerebral palsy	23	0.60	0.58	21.2%	84.6%	93.0%	<0.0001	<0.0001	<0.0001
MILANO_SOMATICSET1	Functional and somatic symptoms (set 1)	101	0.55	0.85	82.7%	92.4%	99.3%	<0.0001	<0.0001	<0.0001
MILANO_SOMATICSET2	Functional and somatic symptoms (set 2)	181	0.75	0.66	37.3%	89.7%	96.1%	<0.0001	<0.0001	<0.0001
MOLLICA_PSAC+	Persisting symptoms after concussion (high)	178	0.50	0.66	33.4%	79.5%	95.8%	<0.0001	<0.0001	<0.0001
MOLLICA_PSAC-	Persisting symptoms after concussion (low)	67	0.00	0.55	3.0%	84.1%	91.8%	0.6045	0.1586	0.9863
PINES_PSYCHOSIS	Psychosis (lesions)	104	0.15	0.41	3.5%	80.4%	86.6%	<0.0001	0.3865	0.0243
PINES_PSYCHOSIS	Psychosis (LNM)	153	-0.52	0.83	53.2%	91.4%	-	<0.0001	<0.0001	<0.0001
QIN_SPASTICITY	Spasticity	32	0.68	0.73	35.9%	89.5%	96.6%	<0.0001	0.0001	<0.0001
REICH_PARKINSONDBS	Parkinson's disease (cognitive decline)	10	0.64	0.92	75.1%	96.6%	-	<0.0001	<0.0001	<0.0001
RIOS_ALZHEIMERDBS	Alzheimer's disease	46	-0.08	0.47	7.0%	76.6%	-	<0.0001	0.0282	<0.0001
RIOS_STROKESETA	Post-stroke depression (set a)	97	0.50	0.82	76.2%	91.7%	98.7%	<0.0001	<0.0001	<0.0001
RIOS_STROKESETB	Post-stroke depression (set b)	280	0.68	0.87	74.7%	92.3%	98.7%	<0.0001	<0.0001	<0.0001
SEGAL_ADHD	ADHD	153	0.98	0.86	88.1%	93.0%	96.5%	<0.0001	<0.0001	<0.0001
SEGAL_ASD	Autism spectrum disorder	202	0.99	0.84	84.2%	92.6%	98.9%	<0.0001	<0.0001	<0.0001
SEGAL_BP	Bipolar disorder	228	0.97	0.73	84.9%	86.3%	98.7%	<0.0001	<0.0001	<0.0001
SEGAL_HCtest	Healthy control	1465	0.97	0.85	80.5%	92.8%	97.6%	<0.0001	<0.0001	<0.0001
SEGAL_MDD	Depression	161	0.95	0.68	52.7%	82.1%	97.9%	<0.0001	<0.0001	<0.0001
SEGAL_OCD	OCD	167	0.96	0.82	88.0%	92.0%	98.6%	<0.0001	<0.0001	<0.0001
SEGAL_SCZ	Schizophrenia	383	0.97	0.65	82.8%	90.0%	94.0%	<0.0001	<0.0001	<0.0001
SIDDIQI_ANXIETY	Anxiety-depression symptoms	451	0.56	0.82	54.9%	79.7%	-	<0.0001	<0.0001	<0.0001
SIDDIQI_DEPRESSION	Depression	461	0.28	0.71	16.8%	75.7%	-	<0.0001	0.0041	<0.0001
SIDDIQI_MSDEPRESSION	MS (depression)	281	0.44	0.75	44.8%	82.7%	-	<0.0001	<0.0001	<0.0001
SIDDIQI_POLITICAL	Political involvement	124	0.46	0.59	10.4%	71.8%	-	<0.0001	<0.0001	<0.0001
SIDDIQI_PTSD	PTSD (prevalence)	61	0.63	0.81	75.6%	86.1%	98.0%	<0.0001	0.0001	<0.0001
SIDDIQI_PTSD	PTSD (sLNM)	193	0.27	0.76	34.8%	90.1%	-	<0.0001	0.0035	<0.0001
SIHVONEN_AMUSIA	Amusia	24	0.80	0.79	65.1%	87.3%	93.2%	<0.0001	<0.0001	<0.0001
SOUTER_APHASIA	Aphasia (semantic)	23	0.44	0.67	21.3%	54.4%	-	<0.0001	<0.0001	<0.0001
STUBB_SUD	Substance abuse disorder	144	0.21	0.31	0.5%	84.8%	-	<0.0001	0.9285	<0.0001
SUN_EPILEPSY	Epilepsy	25	-0.23	0.55	13.6%	77.4%	83.6%	<0.0001	0.0052	0.0001
TAYLOR_TRANSDIAGNOSTIC	Transdiagnostic psychiatric disorders	193	0.14	0.54	0.6%	90.7%	-	<0.0001	0.9614	<0.0001
THEYS_STUTTERING	Neurogenic stuttering	20	0.66	0.55	22.6%	87.6%	94.1%	<0.0001	<0.0001	<0.0001
TRAPP_DEPRESSIONNEG	Depression (resilience)	13	-0.13	0.72	13.2%	82.4%	-	<0.0001	0.1119	0.0352
TRAPP_DEPRESSIONPOS	Depression (risk)	15	-0.03	0.34	2.2%	88.2%	-	<0.0001	0.4034	0.6395
WANG_AMNESIA	Transient global amnesia	51	-0.21	0.62	12.6%	77.7%	86.5%	<0.0001	0.0059	0.0002
YUAN_APNEA	Apnea	27	-0.09	0.22	4.0%	43.4%	93.2%	<0.0001	0.4951	0.1357
YUAN_RBD	Rapid eye movement sleep behavior disorder	19	-0.06	0.42	12.4%	68.3%	86.3%	<0.0001	0.0208	0.3161
ZARIFKAR_APHASIA	Aphasia	20	0.74	0.75	42.5%	85.6%	89.7%	<0.0001	<0.0001	<0.0001
ZARIFKAR_APRAXIA	Apraxia (eye-opening)	27	0.78	0.76	50.2%	84.1%	94.6%	<0.0001	<0.0001	<0.0001
ZOUKI_BLINK	Pathological urge (blink)	6	0.79	0.87	62.0%	89.5%	97.1%	<0.0001	<0.0001	<0.0001
ZOUKI_COUGH	Pathological urge (cough)	9	0.77	0.79	53.5%	84.7%	93.1%	<0.0001	<0.0001	<0.0001
ZOUKI_MICTURITION	Pathological urge (micturition)	19	0.61	0.82	27.4%	86.5%	93.7%	<0.0001	<0.0001	<0.0001
ZOUKI_SWALLOW	Pathological urge (swallow)	18	0.81	0.80	67.4%	90.6%	98.2%	<0.0001	<0.0001	<0.0001
ZOUKI_TICS	Tics (Tourette syndrome)	19	0.34	0.40	0.4%	87.9%	95.9%	<0.0001	0.2979	<0.0001

References

1. Siddiqi, S.H., *et al.* Brain stimulation and brain lesions converge on common causal circuits in neuropsychiatric disease. *Nat Hum Behav* **5**, 1707-1716 (2021).
2. Siddiqi, S.H., *et al.* A potential target for noninvasive neuromodulation of PTSD symptoms derived from focal brain lesions in veterans. *Nat Neurosci* **27**, 2231-2239 (2024).
3. Siddiqi, S.H., *et al.* Distinct Symptom-Specific Treatment Targets for Circuit-Based Neuromodulation. *Am J Psychiatry* **177**, 435-446 (2020).
4. Pines, A.R., *et al.* Mapping Lesions That Cause Psychosis to a Human Brain Circuit and Proposed Stimulation Target. *JAMA Psychiatry* (2025).
5. Kletenik, I., Gaudet, K., Prasad, S., Cohen, A.L. & Fox, M.D. Network Localization of Awareness in Visual and Motor Anosognosia. *Ann Neurol* **94**, 434-441 (2023).
6. Kletenik, I., *et al.* Multiple sclerosis lesions that impair memory map to a connected memory circuit. *J Neurol* **270**, 5211-5222 (2023).
7. Siddiqi, S.H., *et al.* Lesion network localization of depression in multiple sclerosis. *Nature Mental Health* **1**, 36-44 (2023).
8. Ji, G.J., *et al.* A generalized epilepsy network derived from brain abnormalities and deep brain stimulation. *Nat Commun* **16**, 2783 (2025).
9. Stubbs, J.L., *et al.* Heterogeneous neuroimaging findings across substance use disorders localize to a common brain network. *Nature Mental Health* **1**, 772-781 (2023).
10. Souter, N.E., *et al.* Mapping lesion, structural disconnection, and functional disconnection to symptoms in semantic aphasia. *Brain Struct Funct* **227**, 3043-3061 (2022).
11. Siddiqi, S.H., Balters, S., Zamboni, G., Cohen-Zimerman, S. & Grafman, J.H. Effects of focal brain damage on political behaviour across different political ideologies. *Brain* (2025).
12. Taylor, J.J., *et al.* A transdiagnostic network for psychiatric illness derived from atrophy and lesions. *Nat Hum Behav* **7**, 420-429 (2023).
13. Ji, G., *et al.* Distinct antidepressant therapies act on a common brain network. (2025).
14. Li, N., *et al.* A unified connectomic target for deep brain stimulation in obsessive-compulsive disorder. *Nat Commun* **11**, 3364 (2020).
15. Reich, M.M., *et al.* A brain network for deep brain stimulation induced cognitive decline in Parkinson's disease. *Brain* **145**, 1410-1421 (2022).
16. Joutsa, J., *et al.* Identifying therapeutic targets from spontaneous beneficial brain lesions. *Ann Neurol* **84**, 153-157 (2018).
17. Rios, A.S., *et al.* Optimal deep brain stimulation sites and networks for stimulation of the fornix in Alzheimer's disease. *Nat Commun* **13**, 7707 (2022).
18. Horn, A., *et al.* Optimal deep brain stimulation sites and networks for cervical vs. generalized dystonia. *Proc Natl Acad Sci U S A* **119**, e2114985119 (2022).
19. Joutsa, J., *et al.* Brain lesions disrupting addiction map to a common human brain circuit. *Nat Med* **28**, 1249-1255 (2022).
20. Darby, R.R., Horn, A., Cushman, F. & Fox, M.D. Lesion network localization of criminal behavior. *Proc Natl Acad Sci U S A* **115**, 601-606 (2018).
21. Darby, R.R., Joutsa, J., Burke, M.J. & Fox, M.D. Lesion network localization of free will. *Proc Natl Acad Sci U S A* **115**, 10792-10797 (2018).
22. LesionBank. www.lesionbank.org.
23. Jenkinson, M., Beckmann, C.F., Behrens, T.E., Woolrich, M.W. & Smith, S.M. Fsl. *Neuroimage* **62**, 782-790 (2012).
24. Segal, A., *et al.* Regional, circuit and network heterogeneity of brain abnormalities in psychiatric disorders. *Nat Neurosci* **26**, 1613-1629 (2023).
25. Schaefer, A., *et al.* Local-Global Parcellation of the Human Cerebral Cortex from Intrinsic Functional Connectivity MRI. *Cereb Cortex* **28**, 3095-3114 (2018).
26. Tian, Y., Margulies, D.S., Breakspear, M. & Zalesky, A. Topographic organization of the human subcortex unveiled with functional connectivity gradients. *Nat Neurosci* **23**, 1421-1432 (2020).
27. Kutsche, J., *et al.* Mapping Neuroimaging Findings of Creativity and Brain Disease Onto a Common Brain Circuit. *JAMA Netw Open* **8**, e2459297 (2025).
28. Peng, S., Xu, P., Jiang, Y. & Gong, G. Activation network mapping for integration of heterogeneous fMRI findings. *Nat Hum Behav* **6**, 1417-1429 (2022).
29. Trapp, N.T., *et al.* Large-scale lesion symptom mapping of depression identifies brain regions for risk and resilience. *Brain* **146**, 1672-1685 (2023).
30. Cash, R.F.H., Müller, V.I., Fitzgerald, P.B., Eickhoff, S.B. & Zalesky, A. Altered brain activity in unipolar depression unveiled using connectomics. *Nature Mental Health* **1**, 174-185 (2023).
31. Burke, M.J., *et al.* Mapping migraine to a common brain network. *Brain* **143**, 541-553 (2020).

32. Mollica, A., *et al.* The network-based underpinnings of persisting symptoms after concussion: a multimodal neuroimaging meta-analysis. *Nat Ment Health* **3**, 1276-1290 (2025).
33. Zouki, J.J., *et al.* Functional brain networks associated with the urge for action: Implications for pathological urge. *Neurosci Biobehav Rev* **163**, 105779 (2024).
34. Horn, A. & Kuhn, A.A. Lead-DBS: a toolbox for deep brain stimulation electrode localizations and visualizations. *Neuroimage* **107**, 127-135 (2015).
35. Cohen, A., Soussand, L., McManus, P. & Fox, M. GSP1000 Preprocessed Connectome. (Harvard Dataverse, 2020).
36. Van Essen, D.C., *et al.* The WU-Minn Human Connectome Project: an overview. *Neuroimage* **80**, 62-79 (2013).
37. van den Heuvel, M.P., *et al.* Proportional thresholding in resting-state fMRI functional connectivity networks and consequences for patient-control connectome studies: Issues and recommendations. *Neuroimage* **152**, 437-449 (2017).
38. Power, J.D., Barnes, K.A., Snyder, A.Z., Schlaggar, B.L. & Petersen, S.E. Spurious but systematic correlations in functional connectivity MRI networks arise from subject motion. *Neuroimage* **59**, 2142-2154 (2012).
39. Biswal, B.B., *et al.* Toward discovery science of human brain function. *Proc Natl Acad Sci U S A* **107**, 4734-4739 (2010).
40. Alexander-Bloch, A.F., *et al.* On testing for spatial correspondence between maps of human brain structure and function. *Neuroimage* **178**, 540-551 (2018).
41. Burt, J.B., Helmer, M., Shinn, M., Anticevic, A. & Murray, J.D. Generative modeling of brain maps with spatial autocorrelation. *Neuroimage* **220**, 117038 (2020).
42. Vos de Wael, R., *et al.* BrainSpace: a toolbox for the analysis of macroscale gradients in neuroimaging and connectomics datasets. *Commun Biol* **3**, 103 (2020).
43. Maslov, S. & Sneppen, K. Specificity and stability in topology of protein networks. *Science* **296**, 910-913 (2002).
44. Barabási, A.-L. & Albert, R. Emergence of scaling in random networks. *science* **286**, 509-512 (1999).
45. Newman, M.E. & Girvan, M. Finding and evaluating community structure in networks. *Phys Rev E Stat Nonlin Soft Matter Phys* **69**, 026113 (2004).
46. Darby, R.R., Joutsa, J. & Fox, M.D. Network localization of heterogeneous neuroimaging findings. *Brain* **142**, 70-79 (2019).
47. Fox, M.D. Mapping Symptoms to Brain Networks with the Human Connectome. *N Engl J Med* **379**, 2237-2245 (2018).
48. Fisher, R.A. On the mathematical foundations of theoretical statistics. *Philosophical transactions of the Royal Society of London. Series A, containing papers of a mathematical or physical character* **222**, 309-368 (1922).
49. Cohen, A.L. & Fox, M.D. Reply: The influence of sample size and arbitrary statistical thresholds in lesion-network mapping. *Brain* **143**, e41 (2020).
50. Wawrzyniak, M., Klingbeil, J., Zeller, D., Saur, D. & Classen, J. The neuronal network involved in self-attribution of an artificial hand: A lesion network-symptom-mapping study. *Neuroimage* **166**, 317-324 (2018).
51. Klingbeil, J., Wawrzyniak, M., Stockert, A., Karnath, H.O. & Saur, D. Hippocampal diaschisis contributes to anosognosia for hemiplegia: Evidence from lesion network-symptom-mapping. *Neuroimage* **208**, 116485 (2020).
52. Fox, M.D., Buckner, R.L., White, M.P., Greicius, M.D. & Pascual-Leone, A. Efficacy of transcranial magnetic stimulation targets for depression is related to intrinsic functional connectivity with the subgenual cingulate. *Biol Psychiatry* **72**, 595-603 (2012).
53. Horn, A., *et al.* Connectivity Predicts deep brain stimulation outcome in Parkinson disease. *Ann Neurol* **82**, 67-78 (2017).
54. Margulies, D.S., *et al.* Situating the default-mode network along a principal gradient of macroscale cortical organization. *Proc Natl Acad Sci U S A* **113**, 12574-12579 (2016).
55. Sporns, O. & Betzel, R.F. Modular Brain Networks. *Annu Rev Psychol* **67**, 613-640 (2016).
56. Weigand, A., *et al.* Prospective Validation That Subgenual Connectivity Predicts Antidepressant Efficacy of Transcranial Magnetic Stimulation Sites. *Biol Psychiatry* **84**, 28-37 (2018).
57. Seguin, C., *et al.* Neuroanatomical pathways of TMS therapy for depression. *medRxiv*, 2025.2002. 2010.25322034 (2025).
58. Gibson, M., *et al.* The Aphasia Recovery Cohort, an open-source chronic stroke repository. *Sci Data* **11**, 981 (2024).
59. Klingbeil, J., *et al.* Pathological laughter and crying: insights from lesion network-symptom-mapping. *Brain* **144**, 3264-3276 (2021).

60. Stockert, A., *et al.* Involvement of Thalamocortical Networks in Patients With Poststroke Thalamic Aphasia. *Neurology* **100**, e485-e496 (2023).
61. Kim, N.Y., *et al.* Network Effects of Brain Lesions Causing Central Poststroke Pain. *Ann Neurol* **92**, 834-845 (2022).
62. Kletenik, I., *et al.* Network Localization of Unconscious Visual Perception in Blindsight. *Ann Neurol* **91**, 217-224 (2022).
63. Germann, J., *et al.* Potential optimization of focused ultrasound capsulotomy for obsessive compulsive disorder. *Brain* **144**, 3529-3540 (2021).
64. Makhlouf, A.T., *et al.* Heterogeneous patterns of brain atrophy in schizophrenia localize to a common brain network. *Nature Mental Health* **3**, 19-30 (2025).
65. Peng, S., *et al.* Heterogenous brain activations across individuals localize to a common network. *Commun Biol* **7**, 1270 (2024).
66. Wang, Y., *et al.* Heterogeneous Brain Abnormalities in Schizophrenia Converge on a Common Network Associated With Symptom Remission. *Schizophr Bull* **50**, 545-556 (2024).
67. Fasano, A., Laganiere, S.E., Lam, S. & Fox, M.D. Lesions causing freezing of gait localize to a cerebellar functional network. *Ann Neurol* **81**, 129-141 (2017).
68. Li, Y., *et al.* A vertigo network derived from human brain lesions and brain stimulation. *Brain Commun* **5**, fcad071 (2023).
69. Siddiqi, S.H., *et al.* Causal network localization of brain stimulation targets for trait anxiety. *Res Sq* (2024).
70. Ferguson, M.A., *et al.* A neural network for religious fundamentalism derived from patients with brain lesions. *Proc Natl Acad Sci U S A* **121**, e2322399121 (2024).
71. Schaper, F., *et al.* Mapping Lesion-Related Epilepsy to a Human Brain Network. *JAMA Neurol* **80**, 891-902 (2023).
72. Li, N., *et al.* A Unified Functional Network Target for Deep Brain Stimulation in Obsessive-Compulsive Disorder. *Biol Psychiatry* **90**, 701-713 (2021).
73. Padmanabhan, J.L., *et al.* A Human Depression Circuit Derived From Focal Brain Lesions. *Biol Psychiatry* **86**, 749-758 (2019).
74. Rubinov, M., Kötter, R., Hagmann, P. & Sporns, O. Brain connectivity toolbox: a collection of complex network measurements and brain connectivity datasets. *NeuroImage* **47**, S169 (2009).
75. Sihvonen, A.J., *et al.* Focal Brain Lesions Causing Acquired Amusia Map to a Common Brain Network. *J Neurosci* **44** (2024).
76. Marek, S., *et al.* Reproducible brain-wide association studies require thousands of individuals. *Nature* **603**, 654-660 (2022).
77. Kim, N.Y., *et al.* Lesions causing hallucinations localize to one common brain network. *Mol Psychiatry* **26**, 1299-1309 (2021).
78. Joutsa, J., Corp, D.T. & Fox, M.D. Lesion network mapping for symptom localization: recent developments and future directions. *Curr Opin Neurol* **35**, 453-459 (2022).
79. Sperber, C. & Dadashi, A. The influence of sample size and arbitrary statistical thresholds in lesion-network mapping. *Brain* **143**, e40 (2020).
80. Vasa, F. & Misic, B. Null models in network neuroscience. *Nat Rev Neurosci* **23**, 493-504 (2022).
81. Theys, C., *et al.* Localization of stuttering based on causal brain lesions. *Brain* **147**, 2203-2213 (2024).
82. Hagberg, A., Swart, P.J. & Schult, D.A. Exploring network structure, dynamics, and function using NetworkX. (Los Alamos National Laboratory (LANL), Los Alamos, NM (United States), 2008).
83. Ganos, C., *et al.* A neural network for tics: insights from causal brain lesions and deep brain stimulation. *Brain* **145**, 4385-4397 (2022).
84. Zouki, J.J., *et al.* Mapping a network for tics in Tourette syndrome using causal lesions and structural alterations. *Brain Commun* **5**, fcad105 (2023).
85. Corp, D.T., *et al.* Network localization of cervical dystonia based on causal brain lesions. *Brain* **142**, 1660-1674 (2019).
