# Supplementary material for: Investigating the methodological foundation of lesion network mapping
Source: Nat Neurosci. 2026 Jan 15;29(5):1237–47. doi: 10.1038/s41593-025-02196-7 (PMC13156034; doi:10.1038/s41593-025-02196-7)
Supplement: Supplementary file 2 — Reporting Summary [file 41593_2025_2196_MOESM2_ESM.pdf]

nature portfolio

Corresponding author(s): Martijn van den Heuvel
martijn.vanden.heuvel@vu.nl

Last updated by author(s): 10/12/2025

Reporting Summary

Nature Portfolio wishes to improve the reproducibility of the work that we publish. This form provides structure for consistency and transparency in reporting. For further information on Nature Portfolio policies, see our Editorial Policies and the Editorial Policy Checklist.

Statistics

For all statistical analyses, confirm that the following items are present in the figure legend, table legend, main text, or Methods section.

n/a	Confirmed	
☐	☒	The exact sample size (*n*) for each experimental group/condition, given as a discrete number and unit of measurement
☐	☒	A statement on whether measurements were taken from distinct samples or whether the same sample was measured repeatedly
☐	☒	The statistical test(s) used AND whether they are one- or two-sided<br>*Only common tests should be described solely by name; describe more complex techniques in the Methods section.*
☐	☒	A description of all covariates tested
☐	☒	A description of any assumptions or corrections, such as tests of normality and adjustment for multiple comparisons
☐	☒	A full description of the statistical parameters including central tendency (e.g. means) or other basic estimates (e.g. regression coefficient) AND variation (e.g. standard deviation) or associated estimates of uncertainty (e.g. confidence intervals)
☐	☒	For null hypothesis testing, the test statistic (e.g. *F*, *t*, *r*) with confidence intervals, effect sizes, degrees of freedom and *P* value noted<br>*Give P values as exact values whenever suitable.*
☒	☐	For Bayesian analysis, information on the choice of priors and Markov chain Monte Carlo settings
☒	☐	For hierarchical and complex designs, identification of the appropriate level for tests and full reporting of outcomes
☐	☒	Estimates of effect sizes (e.g. Cohen's *d*, Pearson's *r*), indicating how they were calculated

Our web collection on statistics for biologists contains articles on many of the points above.

Software and code

Policy information about availability of computer code

Data collection	Neuroimaging data was from available open-source datasets,<br>additional lesion masks were manually segmented from images from published studies.
Data analysis	Lesion Network Mapping was conducted using<br>Lead-DBS toolbox (v3.1; https://www.lead-dbs.org/). Datasets were analyzed using MATLAB, and Python with<br>two-sided independent t-tests and regression analysis were done using Scipy package (https://scipy.org/)<br>and Visualization of brain plots with Nibabel package (https://nipy.org/nibabel/).

For manuscripts utilizing custom algorithms or software that are central to the research but not yet described in published literature, software must be made available to editors and reviewers. We strongly encourage code deposition in a community repository (e.g. GitHub). See the Nature Portfolio guidelines for submitting code & software for further information.

Data

Policy information about availability of data

All manuscripts must include a data availability statement. This statement should provide the following information, where applicable:

- Accession codes, unique identifiers, or web links for publicly available datasets
- A description of any restrictions on data availability
- For clinical datasets or third party data, please ensure that the statement adheres to our policy

All data used in the present study are publicly available. The preprocessed normative functional connectivity time-series from the GSP1000 dataset are available from [https://doi.org/10.7910/DVN/ILXIKS] and the Lead-DBS toolbox from [https://www.lead-dbs.org]. Neuroimaging data from the Human Connectome Project are available at [www.humanconnectome.org]. LNM maps used in this study are available from [https://neurovault.org] and GitHub [https://github.com]. Lesion masks associated with amnesia, hypersomnia, insomnia, neglect syndrome, and Alice in Wonderland syndrome are available from [https://www.lesionbank.org/]. All other reported lesions or LNM data are directly available from the referenced papers.

Research involving human participants, their data, or biological material

Policy information about studies with human participants or human data. See also policy information about sex, gender (identity/presentation), and sexual orientation and race, ethnicity and racism.

Reporting on sex and gender	NA. Similar data as reported in the original Lesion Network Mapping studies was used.
Reporting on race, ethnicity, or other socially relevant groupings	NA. Similar data as reported in the original Lesion Network Mapping studies was used.
Population characteristics	Patients. Similar data as reported in the original Lesion Network Mapping studies was used.
Recruitment	NA, all data included open source data
Ethics oversight	NA, concerns a mathematical examination

Note that full information on the approval of the study protocol must also be provided in the manuscript.

Field-specific reporting

Please select the one below that is the best fit for your research. If you are not sure, read the appropriate sections before making your selection.

☒ Life sciences ☐ Behavioural & social sciences ☐ Ecological, evolutionary & environmental sciences

For a reference copy of the document with all sections, see nature.com/documents/nr-reporting-summary-flat.pdf

Life sciences study design

All studies must disclose on these points even when the disclosure is negative.

Sample size	The objective was to examine the validity of the Lesion Network Mapping method. The study analyzed openly available and previously published data, with the same sample and sample sizes similar as in the published studies.
Data exclusions	All openly available and previously published case studies were included in the analysis, as the aim was to assess the reproducibility and validity of the methodology under investigation. Exclusion criteria were not applicable.
Replication	Experiments consisted of replicating neuroimaging findings applying Lesion Network Mapping.<br>Experiments were conducted with original and simulated data. Validation and sensitivity analyses included multiple brain resolutions (atlas-based, voxel-based) and multiple normative connectome datasets.
Randomization	NA
Blinding	NA

Reporting for specific materials, systems and methods

We require information from authors about some types of materials, experimental systems and methods used in many studies. Here, indicate whether each material, system or method listed is relevant to your study. If you are not sure if a list item applies to your research, read the appropriate section before selecting a response.

Materials & experimental systems

n/a	Involved in the study
☒	☐ Antibodies
☒	☐ Eukaryotic cell lines
☒	☐ Palaeontology and archaeology
☒	☐ Animals and other organisms
☒	☐ Clinical data
☒	☐ Dual use research of concern
☒	☐ Plants

Methods

n/a	Involved in the study
☒	☐ ChIP-seq
☒	☐ Flow cytometry
☐	☒ MRI-based neuroimaging

Plants

Seed stocks

Report on the source of all seed stocks or other plant material used. If applicable, state the seed stock centre and catalogue number. If plant specimens were collected from the field, describe the collection location, date and sampling procedures.

Novel plant genotypes

Describe the methods by which all novel plant genotypes were produced. This includes those generated by transgenic approaches, gene editing, chemical/radiation-based mutagenesis and hybridization. For transgenic lines, describe the transformation method, the number of independent lines analyzed and the generation upon which experiments were performed. For gene-edited lines, describe the editor used, the endogenous sequence targeted for editing, the targeting guide RNA sequence (if applicable) and how the editor was applied.

Authentication

Describe any authentication procedures for each seed stock used or novel genotype generated. Describe any experiments used to assess the effect of a mutation and, where applicable, how potential secondary effects (e.g. second site T-DNA insertions, mosiacism, off-target gene editing) were examined.

Magnetic resonance imaging

Experimental design

Design type: Lesion Network Mapping

Design specifications: NA

Behavioral performance measures: NA

Acquisition

Imaging type(s): T1-weighted imaging, resting-state functional MRI

Field strength: 1.3, 3T

Sequence & imaging parameters: Sequence and imaging parameters were described in original publications of datasets.

Area of acquisition: Whole Brain

Diffusion MRI: ☐ Used ☒ Not used

Preprocessing

Preprocessing software: Lesion masks were mapped to functional maps using Lead-DBS toolbox.

Normalization: Freesurfer normalized T1-weighted anatomical data extracting brain tissue, denoising, and bias field correction. GSP1000 FMRI data as provided by Lead-DBS.

Normalization template: MNI152. Native patient images were projecting into standard stereotactic (MNI) space.

Noise and artifact removal: Sensitivity analyses were performed for exclusion of small lesions and small lesion sets.

Volume censoring: NA

Statistical modeling & inference

Model type and settings: Lesion Network Mapping

Effect(s) tested: Pearson correlation, linear regression, permutation testing

Specify type of analysis: ☒ Whole brain ☐ ROI-based ☐ Both

Statistic type for inference (See Eklund et al. 2016)	Statistical testing using sensitivity, specificity and conjunction was conducted.
Correction	FWE, Bonferroni, spatial-autocorrelation models (Spin-model and BrainSMASH).

Models & analysis

n/a	Involved in the study
☐	☒ Functional and/or effective connectivity
☐	☒ Graph analysis
☒	☐ Multivariate modeling or predictive analysis

Functional and/or effective connectivity	Functional connectivity was derived as correlation; as similar to the LNM studies tested.
Graph analysis	Weighted and binary graphs were examined. Scale-free, modular and randomized (degree preserved and otherwise), and spin-model permutation analysis was used.
